# The Potential Fungal Pathogens of *Euonymus japonicus* in Beijing, China

**DOI:** 10.3390/jof9020271

**Published:** 2023-02-18

**Authors:** Lu Lin, Meng Pan, Hong Gao, Chengming Tian, Xinlei Fan

**Affiliations:** 1 The Key Laboratory for Silviculture and Conservation of Ministry of Education, Beijing Forestry University, Beijing 100083, China; 2 The Key Laboratory for Biodiversity Science and Ecological Engineering, College of Life Sciences, Beijing Normal University, Beijing 100101, China

**Keywords:** *Euonymus japonicus*, fungal pathogen, new fungal species, phylogeny, taxonomy

## Abstract

*Euonymus japonicus* tolerates the dry and frigid climate of Beijing, China, and effectively filters out particles during the winter. However, fungal infestation frequently causes extreme illness and can even lead to shrub death. In this study, 104 diseased *E. japonicus* specimens were collected from seven districts in Beijing. Seventy-nine isolates were identified as 22 fungal species in seven genera. The species were *Aplosporella hesperidica*, *A. javeedii*, *A. prunicola*, *Botryosphaeria dothidea*, *Colletotrichum aenigma*, *Co. euonymi*, *Co. euonymicola*, *Co. gloeosporioides*, *Cytospora ailanthicola*, *C. albodisca*, *C. diopuiensis*, *C. discotoma*, *C. elaeagni*, *C. euonymicola*, *C. euonymina*, *C. haidianensis*, *C. leucostoma*, *C. sophorae*, *C. zhaitangensis*, *Diaporthe eres*, *Dothiorella acericola*, and *Pestalotiopsis chaoyangensis*. On the basis of morphological and phylogenetic analyses, *Colletotrichum euonymi*, *Co. euonymicola*, *Cytospora zhaitangensis*, and *Pestalotiopsis chaoyangensis* were introduced as novel species. *Colletotrichum euonymi*, *Co. euonymicola*, and *Pestalotiopsis chaoyangensis* were subsequently confirmed as pathogens of *E. japonicus* leaves by pathogenicity testing. This study provides an important assessment of the fungi associated with diseases of *E. japonicus* in Beijing, China.

## 1. Introduction

**Figure 1:**
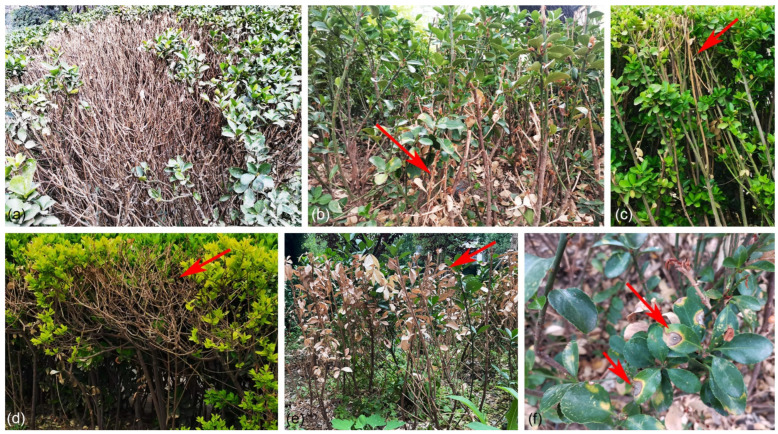
Diseased *Euonymus japonicus* in Beijing: (**a**–**e**) Symptoms of branches dieback; (**f**) Symptoms of leaf spots.

The family Celastraceae, which includes 96 genera and over 1350 species, is extensively distributed in tropical, subtropical, and temperate climates as evergreen or deciduous trees, shrubs, or vine shrubs [1]. *Euonymus japonicus*, also called Japanese spindle, is one of the most prevalent and important species in Celastraceae in northern Chinese cities such as Beijing, the capital of China [2]. As a species of evergreen shrub, *E. japonicus* has strong resistance to the dry and cold conditions in Beijing and can efficiently reduce particulate matter in winter [2]. Furthermore, roots, stems, and leaves of the shrub have a high capacity to enrich heavy metals [3]. However, Japanese spindle in Beijing is frequently extremely ill and even dies because of fungal infestation (Figure 1).

Fungi from many different taxa are associated with diseases of the same host plant species or genus. Raza et al. [4] described one new genus and 32 new species of culturable fungi associated with sugarcane disease in southern China, and Crous et al. [5] described seven new genera and 15 new species as foliar fungal pathogens of eucalypts. Accurate identification of pathogenic fungi provides a good theoretical foundation for the control of plant diseases. However, because fungi associated with fungal diseases of Japanese spindle in Beijing have not been systematically and extensively studied, effective disease prevention is difficult. Therefore, the variety of fungal species associated with Japanese spindle diseases in Beijing was examined in this study. In the study, 79 fungal isolates were classified as seven genera (*Aplosporella*, *Botryosphaeria*, *Collectotrichum*, *Cytospora*, *Diaporthe*, *Dothiorella*, and *Pestalotiopsis*) in four orders (Amphisphaeriales, Botryosphaeriales, Diaporthales, and Glomerellales).

*Aplosporella*, *Botryosphaeria*, and *Dothiorella* are genera in Botryosphaeriales and Dothideomycetes [6]. Botryosphaeriales is an order that includes many pathogens to various hosts [7,8]. Currently, six families (Aplosporellaceae, Botryosphaeriaceae, Melanopsaceae, Phyllostictaceae, Planistromellaceae, and Saccharataceae) and 33 genera are accepted in Botryosphaeriales [6,9]. *Aplosporella* is in Aplosporellaceae, and *Botryosphaeria* and *Dothiorella* are in Botryosphaeriaceae. The two families can be distinguished by the locules, with Aplosporellaceae multiloculate and Botryosphaeriaceae uniloculate [8]. Conidia of *Botryosphaeria* are aseptate, whereas those of *Dothiorella* are septate [10]. Cash [11] recorded *A. euonymi* on *Euonymus atropurpureus*. Hanlin [12] and Reid [13] reported that *B. dothidea* and *B. quercuum* were associated with *E. americanus* and *E. japonicus*, respectively. Dissanayake et al. [14] reported that *Do. sarmentorum* was associated with *E. europaeus*. 

*Colletotrichum* is in Glomerellales, Sordariomycetes. The genus comprises 15 species complexes, i.e., the species complexes of *Co. acutatum*, *Co. agaves*, *Co. boninense*, *Co. caudatum*, *Co. dematium*, *Co. destructivum*, *Co. dracaenophilum*, *Co. gigasporum*, *Co. gloeosporioides*, *Co. graminicola*, *Co. magnum*, *Co. orbiculare*, *Co. orchidearum*, *Co. spaethianum*, and *Co. truncatum* [15,16]. Internal transcribed spacer region (ITS) is always used to assign *Colletotrichum* species to species complexes [16,17]. Liu et al. [16] combined ITS, *act*, *chs-1*, *gapdh*, *his3*, and *tub2* sequences to construct a phylogenetic tree of *Colletotrichum* species. In addition, ITS, *apn2*, Mat1/Apn2, and *sod2* sequences were combined in phylogenetic analyses of the *Co. caudatum* species complex, and ITS, *act*, *chs*, *sod2*, and *tub2* sequences were combined in phylogenetic analyses of the *Co. graminicola* species complex [16]. *Colletotrichum boninense*, *Co. gigasporum*, *Co. gloeosporioides*, *Co. griseum*, *Co. karsti*, and *Co. siamense* have been recorded on host *Euonymus* [18,19,20,21,22].

*Cytospora* and *Diaporthe* are genera in Diaporthales, Sordariomycetes. Diaporthales includes many pathogens that cause dieback and canker disease in various hosts [23,24,25,26,27,28,29]. *Cytospora euonymi*, *C. euonymicola*, *C. euonymina*, *C. haidianensis*, and *C. leucostoma* have been recorded on host *Euonymus* [30,31,32,33]. *Diaporthe eres*, *D. euonymi*, *D. laschii*, and *D. pardalota* have been recorded on host *Euonymus* [34,35,36,37].

*Pestalotiopsis* is in Amphisphaeriales, Sordariomycetes. Steyaert [38] distinguished the genus from *Pestalotia* on the basis of its morphological features (five-celled, clostridial conidia with colored cells in the middle three cells and colorless apical cells, and one to several apical appendages). *Pestalotiopsis* is a well-known phytopathogenic genera. Jiang et al. [39] introduced 10 new species of *Pestalotiopsis* from Fagaceae leaves in China. *Pestalotiopsis breviseta*, *P. caroliniana*, *P. clavata*, *P. diospyri*, *P. gracilis*, *P. neglecta*, and *P. planimi* have been recorded on host *Euonymus* [40,41,42,43].

During investigations of the diversity of fungal species that cause diseases of *E. japonicus*, several ascomycetous taxa associated with various disease symptoms were collected in Beijing. The objectives of this study are to (1) investigate fungal diseases on *E. japonicus* in Beijing, (2) identify the fungal species isolated from *E. japonicus*, and (3) test the pathogenicity of the novel species identified.

## 2. Materials and Methods

### 2.1. Sampling and Isolation

During 2020 to 2021, a survey was conducted in seven districts (Chaoyang, Daxing, Fengtai, Haidian, Mentougou, Shijingshan, and Xicheng) in Beijing, China. A total of 104 specimens (67 branches and 37 leaves) affected with different symptoms were collected. Isolates from leaves were obtained using tissue isolation methods. Leaf spots were cut into small pieces (0.2 × 0.2 cm) and placed on potato dextrose agar (PDA, 200 g potato, 20 g glucose, 20 g agar, and 1000 mL water) plates and incubated at 25 °C after surface sterilisation (1 min in 75% ethanol, 3 min in 1.25% sodium hypochlorite, then rinsed in distilled water and blotted on dry sterile filter paper). Fruiting bodies on diseased branches were shaved off the surface with a sterile blade after surface sterilisation, then the mucoid spore mass from conidiomata was put onto a PDA culture medium and incubated at 25 °C in darkness until spores germinated. Single germinating conidia were transferred onto fresh PDA plates. Hyphal tips were cut and transferred to a new PDA plate twice to obtain a pure culture for further study. Specimens are preserved at the working collection of X.L. Fan (CF) housed at the Beijing Forestry University (BJFC). Cultures of taxonomic novelties are deposited at the China Forestry Culture Collection Centre (CFCC). 

### 2.2. DNA Extraction, PCR Amplification, and Sequencing

The modified CTAB method [44] was used to extract total genomic DNA from mycelium on PDA. The PCR mixture consisted of 10 μL TopTaq™ Master Mix, 7 μL nuclease-free H_2_O, 1 μL of each primer, and 1 μL DNA samples were made up to the final volume of 20 μL. Partial gene sequences were amplified by polymerase chain reaction (PCR) using the primer sets ITS1/ITS4 for ITS region [45], EF-728F/EF-986R for *tef1-α* [46], Bt-2a/Bt-2b for *tub2* [47], fRPB2-5F/fRPB2-7cR for *rpb2* [48], ACT-512F/ACT-783R for *act* [46], CHS-79F/CHS-345R for *chs1* [46], GDF1/GDF2 for *gaphd* [49], CAL-228F/CAL-737R for *cal* [46], and CYLH3F/H3-1b [47,50] for *his3*. The genes used in different genera and the PCR conditions are listed in Table 1.

PCR products were electrophoresed in 1% agarose gel, and the DNA was sequenced by the SinoGenoMax Company Limited (Beijing, China). The forward and reverse reads were assembled by Seqman v. 7.1.0 in the DNASTAR Lasergene core suite software (DNASTAR Inc., Madison, WI, USA). All sequences obtained in this study were submitted to GenBank (Table S1).

### 2.3. Phylogenetic Analyses

The sequence datasets used in this study were based on Lin et al. [51,52] for *Cytospora*, Liu et al. [16] for *Colletotrichum*, Jiang et al. [39] for *Pestalotiopsis*, and Zhang et al. [6] for Botryosphariales, deleting the overly repetitive isolates of the same species and supplementing them with other sequences obtained from GenBank (Table S1). Sequence alignments of the individual loci were performed in MAFFT v. 6 [53] and adjusted by MEGA v. 6.0 [54]. Ambiguous regions were excluded from alignments. For the genus *Colletotrichum*, the ITS tree, including 15 species complexes, was first used for inferring delimitation to the species complex level before multi-locus phylogenetic analyses. Maximum Likelihood (ML) and Bayesian Inference (BI) were used for phylogenetic analyses of both each individual loci and the concatenated genes alignments. ML and BI analyses were computed using PhyML v. 3.0 [55] and MrBayes v. 3.1.2 [56], respectively. For ML analyses, GTR + GAMMA model of site substitution with 1000 bootstrap was set. For BI analyses, the best-fit evolutionary models for each partitioned locus were estimated in MrModeltest v. 2.4 [57]. BI analyses with a four simultaneous Markov Chain Monte Carlo (MCMC) were computed from random trees for 1,000,000 generations and sampled every 1000 generations, and the burn-in was set to 0.25. The resulting trees were viewed in Figtree v. 1.3.1 [58]. The multi-locus sequence alignments were deposited in TreeBASE 29991.

### 2.4. Morphology

For the isolates isolated from diseased branches, the fruiting bodies on the specimens corresponding to the isolates were used for morphological observation. For the isolates isolated from leaf spots, reproductive structures formed on PDA were used for morphological observation. The structure and size of conidiomata were photographed using the Leica stereomicroscope (M205 FA) (Leica Microsystems, Wetzlar, Germany). Over 30 conidiomata were sectioned, and 50 conidia were selected randomly to measure their lengths and widths using a Nikon Eclipse 80 i microscope (Nikon Corporation, Tokyo, Japan) equipped with a Nikon digital sight DS-Ri2 high-definition color camera with differential interference contrast (DIC). Colony color on PDA were described according to the color charts of Rayner [59].

### 2.5. Pathogenicity Tests

Healthy branches and leaves of *E. japonicus* collected from Beijing Forestry University (116°20′28″ E, 40°0′8″ N) were used to confirm the pathogenicity of the ex-holotype of the four novel species (isolated from branches: *C. zhaitangensis*; and isolated from leaves: *Co. euonymi*, *Co. euonymicola*, and *P. chaoyangensis*). Two-year-old branches were inoculated with *C. zhaitangensis* (CFCC 56227), and one-year-old leaves were inoculated with *Co. euonymi* (CFCC 55542), *Co. euonymicola* (CFCC 55486), and *P. chaoyangensis* (CFCC 55549). 

Branches were cut to 20-cm lengths, with the bottoms submerged in water and tips sealed with sealing film. To inoculate branches, a hole punch with 5-mm diameter and approximately 0.5-mm thickness was used to scald branches 10 cm from the tip. A 14-day culture block of the same size was attached to the wounds. Branches were then wet with skimming cotton moistened with sterile water and covered in sealing film. To inoculate leaves, a sterile inoculation needle pierced the leaves five to seven times, and 4-mm-diameter 14-day culture blocks were placed on the wounds. Branch and leaf inoculations were incubated at 25 °C and 70% humidity. Six replicates were prepared for each isolate, and a sterile PDA plug served as the control. Experiments were conducted twice. To fulfil Koch’s postulates, re-isolations were made from lesions to compare the morphological features and DNA sequences with those of the original isolates.

R 4.2.2 with the packages “ggplot2” [60] and “ggpubr” [61] was used to analyse pathogenicity data and output figure. Data were analysed using Tukey’s honestly significant difference (HSD) test (*α* = 0.05) by one-way analysis of variance (ANOVA).

## 3. Results

### 3.1. Disease Investigation and Isolation

**Figure 2:**
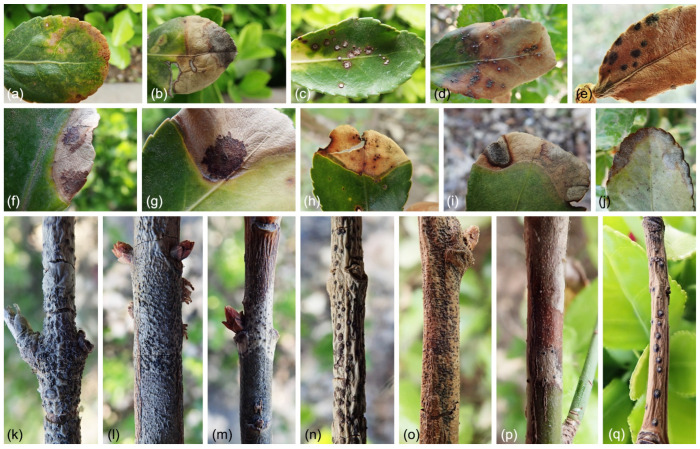
Different symptoms of the diseases of *Euonymus japonicus*: (**a**–**j**) Leaves spots with different symptoms; (**k**–**q**) Different morphological characteristics of reproductive structure on diseased branches.

Symptoms on leaves of infected plants included necrotic patches with tiny, black dot-like infection centers. Branch tips were depressed and dead and spread downward. Black reproductive structures appeared on dead portions of the branches (Figure 2). A total of 79 isolates were obtained, with 26 isolates from Chaoyang, 15 from Haidian, 14 from Mentougou, 10 from Shijingshan, five from Daxing, five from Xicheng, and four from Fengtai districts.

### 3.2. Phylogenetic Analyses

**Figure 3:**
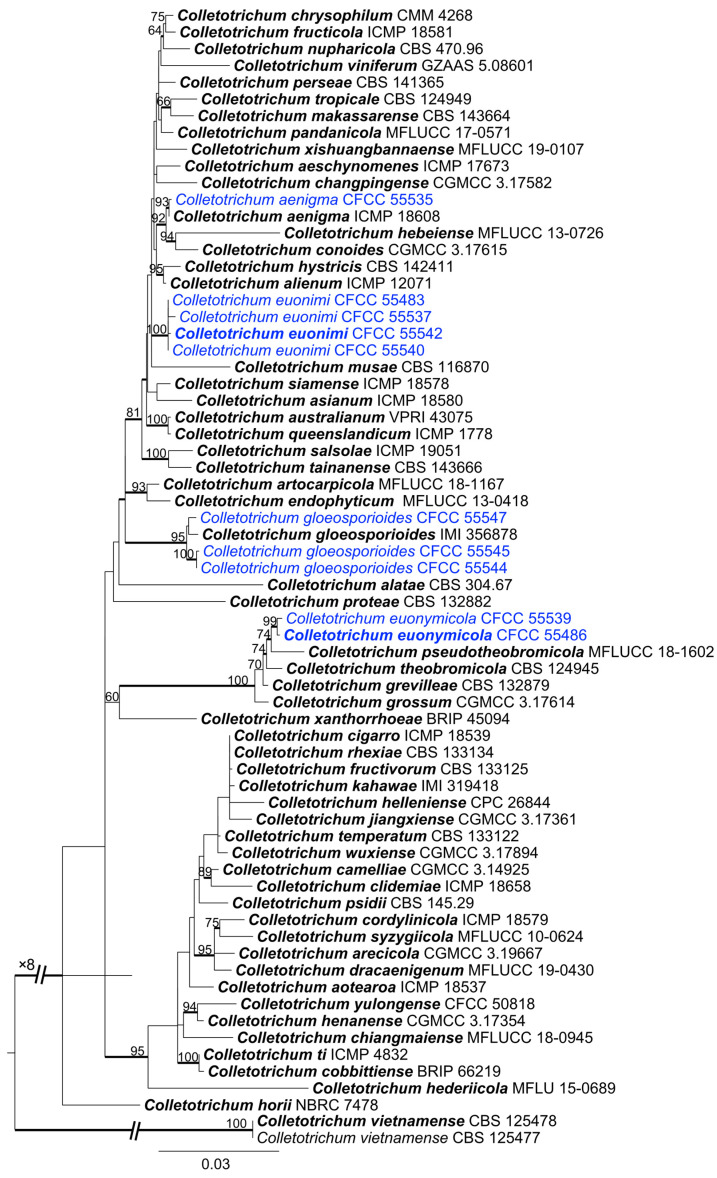
Phylogram of *Colletotrichum gloeosporioides* complex based on maximum likelihood (ML) analysis of the dataset of combined ITS, *act*, *tub2*, *chs1*, and *gaphd* genes. ML bootstrap support values above 70% are shown near nodes. Thickened branches represent posterior probabilities above 0.95 from BI. Ex-type isolates are in bold. Isolates highlighted with blue colours were obtained in this study.

**Figure 4:**
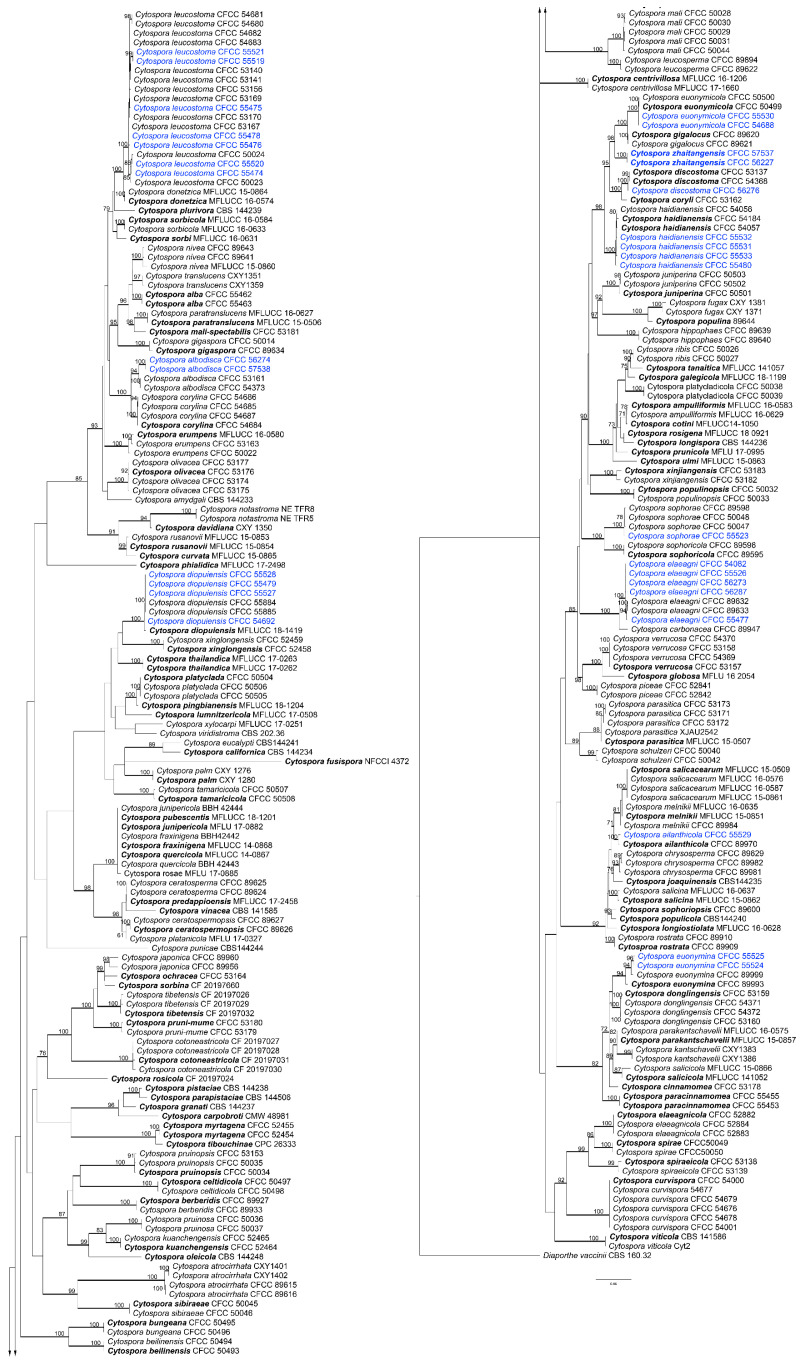
Phylogram of *Cytospora* based on maximum likelihood (ML) analysis of the dataset of combined ITS, *act*, *rpb2*, *tef1-α*, and *tub2* genes. ML bootstrap support values above 70% are shown near nodes. Thickened branches represent posterior probabilities above 0.95 from BI. Ex-type isolates are in bold. Isolates highlighted with blue colours were obtained in this study.

**Figure 5:**
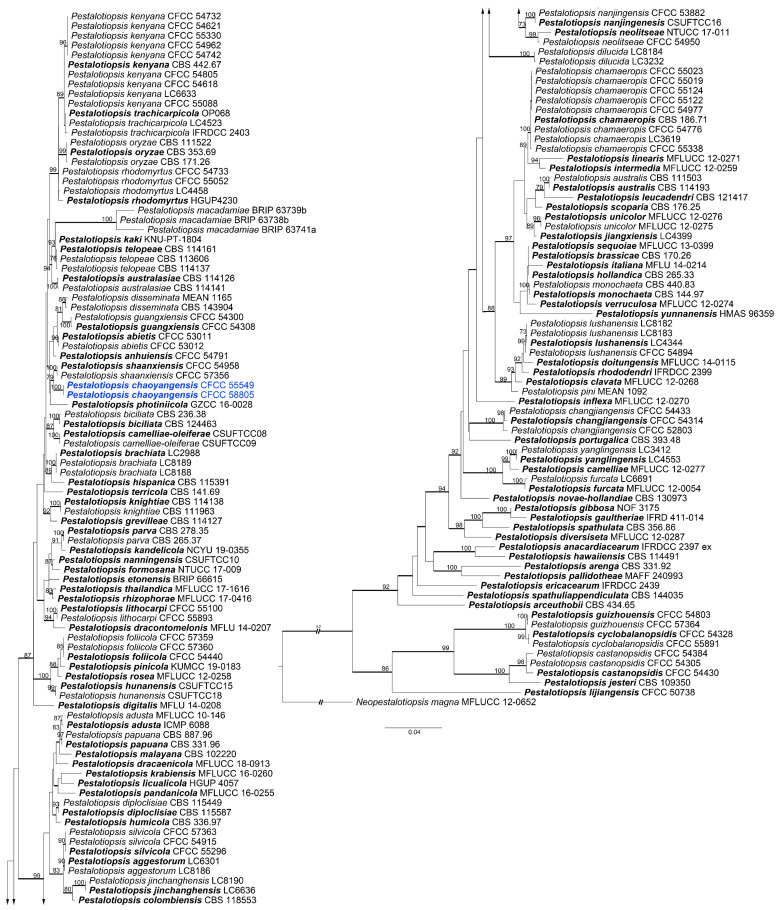
Phylogram of *Pestalotiopsis* based on maximum likelihood (ML) analysis of the dataset of combined ITS, *tef1-α*, and *tub2* genes. ML bootstrap support values above 70% are shown near nodes. Thickened branches represent posterior probabilities above 0.95 from BI. Ex-type isolates are in bold. Isolates highlighted with blue colours were obtained in this study.

The best-fit models used in Bayesian analyses and the statistics of ML trees are presented in Table 2 and Table 3, respectively. The ML trees with ML bootstrap support values and posterior probabilities are shown in Figure 3, Figure 4 and Figure 5 and Figures S1–S5. The results of Bayesian analyses did not significantly differ from those of ML trees.

The 79 isolates clustered into seven genera, i.e., *Aplosporella*, *Botryosphaeria*, *Collectotrichum*, *Cytospora*, *Diaporthe*, *Dothiorella*, and *Pestalotiopsis*. Eight isolates in the genus *Aplosporella* were clustered in three clades with *A. hesperidica*, *A. javeedii*, and *A. prunicola* (Figure S1). Twenty-one isolates in the genus Botryosphaeria clustered with *B. dothidea* (Figure S2). The ten isolates in Colletotrichum all clustered in the *Co. gloeosporioides* complex based on the ITS tree (Figure S3). *Multilocus* analyses in the *Co. gloeosporioides* complex showed that the ten isolates clustered in four clades (Figure 3). Isolate CFCC 55535 clustered with *Co. aenigma*, and three isolates (CFCC 55544, 55545 and 55547) clustered with *Co. gloeosporioides*. Four isolates (CFCC 55483, 55537, 55540, and 55542) and two isolates (CFCC 55486 and 55539) formed two distinct branches. A total of 31 isolates in the genus *Cytospora* clustered in 11 clades, with 10 known species (*C. ailanthicola*, *C. albodisca*, *C. diopuiensis*, *C. discotoma*, *C. elaeagni*, *C. euonymicola*, *C. euonymina*, *C. haidianensis*, *C. leucostoma*, and *C. sophorae*), and one forming a distinct branch (CFCC 56227 and 57537) (Figure 4). Three isolates in *Diaporthe* were clustered in the *D. alnea* species complex (Figure S4). Four isolates in *Dothiorella* clustered with *Do. acericola* (Figure S5). Two isolates in *Pestalotiopsis* (CFCC 55549 and 58805) were placed in a distinct clade (Figure 5).

### 3.3. Taxonomy

**Dothideomycetes** O.E. Erikss. and Winka, Myconet 1 (1): 5, 1997.

**Botryosphaeriales** C.L. Schoch, Crous and Shoemaker Mycologia 98 (6): 1050, 2007.

***Aplosporella*** Speg., Anal. Soc. cient. argent. 10 (5–6): 158, 1880.

***Aplosporella hesperidica*** Speg., Anal. Soc. cient. argent. 13 (1): 18, 1882.

*Description*: See Mapook et al. [62] and Dissanayake et al. [7].

*Materials examined*: China. Beijing City, Haidian District, Shucun Park, 116°17′55″ E, 40°0′53″ N, from branches of *Euonymus japonicus*, H. Gao and X.L. Fan, 7 May 2021 (BJFC CF20220104, living culture CFCC 55554).

*Notes*: *Aplosporella hesperidica* was first discovered by Spegazzini [63] on Citrus × aurantium in Argentina. The isolate CFCC 55554 clustered with *Aplosporella hesperidica* CBS 732.79 and *A. hesperidica* CBS 208.37 in the present phylogenetic analysis and showed 100% similarity in ITS sequence. Therefore, CFCC 55554 is identified as *Aplosporella hesperidica* according to phylogenetic analyses. A new host record from *Euonymus japonicus* is provided here.

***Aplosporella javeedii*** Jami, Gryzenh., Slippers and M.J. Wingf., Fungal Biol. 118 (2): 174, 2013.

*Descriptions*: See Jami et al. [64] and Fan et al. [65].

*Materials examined*: China. Beijing City, Chaoyang District, Sun Palace South Street, 116°26′57″ E, 39°58′10″ N, from branches of *Euonymus japonicus*, H. Gao and X.L. Fan, 21 April 2021 (BJFC CF20220101, living culture CFCC 55489). Mentougou District, Shitan Road, 116°6′31″ E, 39°55′36″ N, from branches of *Euonymus japonicus*, H. Gao and X.L. Fan, 25 May 2021 (BJFC CF20220102, living culture CFCC 55553). Haidian District, Shucun Park, 116°17′55″ E, 40°0′53″ N, from branches of *Euonymus japonicus*, H. Gao and X.L. Fan, 7 June 2021 (BJFC CF20220103, living culture CFCC 55555).

*Notes*: *Aplosporella javeedii* was introduced by Jami et al. [64] from a healthy wood section of *Celtis Africana*. It has been reported on 14 different hosts in 10 families, such as Rhamnaceae, Rosaceae, and Cupressaceae [65,66,67,68,69]. The isolates CFCC 55489, 55553, and 55555 were grouped together with *A. javeedii* (CFCC 50052 and 50054) with high statistical support (ML/BI = 99/1). The conidia of CFCC 55489 on PDA are aseptate, ellipsoid to oblong, smooth, ends rounded, initial hyaline, becoming brown when mature, 19.5–23.0 × 6.5–8.5 µm, which overlap with the morphological characteristics described by Jami et al. [64]. Therefore, three isolates in this study are identified as *A. javeedii* based on phylogenetic and morphological analyses.

***Aplosporella prunicola*** Damm and Crous, Fungal Divers. 27 (1): 39, 2007.

*Descriptions*: See Damm et al. [70].

*Material examined*: China. Beijing City, Chaoyang District, Sanyuanli, 116°26′59″ E, 39°57′10″ N, from branches of *Euonymus japonicus*, H. Gao and X.L. Fan, 20 April 2021 (BJFC CF20220105, living culture CFCC 55550). Mentougou District, Lisichen Park, 116°6′33″ E, 39°55′39″ N, from branches of *Euonymus japonicus*, H. Gao and X.L. Fan, 7 May 2021 (BJFC CF20220106, living culture CFCC 55551). Fengtai District, West Bureau Yupu Garden, 116°17′32″ E, 39°52′10″ N, from branches of *Euonymus japonicus*, H. Gao and X.L. Fan, 23 April 2021 (BJFC CF20220107, living culture CFCC 55552 and 57541).

*Notes*: In this study, four isolates CFCC 55550–55552 and 57541 were grouped together with *Aplosporella prunicola* and *A. yalgorensis* in phylogenetic analyses with 98 ML bootstrap support value and 0.98 posterior probabilities. The four isolates in this study can be distinguished from *A. yalgorensis* based on ITS and *tef* loci (for 10–11/520 bp in ITS, 2–6/317 bp in *tef*). In ML tree, CFCC 55550–55551 clustered with *A. prunicola* with 83 ML bootstrap support value with 100% repetitive ITS sequences. CFCC 55552 and 57541 are only one base different from *A. prunicola* in ITS region. Additionally, the conidia size of CFCC 55552 on PDA are 16.5–21.5 × 10.0–10.5 µm, which is consistent with the morphological characteristics of ex-type of *A. prunicola* for (17) 19–22 (25) × (9) 10–12 (18) [70]. Therefore, these four isolates in this study are identified as *Aplosporella prunicola*.

***Botryosphaeria*** Ces. and De Not., Comment. Soc. Crittog. Ital. 1: 211. 1863.

***Botryosphaeria dothidea*** (Moug.: Fr.) Ces. and De Not., Comment. Soc. Crittog. Ital. 1: 212. 1863.

*Basionym*: *Sphaeria dothidea* Moug., In: Fries, Syst. Mycol. (Lundae) 2 (2): 423. 1823

*Description*: See Phillips et al. [11].

*Material examined*: China. Beijing City, Chaoyang District, Olympic Forest Park, 116°23′9″ E, 40°0′2″ N, from branches of *Euonymus japonicus*, H. Gao and X.L. Fan, 26 April 2021 (BJFC CF20220110, living culture CFCC 55492–55496); 116°23′10″ E, 40°0′7″ N, from branches of *Euonymus japonicus*, H. Gao and X.L. Fan, 26 April 2021 (BJFC CF20220115, living culture CFCC 55569–55572); 116°23′10″ E, 40°0′2″ N, from branches of *Euonymus japonicus*, H. Gao and X.L. Fan, 7 May 2021 (BJFC CF20220119, living culture CFCC 55681). Haidian District, Beijing Forestry University, 116°20′28″ E, 40°0′8″ N, from branches of *Euonymus japonicus*, H. Gao and X.L. Fan, 23 April 2021 (BJFC CF20220108, living culture CFCC 55490; BJFC CF20220109, living culture CFCC 55491). Shijingshan District, Sculpture Garden Middle Street, 116°14′40″ E, 39°54′23″ N, from branches of *Euonymus japonicus*, H. Gao and X.L. Fan, 23 May 2021 (BJFC CF20220111, living culture CFCC 55564; BJFC CF20220117, living culture CFCC 55576–55577); 116°14′35″ E, 39°54′23″ N, from branches of *Euonymus japonicus*, H. Gao and X.L. Fan, 23 May 2021 (BJFC CF20220112, living culture CFCC 55565). Daxing District, Daxing New Town Riverfront Forest Park, 116°17′41″ E, 39°44′31″ N, from branches of *Euonymus japonicus*, H. Gao and X.L. Fan, 23 May 2021 (BJFC CF20220113, living culture CFCC 55566); 116°17′51″ E, 39°44′31″ N, from branches of *Euonymus japonicus*, H. Gao and X.L. Fan, 23 May 2021 (BJFC CF20220114, living culture CFCC 55567–55568). Mentougou District, Pushan Park, 116°6′35″ E, 39°55′30″ N, from diseased leaves of *Euonymus japonicus*, H. Gao and X.L. Fan, 14 May 2021 (BJFC CF20220116, living culture CFCC 55575). Xicheng District, Houhai Park, 116°22′48″ E, 39°56′21″ N, from twigs of *Euonymus japonicus*, H. Gao and X.L. Fan, 7 May 2021 (BJFC CF20220118, living culture CFCC 55578).

*Notes*: Cesati and De Notaris [71] first introduced the genus *Botryosphaeria* with 12 species described. However, they did not specify a type species of this genus. Barr et al. [72] suggested that *Botryosphaeria dothidea* (Basionym: *Sphaeria dothidea* Moug.: Fries [73]) should be a lectotype of this genus. Slippers et al. [74] re-examined that the host of the holotype of *Sphaeria dothidea* in the Fries herbarium was *Rosa* sp., which was not consistent with the description of Fries [73] (on *Fraxinus* sp.). Additionally, the only other specimen identified as *S. dothidea* on *Fraxinus* sp. in the Fries herbarium was immature with no spores [74,75]. This specimen was designated as a neotype [74]. Then, Slippers et al. [74] re-collected specimens from a nearby locality and designated an epitype (PREM 57372) on *Prunus* sp. collected from Crocifisso, Switzerland, with an ex-epitype culture (CBS 115476 = CMW 8000) with phylogenetic data. Zhang et al. [6] reduced four species to synonymy with *Botryosphaeria dothidea* based on the high sequence similarity values in ITS region. In this study, twenty-one isolates clustered together with *B. dothidea* in ML and BI trees. Therefore, they are identified as *Botryosphaeria dothidea*.

***Dothiorella*** Sacc., Michelia 2 (6): 5, 1880/

***Dothiorella acericola*** Phookamsak, Tennakoon and K.D. Hyde, Fungal Divers. 95: 78, 2019/

*Descriptions*: See Phookamsak et al. [76].

*Material examined*: China. Beijing City, Chaoyang District, Sun Palace South Street, 116°26′41″ E, 39°58′29″ N, from branches of *Euonymus japonicus*, H. Gao and X.L. Fan, 21 April 2021 (BJFC CF20220164, living culture CFCC 55559). Haidian District, Beijing Forestry University, 116°20′31″ E, 40°0′16″ N, from branches of *Euonymus japonicus*, H. Gao and X.L. Fan, 21 April 2021 (BJFC CF20220165, living culture CFCC 55561). Mentougou District, Shitan Road, 116°6′31″ E, 39°55′36″ N, from branches of *Euonymus japonicus*, H. Gao and X.L. Fan, 21 April 2021 (BJFC CF20220162, living culture CFCC 55556; BJFC CF20220163, living culture CFCC 55558).

*Notes*: *Dothiorella acericola* was first discovered on dead hanging twigs of *Acer palmatum* Thunb. in Yunnan Province, China [76]. In this study, four isolates (CFCC 55556–55558, and 55561) were grouped together with the ex-type of *Do. acericola*, KUMCC 18-0137, with a high statistical support (ML/BI = 90/1). Therefore, they are identified as *Dothiorella acericola*.

**Sodariomycetes** O.E. Erikss. and Winka, Myconet 1 (1): 10, 1997.

**Glomerellales** Chadef. ex Réblová, W. Gams and Seifert, Stud. Mycol. 68: 170, 2011.

***Colletotrichum*** Corda, in Sturm, Deutschl. Fl., 3 Abt. (Pilze Deutschl.) 3 (12): 41, 1831.

***Colletotrichum aenigma*** B.S. Weir and P.R. Johnst., in Weir, Johnston, and Damm, Stud. Mycol. 73: 135, 2012.

*Descriptions*: See Weir et al. [77].

*Material examined*: China. Beijing City, Daxing District, Daxing New Town Riverfront Forest Park, 116°17′40″ E, 39°44′31″ N, from leaf spots of *Euonymus japonicus*, H. Gao and X.L. Fan, 21 April 2021 (BJFC CF20220154, living culture CFCC 55535).

*Notes*: *Colletotrichum aenigma* was first discovered on *Persea americana* [77]. Over 20 host species of *Co. aenigma* were recorded (https://nt.ars-grin.gov/fungaldatabases/index.cfm, accessed on 16 January 2023). In this study, one isolate CFCC 55535 clustered into a clade with *Co. aenigma* ICMP 18608 with high statistical support (ML/BI = 93/0.99). Therefore, CFCC 55535 is identified as *Colletotrichum aenigma*. The host range of *Co. aenigma* also expanded to include *Euonymus japonicus*.

**Figure 6:**
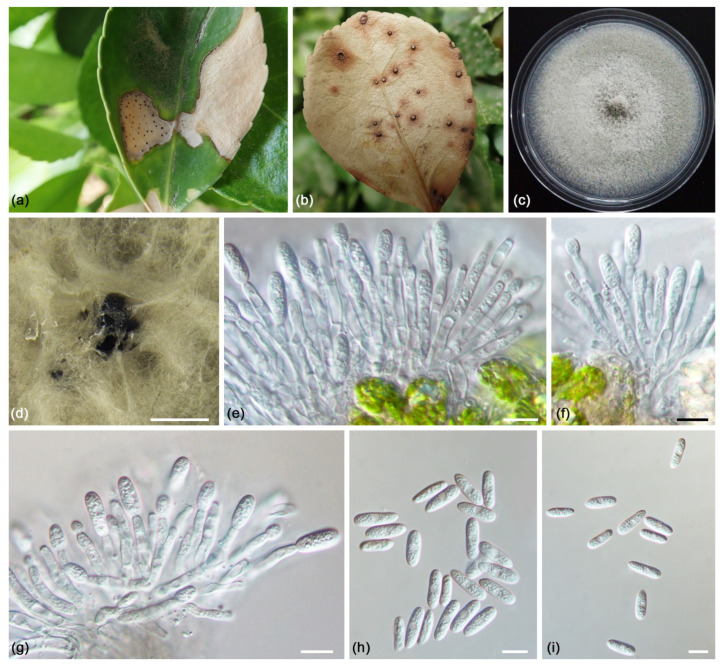
*Colletotrichum euonymi* (ex-holotype culture CFCC 55542): (**a**,**b**) Symptoms caused by *Colletotrichum euonymi*; (**c**) Front colony on PDA (10 d); (**d**) Conidiama on PDA (30 d); (**e**–**g**) Conidiophores and conidiogenous cells formed on inoculated *Euonymus japonicus*; (**h**,**i**) Conidia formed on inoculated *Euonymus japonicus*. Scale bars = 10 μm.

***Colletotrichum euonymi*** L. Lin and X.L. Fan sp. nov. (Figure 6).

*MycoBank*: MB 846880

*Etymology*: The name reflects the host genus from it was collected, *Euonymus*.

*Description*: *Colonies* on PDA reaching 75–80 mm diam after 7 d, flat, white to smoke grey, reverse honey in the center, and white towards the margin. *Conidiomata* 541–1193 µm, formed on the surface of PDA, covered by olivaceous buff mycelium. *Conidiophores* formed on inoculated *Euonymus japonicus*, hyaline, septate, unbranched, 24.5–47.5 × 2.5–4.0 µm (av. = 36.15 ± 5.17 × 3.46 ± 0.41 µm, n = 30). *Conidiogenous cells* hyaline, smooth-walled, cylindrical to subcylindrical, variable in size, 8.0–18.0 × 2.5–4.0 µm (av. = 13.46 ± 2.66 × 3.28 ± 0.32 µm, n = 30). *Conidia* hyaline, aseptate, smooth-walled, cylindrical to subcylindrical, guttulate, straight, 14.0–22.5 × 4.5–6.0 µm (av. = 16.67 ± 1.83 × 5.35 ± 0.42 µm, n = 50), L/W ratio = 3.13. *Appressoria* and *Setae* not observed.

*Typus*: China. Beijing City, Shijingshan District, Sculpture Garden Middle Street, 116°14′14″ E, 39°54′18″ N, from leaf spot of *Euonymus japonicus*, H. Gao and X.L. Fan, 7 May 2021 (**holotype** BJFC CF20220153, **ex-holotype** culture CFCC 55542). Haidian District, Beijing Forestry University, 116°20′28″ E, 40°0′8″ N, from leaf spot of *Euonymus japonicus*, H. Gao and X.L. Fan, 21 May 2021 (**paratype** BJFC CF20220152, **ex-paratype** culture CFCC 55483).

*Additional materials examined*: China. Beijing City, Chaoyang District, Olympic Forest Park, 116°23′13″ E, 40°0′4″ N, from leaf spot of *Euonymus japonicus*, H. Gao and X.L. Fan, 21 May 2021 (BJFC CF20220150, living culture CFCC 55540). Fengtai District, Lotus Pond Park, 116°18′17″ E, 39°53″ N, from leaf spot of *Euonymus japonicus*, H. Gao and X.L. Fan, 21 May 2021 (BJFC CF20220151, living culture CFCC 55537).

*Notes*: In this study, four isolates of *Colletotrichum euonymi* formed a distinct clade with high statistical support (ML/BI = 100/1). In *Colletotrichum gloeosporioides* complex, *Co. gloeosporioides* and *Co. siamense* have been recorded to host *Euonymus* [78,79]. However, *Co. euonymi* can be distinguished from them by larger conidia (14.0–22.5 × 4.5–6.0 µm vs. 12.0–17 × 4.5–6.0 µm for *Co. gloeosporioides* and 7–18.3 × 3–4.3 µm for *Co. siamense*) [78,79].

**Figure 7:**
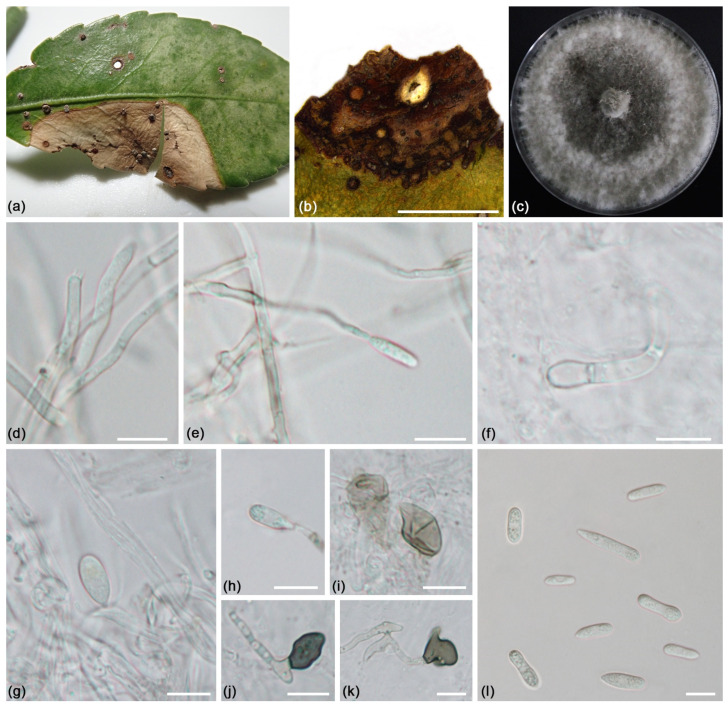
*Colletotrichum euonymicola* (ex-holotype culture CFCC 55486): (**a**,**b**) Symptoms caused by *Colletotrichum euonymicola*; (**c**) Front colony on PDA (10 d); (**d**–**h**) Conidiogenous cells formed on PDA; (**i**–**k**) Appiospora formed on PDA; (**l**) Conidia formed on PDA. Scale bars = 10 μm.

***Colletotrichum euonymicola*** L. Lin and X.L. Fan sp. nov. (Figure 7).

*MycoBank*: MB 846881

*Etymology*: The name reflects that the species is a *Euonymus*-colonizer.

*Description*: Colonies on PDA 73–82 mm diam in 7 d, flat with undulate edge, mouse grey to dark mouse grey, aerial mycelium short, *Conidiomata* not developed. *Conidiophores* formed directly from hyphae. *Conidiophores* hyaline to brown, septate, branched. *Conidiogenous cells* hyaline, smooth-walled, cylindrical or slightly tapering towards the apex, 10.5–16.5 × 1.0–3.5 μm. *Conidia* hyaline, aseptate, smooth-walled, guttulate, cylindrical with obtuse ends, with the base sometimes tapering to a truncate hilum, (9.5) 11.0–23.0 (25.0) × 3.5–6.5 (6.9) µm (av. = 14.3 ± 2.85 × 4.73 ± 0.69 µm, n = 50), L/W ratio = 3.04. *Appressoria* single, smoke grey to iron grey, terminally at the tip of the hyphae, irregularly shaped, with undulate to lobate margins, 10.0–14.5 × 6.0–8.0 μm. *Setae* was not observed.

*Typus*: China. Beijing City, Chaoyang District, 116°23′13″ E, 40°0′4″ N, Sun Palace Middle Street, from leaf spot of *Euonymus japonicus*, H. Gao and X.L. Fan, 7 May 2021 (**holotype** BJFC CF20220157, **ex-holotype** culture CFCC 55486; **paratype** BJFC CF20220158, **ex-paratype** culture CFCC 55539).

*Notes*: *Colletotrichum euonymicola* is phylogenetically related to *Co. pseudotheobromicola*. However, they show differences of sequence at ITS (13/549), *act* (4/284), *tub2* (1/445), *chs-1* (1/296), and *gapdh* (2/278). Morphologically, *Co. euonymicola* differs from *Co. pseudotheobromicola* in that it produces larger-sized appiospora (10.0–14.5 × 6.0–8.0 μm vs. 6–10 × 5–8 μm) [80]. Moreover, *Co. euonymicola* is different from *Co. pseudotheobromicola* in terms of host plant species (*Euonymus japonicus* v.s. *Prunus avium*). Therefore, *Colletotrichum euonymicola* is introduced as a novel species here.

***Colletotrichum gloeosporioides*** (Penz.) Penz. and Sacc., Atti Inst. Veneto Sci. lett., ed Arti, Sér. 6 2 (5): 670, 1884.

*Descriptions*: See Cannon et al. [78].

*Materials examined*: China. Beijing City, Xicheng District, Houhai Park, 116°22′34″ E, 39°56′25″ N, from branches of *Euonymus japonicus*, H. Gao and X.L. Fan, 7 May 2021 (BJFC CF20220155 living culture CFCC 55544–55545); 116°23′7″ E, 39°56′27″ N, from branches of *Euonymus japonicus*, H. Gao and X.L. Fan, 7 May 2021 (BJFC CF20220156 living culture CFCC 55547).

*Notes*: The host range of *Colletotrichum gloeosporioides* includes multiple families. It has been reported that *Co. gloeosporioides* can affected *Euonymus fortunei* and *Euonymus japonicus* [81,82]. In this study, three isolates grouped together with *Co. gloeosporioides* IMI 356878 with a high statistical support (ML/BI = 95/1). Therefore, they are identified as *Colletotrichum gloeosporioides* based on the phylogenetic tree.

**Diaporthales** Nannf., Nova Acta R. Soc. Scient. Upsal., Ser. 48 (2): 53, 1932.

***Cytospora*** Ehrenb., Sylv. mycol. berol. (Berlin): 28, 1818.

***Cytospora ailanthicola*** X.L. Fan and C.M. Tian, Persoonia 45: 13, 2020.

*Descriptions*: See Fan et al. [32].

*Material examined*: China. Beijing City, Chaoyang District, Olympic Forest Park, 116°23′9″ E, 40°0′2″ N, from branches of *Euonymus japonicus*, X.W. Zhu, 28 April 2020 (BJFC CF20220120, living culture CFCC 55529).

*Notes*: *Cytospora ailanthicola* was first introduced on branches of *Ailanthus altissima* [32]. Lin et al. [52] confirmed this species was a pathogen with strong virulence caused by poplar canker disease. In this study, one isolate, CFCC 55529, was isolated from symptomatic branches of *Euonymus japonicus* in Beijing, which clustered in a well-supported clade with *C. ailanthicola* ex-holotype CFCC 89970 (ML/BI = 100/1). Therefore, CFCC 55529 is identified as *Cytospora ailanthicola*.

***Cytospora albodisca*** M. Pan and X.L. Fan, Front. Plant Sci. 12 (636460): 3, 2021.

*Descriptions*: See Pan et al. [83].

*Material examined*: China. Beijing City, Mengtougou District, Baihuashan Nature Reserve, 115°33′15″ E, 39°51′52″ N, from branches of *Euonymus japonicus*, M. Pan and X.L. Fan, 21 August 2021 (BJFC CF20220121, living culture CFCC 56274, 57538).

*Notes*: *Cytospora albodisca* was first discovered on *Platycladus orientalis*, whose ascostroma was surrounded by a black conceptacle [83]. In this study, two isolates (CFCC 56274 and 57538) converged into a separate little branch. However, they only differ from *C. albodisca* CFCC 53161 and 54373 by 1/778 in *act* gene and 1/732 in *tub2* gene (with gaps). Additionally, the isolates in this study (CFCC 56274 and 57538) grouped together with *C. albodisca* CFCC 53161 and 54373 with a high statistical support (ML/BI = 94/1). Therefore, these two isolates are identified as *Cytospora albodisca*.

***Cytospora discostoma*** M. Pan and X.L. Fan, Front. Plant Sci. 12 (636460): 3, 2021.

*Descriptions*: See Pan et al. [83].

*Material examined*: China. Beijing City, Mengtougou District, Baihuashan Nature Reserve, 115°33′17″ E, 39°52′52″ N, from branches of *Euonymus japonicus*, M. Pan and X.L. Fan, 21 August 2021 (BJFC CF20220122, living culture CFCC 56276).

*Notes*: *Cytospora discostoma* was first discovered on branches of *Platycladus orientalis* at Mentougou District in Beijing [83]. In this study, one isolate, CFCC 56276, clustered in a well-support clade (ML/BI = 100/1) with *C. discostoma* CFCC 53137 and 54368. The specimen BJFC CF20220122 in this study was collected from branches of *Euonymus japonicus* at Mentougou District in Beijing, where *Cytospora discostoma* was first discovered.

***Cytospora diopuiensis*** Q.J. Shang, J.K. Liu and K.D. Hyde, Mycosphere, 11 (1): 202, 2020.

*Descriptions*: See Shang et al. [84].

*Material examined*: China. Beijing City, Haidian District, Beijing Forestry University, 116°20′28″ E, 40°0′8″ N, from leaves of *Euonymus japonicus*, H. Gao and X.W. Zhu, 21 October 2020 (BJFC CF20220146, living culture CFCC 54692; BJFC CF20220147, living culture CFCC 55479). Shijingshan District, Sculpture Garden Middle Street, 116°14′11″ E, 39°54′10″ N, from branches of *Euonymus japonicus*, H. Gao and X.L. Fan, 20 April 2021 (BJFC CF20220148, living culture CFCC 55527; BJFC CF20220149, living culture CFCC 55528).

*Notes*: *Cytospora diopuiensis* was discovered on bark of dead wood in Thailand [84]. Jiang et al. [85] reported this species on *Kerria japonica* f. *pleniflora* in China. In this study, two isolates from leaves of *Euonymus japonicus* and two isolates from branches clustered in a well-supported clade with *C. diopuiensis* (ML/BI = 100/1). Therefore, they were identified as *Cytospora diopuiensis*.

***Cytospora elaeagni*** Allesch., Hedwigia 36: 162, 1897.

*Descriptions*: See Fan et al. [86].

*Material examined*: China. Beijing City, Shijingshan District, Beijing International Sculpture Park, 116°14′11″ E, 39°54′10″ N, from branches of *Euonymus japonicus*, H. Gao and X.L. Fan, 23 April 2021 (BJFC CF20220123, living culture CFCC 54082). Haidian District, Beijing Forestry University, 116°20′27″ E, 40°0′13″ N, from leaves of *Euonymus japonicus*, H. Gao and X.L. Fan, 21 April 2021 (BJFC CF20220124, living culture CFCC 55477). The Shucun Park, 116°17′55″ E, 40°0′53″ N, from branches of *Euonymus japonicus*, H. Gao and X.L. Fan, 21 April 2021 (BJFC CF20220125, living culture CFCC 55526). Mengtougou District, Baihuashan Nature Reserve, 115°33′25″ E, 39°51′53″ N, from branches of *Euonymus japonicus*, M. Pan, Y.K. Bai and X.L. Fan, 21 August 2021 (BJFC CF20220126, living culture CFCC 56273). 115°34′15″ E, 39°51′56″ N, from branches of *Euonymus japonicus*, M. Pan, Y.K. Bai and X.L. Fan, 21 August 2021 (BJFC CF20220127, living culture CFCC 56287).

*Notes*: *Cytospora elaeagni* has been reported from *Elaeagnus angustifolia* in China, German, and the USA [87,88,89]. Fan et al. [86] provided its morphological descriptions and molecular data. In this study, five isolates are identified as *Cytospora elaeagni* based on phylogenetic analyses. A new host record from *Euonymus japonicus* is provided here.

***Cytospora euonymicola*** X.L. Fan and C.M. Tian, Persoonia 45: 13, 2020.

*Descriptions*: See Fan et al. [32].

*Material examined*: China. Beijing City, Haidian District, Beijing Forestry University, 116°20′31″ E, 40°0′16″ N, from branches of *Euonymus japonicus*, H. Gao and X.L. Fan, 21 April 2021 (BJFC CF20220128, living culture CFCC 54688). Fengtai District, Lotus Pond Park, 116°18′17″ E, 39°53′34″ N, from branches of *Euonymus japonicus*, H. Gao and X.L. Fan, 21 April 2021 (BJFC CF20220129, living culture CFCC 55530).

*Notes*: *Cytospora euonymicola* was first introduced on *Euonymus kiautschovicus* in Shaanxi Province, China [32]. In this study, two isolates grouped together with *C. euonymicola* in ML and BI trees (ML/BI = 100/1). Morphologically, the conidia size in this study were similar in *C. euonymicola* described by Fan et al. [32] (4.5–5.0 × 1.0–1.5 μm vs. 4–5 × 1 μm). Therefore, the two isolates in the current study are identified as *Cytospora euonymicola* based on phylogeny and morphology.

***Cytospora euonymina*** X.L. Fan and C.M. Tian, Persoonia 45: 13, 2020.

*Descriptions*: See Fan et al. [32].

*Material examined*: China. Beijing City, Daxing District, Nanchengzhuang, 116°17′39″ E, 39°44′26″ N, from branches of *Euonymus japonicus*, H. Gao and X.L. Fan, 11 April 2021 (BJFC CF20220130, living culture CFCC 55524). Xicheng District, Houhai Park, 116°22′49″ E, 39°56′21″ N, from branches of *Euonymus japonicus*, H. Gao and X.L. Fan, 21 April 2021 (BJFC CF20220131, living culture CFCC 55525).

*Notes*: *Cytospora euonymina* was first introduced on *Euonymus kiautschovicus* in Shanxi Province, China [32]. In this study, two isolates grouped together with *C. euonymina* in ML and BI trees (ML/BI = 100/1). Therefore, they were identified as *C. euonymina*. Additionally, CFCC 55524 and CFCC 55525 were isolated from leaves of *Euonymus japonicus* in the current study. The discs of conidioma formed on leaves (100–150 µm in this study) were smaller than the description of Fan et al. (2020) (200–230 µm, on branches).

***Cytospora haidianensis*** X. Zhou and X.L. Fan, Forests, 11 (5): 524, 2020.

*Descriptions*: See Zhou et al. [33].

*Material examined*: China. Beijing City, Chaoyang District, Olympic Forest Park, 116°23′9″ E, 40°0′2″ N, from branches of *Euonymus japonicus*, H. Gao and X.L. Fan, 21 April 2021 (BJFC CF20220132, living culture CFCC 55480; BJFC CF20220134, living culture CFCC 55532). 116°23′10″ E, 40°0′2″ N, from branches of *Euonymus japonicus*, H. Gao and X.L. Fan, 21 April 2021 (BJFC CF20220135, living culture CFCC 55533). Shijingshan District, Sculpture Garden Middle Street, 116°14′14″ E, 39°54′18″ N, from branches of *Euonymus japonicus*, H. Gao and X.L. Fan, 21 April 2021 (BJFC CF20220133, living culture CFCC 55531).

*Notes*: *Cytospora haidianensis* was first introduced as a pathogen on *Euonymus alatus* by Zhou et al. [33]. This species has a toruloid locule with a central column of ostiolar tissue [33]. In this study, four isolates grouped together with *C. haidianensis* in ML and BI trees (ML/BI = 100/1). Therefore, they are identified as *Cytospora haidianensis*.

***Cytospora leucostoma*** (Pers.) Sacc., Michelia, 2 (7): 264, 1881.

*Descriptions*: See Fan et al. [32].

*Material examined*: China. Beijing City, Haidian District, Beijing Forestry University, 116°20′27″ E, 40°0′13″ N, from branches of *Euonymus japonicus*, H. Gao and X.L. Fan, 21 April 2021 (BJFC CF20220136, living culture CFCC 55474). 116°20′32″ E, 40°0′13″ N, from branches of *Euonymus japonicus*, H. Gao and X.L. Fan, 21 April 2021 (BJFC CF20220137, living culture CFCC 55475). 116°20′31″ E, 40°0′16″ N, from branches of *Euonymus japonicus*, H. Gao and X.L. Fan, 21 April 2021 (BJFC CF20220138, living culture CFCC 55476). 116°20′28″ E, 40°0′17″ N, from branches of *Euonymus japonicus*, H. Gao and X.L. Fan, 21 April 2021 (BJFC CF20220139, living culture CFCC 55478). Chaoyang District, Olympic Forest Park, 116°23′10″ E, 40°0′2″ N, from branches of *Euonymus japonicus*, H. Gao and X.L. Fan, 21 April 2021 (BJFC CF20220140, living culture CFCC 55519; BJFC CF20220142, living culture CFCC 55521). Mentougou District, The Shitan Road, 116°6′31″ E, 39°55′36″ N, from branches of *Euonymus japonicus*, H. Gao and X.L. Fan, 21 April 2021 (BJFC CF20220141, living culture CFCC 55520).

*Notes*: Fan et al. [32] revealed *C. donetzica* as synonym of *C. leucostoma* based on DNA data and descriptions. In this study, seven isolates grouped together with *C. leucostoma* in ML and BI trees (ML/BI = 100/1). Morphologically, the size of conidia in this study was 5.0–6.0 × 1.0–2.0 µm, which overlapped with the description by Fan et al. [32] (4.5–5.5 × 1–1.5 μm). Therefore, these seven isolates are identified as *Cytospora leucostoma*.

***Cytospora sophorae*** Bres., Fung. trident. 2 (8–10): 44, 1892.

*Descriptions*: See Fan et al. [90].

*Material examined*: China. Beijing City, Mentougou District, Lisichen Park, 116°6′31″ E, 39°55′37″ N, from leaves spots of *Euonymus japonicus*, H. Gao and X.L. Fan, 11 May 2021 (BJFC CF20220143, living culture CFCC 55523).

*Notes*: *Cytospora sophorae* has been recorded as a pathogen caused *Sophora* canker disease in China [89,91]. Fan et al. [90] provided the description and DNA data of this species. In this study, one isolate CFCC 55523 grouped together with *C. sophorae* in ML and BI trees (ML/BI = 100/1). Therefore, it is identified as *Cytospora sophorae*.

**Figure 8:**
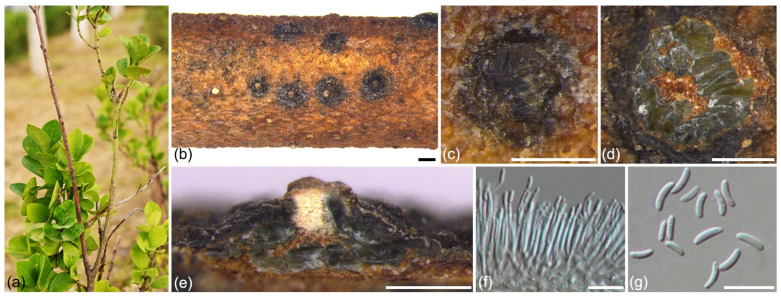
*Cytospora zhaitangensis* (holotype BJFC CF20220144): (**a**) Diseased Euonymus japonicus branches caused by *Cytospora zhaitangensis* in the field; (**b**,**c**) Habit of conidiomata on twig; (**d**) Transverse section of a conidioma; (**e**) Longitudinal section through a conidioma; (**f**) Conidiophores and conidiogenous cells; (**g**) Conidia. Scale bars: 500 µm (**b**–**e**), 10 µm (**f**,**g**).

***Cytospora zhaitangensis*** L. Lin and X.L. Fan sp. nov. (Figure 8).

*MycoBank*: MB 846882

*Etymology*: The name reflects the station where the holotype and paratype specimens were collected, next to the Zhaitang reservoir.

*Description*: Asexual morph: Conidiomata cytosporoid rosette, immersed in bark, erumpent when mature, discoid to conical, 524–936 µm in diam, with multiple locules. Conceptacles were absent. Disc hazel to olivaceous, circular to ovoid, 166–224 µm in diam, with a single ostiole per disc in the center. Ostiole circular to ovoid, olivaceous buff to dark mouse grey, at the same level or above as disc surface, 66–98 µm in diam. Locules are complex with irregular shapes, and do not share common walls. Conidiophores hyaline, unbranched, or branched at the bases or at mid-height, 13.5–27.0 (30.0) × 1.0–2.0 µm (av. = 19.71 ± 4.51 × 1.40 ± 0.18 µm, n = 30). Conidiogenous cells enteroblastic, phialidic, subcylindrical to cylindrical, 6.0–11.0 × 1.0–2.0 µm (av. = 8.05 ± 1.29 × 1.37 ± 0.18 µm, n = 30). Conidia hyaline, unicellular, eguttulate, elongate-allantoid, (3.8) 4.0–6.5 × (1.2) 1.3–1.6 (1.7) µm (av. = 5.14 ± 0.74 × 1.47 ± 0.12 µm, n = 50), L/W ratio = 3.48. Sexual morph: not observed.

*Typus*: China. Beijing City, Mengtougou District, next to the Zhaitang Reservoir, 115°33′15″ E, 39°51′52″ N, from branches of *Euonymus japonicus*, H. Gao and X.L. Fan, 21 August 2021 (**holotype** BJFC CF20220144, **ex-holotype** culture CFCC 56227). 115°33′16″ E, 39°51′54″ N, from branches of *Euonymus japonicus*, M. Pan, Y.K. Bai and X.L. Fan, 21 August 2021 (**paratype** BJFC CF20220145, **ex-paratype** culture CFCC 57537).

*Notes*: *Cytospora zhaitangensis* is phylogenetically most closely related to *C. euonymicola* and *C. gigalocus*. Morphologically, *C. zhaitangensis* can be differentiated by the wider conidia from *C. euonymicola* (L/W ratio = 3.48 vs. L/W ratio = 4.5) and *C. gigalocus* (L/W ratio = 4.36) [32,86]. Additionally, *C. zhaitangensis* has smaller discs (166–224 µm) than *C. euonymicola* (240–350 µm) and *C. gigalocus* (330–620 µm) [32,86]. *Cytospora euonymi* and *C. euonymella* were also recorded to host *Euonymus* [92,93]. *Cytospora zhaitangensis* can be differentiated by the conidia size from *C. euonymella* (4.0–6.5 × 1.3–1.6 vs. 2.5 × 0.5 µm) and *C. euonymi* (8 × 2 µm) [92,93].

***Diaporthe*** Nitschke, Pyrenomyc. Germ. 2: 240, 1870.

***Diaporthe eres*** Nitschke, Pyrenomyc. Germ. 2: 240, 1870.

*Descriptions*: See Gao et al. [26].

*Material examined*: China. Beijing City, Chaoyang District, Olympic Forest Park, 116°23′9″ E, 40°0′2″ N, from branches of *Euonymus japonicus*, H. Gao and X.L. Fan, 21 April 2021 (BJFC CF20220159, living culture CFCC 55481). 116°26′52″ E, 39°58′5″ N, from branches of *Euonymus japonicus*, H. Gao and X.L. Fan, 21 April 2021 (BJFC CF20220160, living culture CFCC 55482). Shijingshan District, Sculpture Garden Middle Street, 116°14′14″ E, 39°54′18″ N, from branches of *Euonymus japonicus*, H. Gao and X.L. Fan, 21 April 2021 (BJFC CF20220161, living culture CFCC 55534).

*Notes*: Norphanphoun et al. [94] introduced 13 species complexes of *Diaporthe*, with revealing *D. eres* species complex introduced by Udayanga et al. [95] as *D. alnea* species complex according to nomenclatural articles. *Diaporthe eres* strains collected from different hosts were dispersed in the clade [94,95]. In this study, three isolates were dispersed in *Diaporthe alnea* species complex. To avoid over-classification, we only identified them as *Diaporthe eres*.

***Amphisphaeriales*** D. Hawksw. and O.E. Erikss., Syst. Ascom. 5 (1): 177, 1986.

***Pestalotiopsis*** Steyaert, Bull. Jard. Bot. État Brux. 19: 300, 1949.

**Figure 9:**
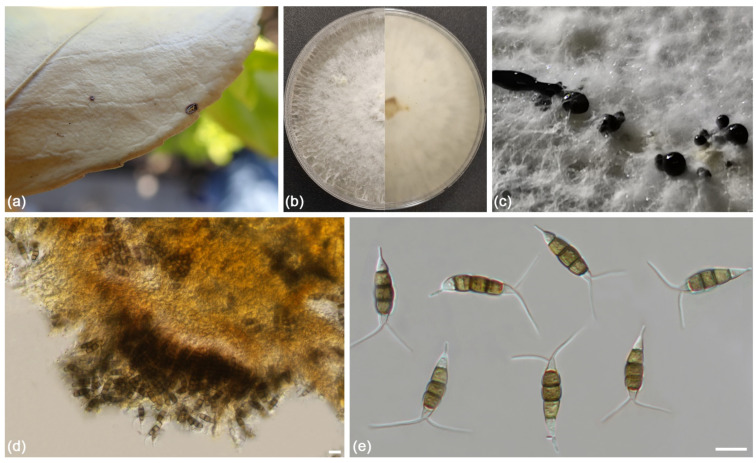
*Pestalotiopsis chaoyangensis* (ex-holotype culture CFCC 55549): (**a**) Diseased *Euonymus japonicus* leaves caused by *Pestalotiopsis chaoyangensis*; (**b**) Colony on PDA at 30 days; (**c**) Conidial masses formed on PDA; (**d**) Conidiogenous cells; (**e**) Conidia. Scale bars: 10 µm.

***Pestalotiopsis chaoyangensis*** L. Lin and X.L. Fan sp. Nov. (Figure 9).

*MycoBank*: MB 846883

*Etymology*: The name reflects the station where the holotype and paratype specimens were collected, Chaoyang District, Beijing, China.

*Description*: On PDA, *conidiomata* was not observed. *Conidial masses* abundant, black, scattered, or confluent, formed among the mycelia. Conidiophores are indistinct, and are usually reduced to conidiogenous cells. Conidiogenous cells hyaline, smooth, cylindrical to subcylindrical. Conidia fusoid, straight or slightly curved, 4-septate, smooth, slightly constricted at the septa, 19.5–25.5 × 4.5–6.5 µm (av. = 22.34 ± 1.17 × 5.52 ± 0.38 µm, n = 50), L/W ratio = 4.05; basal cell obconic with a truncate base, thin-walled, hyaline or pale brown, 3.5–5.5 µm (av. = 4.52 ± 0.44 µm, n = 50); median cells 3, trapezoid or subcylindrical, concolorous, pale brown to brown, thick-walled, the first median cell from base 3.5–6.0 µm (av. = 4.36 ± 0.52 µm, n = 50) mm long, the second cell 4.0–6.5 µm (av. = 4.88 ± 0.39 µm, n = 50) long, the third cell 3.5–5.5 µm (av. = 4.73 ± 0.43 µm, n = 50) long; apical cell conic with an acute apex, thin-walled, hyaline, 2.0–4.5 µm (av. = 3.44 ± 0.43 µm, n = 50) long; basal appendage single, occasionally 2, tubular, centric, straight or slightly bent, 2.0–6.5 µm (av. = 4.02 ± 1.14 µm, n = 50) long; apical appendages 2, unbranched, tubular, centric, straight or bent, 14.0–22.5 µm (av. = 17.56 ± 2.38 µm, n = 50). Sexual morph is unknown.

*Typus*: China. Beijing City, Chaoyang District, Olympic Forest Park, 116°23′10″ E, 40°0′2″ N, from leaves spots of *Euonymus japonicus*, H. Gao and X.L. Fan, 21 April 2021 (**holotype** BJFC CF20220167, **ex-holotype** culture CFCC 55549; **paratype** BJFC CF20220168, **ex-paratype** culture CFCC 58805).

*Notes*: Two isolates of *Pestalotiopsis chaoyangensis* (CFCC 55549 and 58805) formed a distinct clade phylogenetically close to *P. shaanxiensis* (Figure 5). However, *P. chaoyangensis* can be distinguished from *P. shaanxiensis* by the number of apical appendages with two for the former and three for the latter [39].

### 3.4. Pathogenicity Test

**Figure 10:**
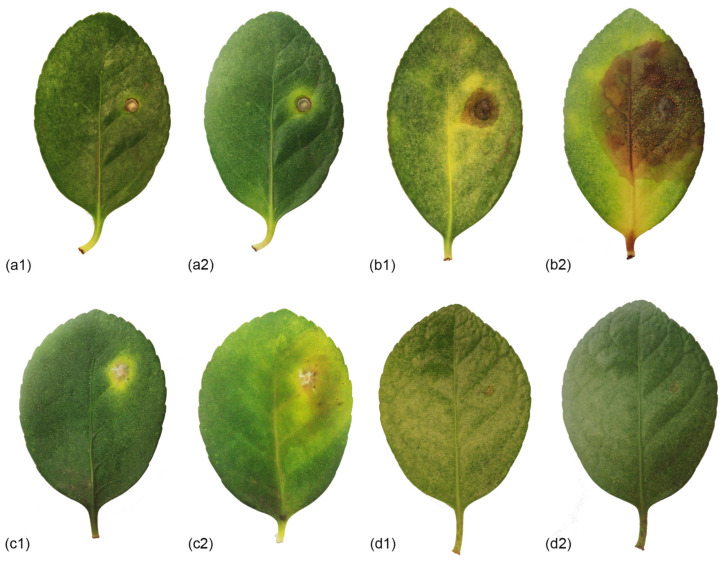
Lesions (1, 14 d; 2, 17d) resulting from inoculation of three novel species isolated from leaves onto *Euonymus japonicus*. Disease symptoms inoculated with (**a**) *Colletotrichum euonymi* (CFCC 55542); (**b**) *Colletotrichum euonymicola* (CFCC 55486); (**c**) *Pestalotiopsis chaoyangensis* (CFCC 55549); (**d**) Blank control.

**Figure 11:**
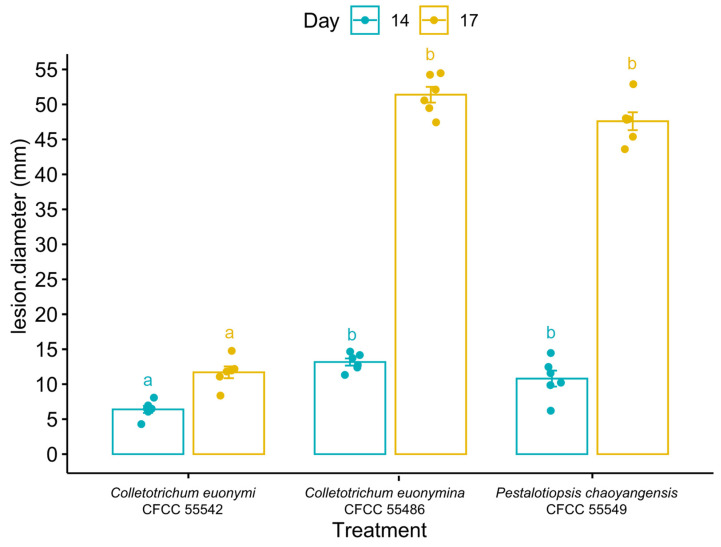
Average lesion diameter (mm) resulting from inoculation with *Euonymus japonicus*. Vertical bars represent standard error of means. Different letters above the bars indicate treatments that were significantly different (*α* = 0.05).

In the leaf inoculation assays, fourteen days after inoculation, leaf lesions were caused by all three species isolated from leaves (*Co. euonymi* CFCC 55542, *Co. euonymicola* CFCC 55486, and *P. chaoyangensis* CFCC 55549) (Figure 10 and Figure 11, Table 4). Disease sites initially turned yellow, and as disease progressed, diseased patches enlarged and took on a water-stained appearance, ultimately leading to wilting and consequent death. No symptoms were observed in the non-inoculated controls. All pathogenic species were re-isolated from lesions or conidia masses of inoculated leaves.

In the branch inoculation assay, no symptoms were observed with *C. zhaitangensis* inoculation or in the non-inoculated controls.

## 4. Discussion

*Euonymus japonicus* is an evergreen shrub that often becomes seriously diseased and even dies from fungal infestations in Beijing, China. In the current study, 79 isolates were obtained from 104 specimens collected from seven districts in Beijing City. The isolates included 22 species in seven genera, which were *Aplosporella* (eight isolates, three species), *Botryosphaeria* (21 isolates, one species), *Colletotrichum* (10 isolates, four species), *Cytospora* (31 isolates, 11 species), *Diaporthe* (three isolates, one species), *Dothiorella* (four isolates, one species), and *Pestalotiopsis* (two isolates, one species). Among the 22 species, *Co. euonymi*, *Co. euonymicola*, *C. zhaitangensis*, and *P. chaoyangensis* were identified as novel species on the basis of morphological and phylogenetic analyses. *Colletotrichum euonymi*, *Co. euonymicola*, and *P. chaoyangensis* were confirmed as pathogens on leaves of *E. japonicus*. In this study, *A. hesperidica*, *A. javeedii*, *A. prunicola*, *Co. aenigma*, *C. ailanthicola*, *C. albodisca*, *C. diopuiensis*, *C. discotoma*, *C. elaeagni*, *C. sophorae*, and *Do. acericola* were first recorded on the host genus *Euonymus*.

*Cytospora* had the highest diversity of species associated with *E. japonicus* (11 species). The genus includes numerous important pathogens and saprophytic fungi on various hosts [32,33,83,96]. Branch and stem diseases frequently result in skin rot, dryness, and plant death [51,52,57,97]. Among the 10 known species obtained from *E. japonicus* in the current study, *C. ailanthicola* and *C. haidianensis* are confirmed pathogens on *Populus* and *Euonymus*, respectively [32,52]. However, *C. zhaitangensis*, the novel species isolated from branches, was not pathogenic on *E. japonicus*. The pathogenicity of other *Cytospora* species on *Euonymus* needs to be studied further.

*Botryosphaeria dothidea* was the species with the highest number of isolates (21 isolates), which were distributed in Chaoyang, Daxing, Haidian, Mentougou, Shijingshan, and Xicheng districts. Chaoyang District had the most fungal species occurring on *E. japonicus* (11 species in seven genera), followed by Haidian and Mentougou districts (nine species in five and four genera, respectively). The differences among districts could be because Chaoyang District is a major industrial district in Beijing, which has more environmental pollution. Schmidt et al. [98] concluded that Sordariomycetous fungi dominated at the polluted site and species diversity of endophytes was higher at the unpolluted site. These changes could weaken plants and increase susceptibility to disease.

Multiple infections can occur on different plant parts. For example, *B. dothidea* causes apple ring rot of stems, twigs, and fruits [99], and *Co. gloeosporioides* causes anthracnose of leaves and fruits [100]. Among the seven genera in this study, *Aplosporella*, *Botryosphaeria*, *Cytospora*, *Dothiorella*, and *Diaporthe* are pathogenic and cause canker and dieback disease of various hosts [6,29,32,64,69,94,96]. However, eight isolates were obtained from leaves in the current study, i.e., *B. dothidea* CFCC 55575, *C. diopuiensis* CFCC 54692 and 55479, *C. elaeagni* CFCC 55477, *C. euonymina* CFCC 55524 and 55525, *C. sophorae* CFCC 55523, and *Do. acericola* CFCC 55559. The isolates also created reproductive structures on leaves, which indicated that the species may be able to infect branches as well as leaves. The pathogenicity of these species to leaves and stems needs to be studied further. Increased understanding of pathogen diversity is beneficial to *E. japonicus* production and maintenance because impacts of disease can be minimized, and disease management needs to be improved.

Over 90 species of fungi occurring on *E. japonicus* are recorded in the Fungal database (https://nt.ars-grin.gov/fungaldatabases/index.cfm; accessed on 16 January 2022). There are numerous cases of *Erysiphe* and *Phytophthora* infesting *Euonymus* in China, in addition to the taxa identified in the current study [88,89,101,102]. *Erysiphe alphitoides*, *E. euonymi*, *E. euonymicola*, *E. lianyungangensis*, *E. mayumi*, and *E. pseudopusilla* have been recorded on host *Euonymus* [102,103,104,105]. *Phytophthora citrophthora*, *P. meadii*, and *P. palmivora* have been reported on host *E. japonicus* [88,89,101]. Therefore, the two genera also need to be considered in disease control.

## Figures and Tables

**Table 1:** Genes used in this study with PCR primers and optimal annealing temperature.

Locus	PCR Primers	PCR: Thermal Cycles: (Annealing Temp. in Bold)	Reference	Genera
ITS	ITS1/ITS4	(95 °C: 30 s, **51 °C**: 30 s, 72 °C: 1 min) × 35 cycles	[45]	*Aplosporella*, *Botryosphaeria*, *Cytospora*, *Colletotrichum*, *Diaporthe*, *Dothiorella*, and *Pestalotiopsis*
*tef1-α*	EF1-728F/EF1-986R	(95 °C: 15 s, **55 °C**: 20 s, 72 °C: 1 min) × 35 cycles	[46]	*Aplosporella*, *Botryosphaeria*, *Cytospora*, *Diaporthe*, *Dothiorella*, and *Pestalotiopsis*
*tub2*	Bt2a/Bt2b	(95 °C: 30 s, **55 °C**: 30 s, 72 °C: 1 min) × 35 cycles	[47]	*Botryosphaeria*, *Cytospora*, *Colletotrichum*, *Diaporthe*, *Dothiorella*, and *Pestalotiopsis*
*act*	ACT-512F/ACT-783R	(95 °C: 45 s, **55 °C**: 45 s, 72 °C: 1 min) × 35 cycles	[46]	*Cytospora* and *Colletotrichum*
*rpb2*	RPB2-5F/RPB2-7cR	(95 °C: 30 s, **52 °C**: 1 min, 72 °C: 1 min) × 35 cycles	[46]	*Cytospora*
*chs1*	CHS-79F/CHS-345R	(95 °C: 30 s, **58 °C**: 30 s, 72 °C: 1 min) × 35 cycles	[46]	*Colletotrichum*
*gaphd*	GDF1/GDF2	(95 °C: 30 s, **58 °C**: 30 s, 72 °C: 1 min) × 35 cycles	[49]	*Colletotrichum*
*cal*	CAL228F/CAL737R	(95 °C: 15 s, **55 °C**: 20 s, 72 °C: 1 min) × 35 cycles	[46]	*Diaporthe*
*his3*	CYLH3F/H3-1b	(95 °C: 30 s, **58 °C**: 30 s, 72 °C: 1 min) × 35 cycles	[47,50]	*Diaporthe*

**Table 2:** Substitution models used for Bayesian analyses in this study.

Analysis	Number of Ingroup Sequences	Outgroup	Substitution Models Used for Bayesian Analyses/Number of Characters with Gaps								
			ITS	*tef1*	*tub2*	*act*	ITS	*chs*	*gaphd*	*cal*	ITS
*Aplosporella* 2-genes	27	*Alanomyces indica* CBS 134264	SYM + G/558	HKY + G/317	–	–	–	–	–	–	–
*Botryosphaeria* 3-genes	71	*Cophinforma eucalypti* CBS 134651	GTR + I/448	GTR + G/266	HKY + G/415	–	–	–	–	–	–
*Colletotrichum* 1-gene	487	*Monilochaetes infuscans* CBS 869.96	GTR + I + G/611	–	–	–	–	–	–	–	–
*Colletotrichum gloeosporioides* complex 5-genes	67	*Colletotrichum vietnamense* CBS 125477	GTR + I + G/555	–	HKY + G/451	HKY + G/290	–	K80 + I + G/275	HKY + G/278	–	–
		*Colletotrichum vietnamense* CBS 125478									
*Cytospora* 5-genes	277	*Diaporthe vaccinii* CBS 160.32	GTR + I + G/626	HKY + I + G/788	HKY + I + G/680	GTR + I + G/347	GTR + I + G/732	–	–	–	–
*Diaporthe* 5-genes	307	*Diaporthella corylina* CBS 121124	GTR + I + G/551	HKY + I + G/678	GTR + I + G/533	–	–	–	–	HKY + I + G/625	GTR + I + G/625
*Aplosporella* 2-genes	27	*Alanomyces indica* CBS 134264	SYM + G/558	HKY + G/317	–	–	–	–	–	–	–
*Botryosphaeria* 3-genes	71	*Cophinforma eucalypti* CBS 134651	GTR + I/448	GTR + G/266	HKY + G/415	–	–	–	–	–	–

**Table 3:** The statistics of ML trees in this study.

Analysis	Figure Number	Estimated Base Frequencies				Substitution Rates						Gamma Distribution Shape Parameter α A
		A	T	C	G	AC	A	T	C	CT	GT	
*Aplosporella*	Figure S1	0.212988	0.257488	0.274402	0.255122	1.887152	3.178340	1.573282	1.399408	2.980772	1.000000	0.382287
*Botryosphaeria*	Figure S2	0.215054	0.222293	0.312418	0.250235	0.441342	2.413944	0.639018	0.521572	3.599492	1.000000	0.240522
*Colletotrichum*	Figure S3	0.227417	0.235495	0.284682	0.252405	1.597190	2.099216	1.489264	1.500977	5.675789	1.000000	0.260939
*Colletotrichum gloeosporioides* complex	Figure 3	0.229013	0.228092	0.298051	0.244843	0.821304	2.465133	0.850090	0.790048	4.448447	1.000000	0.393050
*Cytospora*	Figure 4	0.244498	0.230131	0.286014	0.239357	1.288489	3.541841	1.487837	0.962637	6.197985	1.000000	0.360802
*Diaporthe*	Figure S4	0.213713	0.223461	0.326805	0.236021	1.074776	3.154257	1.134578	0.879474	4.526775	1.000000	0.509636
*Dothiorella*	Figure S5	0.204842	0.229767	0.320640	0.244750	0.762653	1.883624	1.132143	0.814926	3.755328	1.000000	0.228281
*Pestalotiopsis*	Figure 5	0.232209	0.258050	0.294948	0.214793	0.941298	2.870198	0.998145	0.973783	3.778432	1.000000	0.294632

**Table 4:** Lesions diameter (mm) of inoculated leaves.

Treatment	Days after Inoculation	Lesion Diameter (mm)						Average (mm)	Sd. (mm)
*Colletotrichum euonymi* CFCC 55542	14	6.5	7.0	8.1	4.3	6.5	6.1	6.4	1.24
	17	11.8	12.0	14.8	8.4	12.2	11.1	11.7	2.06
*Colletotrichum euonymicola* CFCC 55486	14	12.4	13.7	14.2	11.3	12.7	14.7	13.2	1.24
	17	49.5	52.1	54.2	47.4	50.6	54.5	51.4	2.76
*Pestalotiopsis chaoyangensis* CFCC 55549	14	9.9	10.2	14.5	6.2	12.5	11.6	10.8	2.79
	17	45.4	47.8	52.9	43.6	48.0	47.9	47.6	3.14
Blank control	14	4	4	4	4	4	4	4.0	0.0
	17	4	4	4	4	4	4	4.0	0.0

## Data Availability

Alignments generated during the current study are available in Tree-BASE (accession http://purl.org/phylo/treebase/phylows/study/TB2:S29991 (accessed on 15 February 2023)). All sequence data are available in NCBI GenBank following the accession numbers in the manuscript.

## Supplementary Materials

The following supporting information can be downloaded at: https://www.mdpi.com/article/10.3390/jof9020271/s1, Figure S1. Phylogram of *Aplosporella* based on maximum likelihood (ML) analysis of the dataset of combined ITS and *tef1-α* genes; Figure S2. Phylogram of *Botryosphaeria* based on maximum likelihood (ML) analysis of the dataset of combined ITS, *tef1-α*, and *tub2* genes; Figure S3. Phylogram of *Colletotrichum* based on maximum likelihood (ML) analysis of the dataset of ITS gene; Figure S4. Phylogram of *Diaporthe* based on maximum likelihood (ML) analysis of the dataset of combined ITS, *cal*, *his3*, *tef1-α*, and *tub2* genes; Figure S5. Phylogram of *Dothiorella* based on maximum likelihood (ML) analysis of the dataset of combined ITS, *tef1-α*, and *tub2* genes; Table S1: Strains used in the molecular analyses in this study.

## References

[1] Christenhusz M.J.M., Byng J.W. (2016). The number of known plants species in the world and its annual increase. Phytotaxa.

[2] Zhang T., Bai Y., Hong X., Sun L., Liu Y. (2017). Particulate matter and heavy metal deposition on the leaves of *Euonymus japonicus* during the East Asian monsoon in Beijing, China. PLoS ONE.

[3] Shi H.W., Yang L.F., Ding Z.Q., Tu J.H. (2010). Characteristics of absorption and enrichment of heavy metal in *Photinia serrulata* and *Euonymus japonicus* planted in sewage sludge substrates. North. Hortic..

[4] Raza M., Zhang Z.F., Hyde K.D., Diao Y.Z., Cai L. (2019). Culturable plant pathogenic fungi associated with sugarcane in southern China. Fungal Divers..

[5] Crous P.W., Wingfield M.J., Cheewangkoon R., Carnegie A.J., Burgess T.I., Summerell B.A., Edwards J., Taylor P.W.J., Groenewald J.Z. (2019). Foliar pathogens of eucalypts. Stud. Mycol..

[6] Zhang W., Groenewald J.Z., Lombard L., Schumacher R.K., Phillips A.J.L., Crous P.W. (2021). Evaluating species in Botryosphaeriales. Pers.-Mol. Phylogeny Evol. Fungi.

[7] Dissanayake A.J., Phillips A.J.L., Li X.H., Hyde K.D. (2016). Botryosphaeriaceae, Current status of genera and species. Mycosphere.

[8] Yang T., Groenewald J.Z., Cheewangkoon R., Jami F., Abdollahzadeh J., Lombard L., Crous P.W. (2017). Families, genera, and species of Botryosphaeriales. Fungal Biol..

[9] Phillips A.J., Hyde K.D., Alves A., Liu J.K. (2019). Families in Botryosphaeriales, a phylogenetic, morphological and evolutionary perspective. Fungal Divers..

[10] Phillips A.J.L., Alves A., Abdollahzadeh J., Slippers B., Wingfield M.J., Groenewald J.Z., Crous P.W. (2013). The Botryosphaeriaceae, genera and species known from culture. Stud. Mycol..

[11] Cash E.K. (1952). A Record of the Fungi Named by JB Ellis.

[12] Hanlin R.T. (1963). A Revision of the Ascomycetes of Georgia.

[13] Reid D. (1985). An annotated list of some fungi from the Channel Islands, mostly from Jersey. Trans. Br. Mycol. Soc..

[14] Dissanayake A.J., Camporesi E., Hyde K.D., Phillips A.J.L., Fu C.Y., Yan J.Y., Li X.H. (2016). *Dothiorella* species associated with woody hosts in Italy. Mycosphere.

[15] Bhunjun C.S., Phukhamsakda C., Jayawardena R.S., Jeewon R., Promputtha I., Hyde K.D. (2021). Investigating species boundaries in *Colletotrichum*. Fungal Divers..

[16] Liu F., Ma Z.Y., Hou L.W., Diao Y.Z., Wu W.P., Damm U., Song S., Cai L. (2022). Updating species diversity of *Colletotrichum*, with a phylogenomic overview. Stud. Mycol..

[17] Cannon P.F., Damm U., Johnston P.R., Weir B.S. (2012). *Colletotrichum* current status and future directions. Stud. Mycol..

[18] Blain W.L. (1931). A list of diseases of economic plants in Alabama. Mycologia.

[19] Lee H.B., Park J.Y., Jung H.S. (2005). First report of leaf anthracnose caused by *Colletotrichum boninense* on spindle trees. Plant Pathol..

[20] Alizadeh A., Javan-Nikkhah M., Zare R., Fotouhifar K.B., Damm U., Stukenbrock E.H. (2015). New records of *Colletotrichum* species for the mycobiota of Iran. Mycol. Iran..

[21] Wang T.T., Li P.L., Li H.W., Li J., Gao F., Liu D., Yan J.M., Gong G.S. (2018). First report of *Colletotrichum gloeosporioides* sensu stricto causing anthracnose on *Nandina domestica* in Sichuan Province of China. Plant Dis..

[22] Wu M. (2020). First report of *Colletotrichum siamense* causing Anthracnose on *Euonymus japonicus* in China. Plant Dis..

[23] Senanayake I.C., Crous P.W., Groenewald J.Z., Maharachchikumbura S.S., Jeewon R., Phillips A.J., Bhat J.D., Perera R.H., Li Q.R., Li W.J. (2017). Families of Diaporthales based on morphological and phylogenetic evidence. Stud. Mycol..

[24] Yang Q., Jiang N., Tian C.M. (2020). Three new *Diaporthe* species from Shaanxi province, China. MycoKeys.

[25] Yang Q., Jiang N., Tian C.M. (2021). New species and records of *Diaporthe* from Jiangxi Province, China. MycoKeys.

[26] Gao H., Pan M., Tian C.M., Fan X.L. (2021). *Cytospora* and *Diaporthe* species associated with hazelnut canker and dieback in Beijing, China. Front. Cell. Infect. Microbiol..

[27] Jiang N., Voglmayr H., Bian D.R., Piao C.G., Wang S.K., Li Y. (2021). Morphology and phylogeny of *Gnomoniopsis* (Gnomoniaceae, Diaporthales) from Fagaceae leaves in China. J. Fungi.

[28] Jiang N., Voglmayr H., Piao C.G., Li Y. (2021). Two new species of *Diaporthe* (*Diaporthaceae*, *Diaporthales*) associated with tree cankers in The Netherlands. MycoKeys.

[29] Bai Y.K., Pan M., Gao H., Lin L., Tian C.M., Fan X.L. (2022). Studies of *Diaporthe* species causing hazelnut canker disease in Beijing, China, with two new species described. Plant Pathol..

[30] Simonyan S.A. (1981). Mycoflora of Botanical Gardens and Arboretums of the Armenian SSR (Translated from Russian).

[31] Ahmad S., Iqbal S.H., Khalid A.N. (1997). Fungi of Pakistan.

[32] Fan X.L., Bezerra J.D.P., Tian C.M., Crous P.W. (2020). *Cytospora* (*Diaporthales*) in China. Pers.-Mol. Phylogeny Evol. Fungi.

[33] Zhou X., Pan M., Li H., Tian C.M., Fan X.L. (2020). Dieback of *Euonymus alatus* (*Celastraceae*) Caused by *Cytospora haidianensis* sp. nov. in China. Forests.

[34] Wehmeyer L.E. (1933). The Genus Diaporthe Nitschke and Its Segregates.

[35] Otani Y. (1995). Mycological Flora of Japan.

[36] Mulenko W., Majewski T., Ruszkiewicz-Michalska M. (2008). A preliminary checklist of *Micromycetes* in Poland. W. Szafer Institute of Botany. Pol. Acad. Sci..

[37] Gajanayake A.J., Abeywickrama P.D., Jayawardena R.S., Camporesi E., Bundhun D. (2020). Pathogenic *Diaporthe* from Italy and the first report of *D. foeniculina* associated with *Chenopodium* sp.. Plant Pathol. Quar..

[38] Steyaert R.L. (1949). Contribution à l’étude monographique de *Pestalotia* de Not. et *Monochaetia* Sacc. (*Truncatella* gen. nov. et *Pestalotiopsis* gen. nov.). Bull. Jard. Bot. Brux..

[39] Jiang N., Voglmayr H., Xue H., Piao C.G., Li Y. (2022). Morphology and phylogeny of *Pestalotiopsis* (Sporocadaceae, Amphisphaeriales) from Fagaceae leaves in China. Microbiol. Spectr..

[40] Nag Raj T.R. (1993). Coelomycetous Anamorphs with Appendage-Bearing Conidia.

[41] Kobayashi T., Watanabe K., Ono Y., Furukawa T. (2010). Index of fungi inhabiting woody plants in Japan-host, distribution and literature. Mycoscience.

[42] Maharachchikumbura S.S., Guo L.D., Chukeatirote E., Bahkali A.H., Hyde K.D. (2011). *Pestalotiopsis*—Morphology, phylogeny, biochemistry and diversity. Fungal Divers..

[43] Maharachchikumbura S.S.N., Guo L.D., Cai L., Chukeatirote E., Wu W.P., Sun X., Crous P.W., Bhat D.J., McKenzie E.H.C., Bahkali A.H. (2012). A multi-locus backbone tree for *Pestalotiopsis*, with a polyphasic characterization of 14 new species. Fungal Divers..

[44] Doyle J.J., Doyle J.L. (1990). Isolation of plant DNA from fresh tissue. Focus.

[45] White T.J., Bruns T., Lee S., Taylor J. (1990). Amplification and direct sequencing of fungal ribosomal RNA genes for phylogenetics. PCR Protoc. A Guide Methods Appl..

[46] Carbone I., Kohn L. (1999). A method for designing primer sets for speciation studies in filamentous ascomycetes. Mycologia.

[47] Glass N.L., Donaldson G.C. (1995). Development of primer sets designed for use with the PCR to amplify conserved genes from filamentous ascomycetes. Appl. Environ. Microbiol..

[48] Liu Y.L., Whelen S., Hall B.D. (1999). Phylogenetic relationships among ascomycetes, evidence from an RNA polymerase II subunit. Mol. Biol. Evol..

[49] Guerber J.C., Liu B., Correll J.C., Johnston P.R. (2003). Characterization of diversity in *Colletotrichum acutatum* sensu lato by sequence analysis of two gene introns, mtDNA and intron RFLPs, and mating compatibility. Mycologia.

[50] Crous P.W., Groenewald J.Z., Risède J.M., Simoneau P., Hywel-Jones N.L. (2004). *Calonectria* species and their *Cylindrocladium* anamorphs, species with sphaeropedunculate vesicles. Stud. Mycol..

[51] Lin L., Pan M., Tian C.M., Fan X.L. (2022). Fungal Richness of *Cytospora* species associated with willow canker disease in China. J. Fungi.

[52] Lin L., Pan M., Bezerra J., Tian C.M., Fan X.L. (2022). Re-evaluation of the fungal diversity and pathogenicity of *Cytospora* species from *Populus* in China. Plant Dis..

[53] Katoh K., Standley D.M. (2013). MAFFT multiple sequence alignment software version 7: Improvements in performance and usability. Mol. Biol. Evol..

[54] Tamura K., Stecher G., Peterson D., Filipski A., Kumar S. (2013). MEGA 6: Molecular evolutionary genetics analysis version 6.0. Mol. Biol. Evol..

[55] Guindon S., Dufayard J.F., Lefort V., Anisimova M., Hordijk W., Gascuel H.O. (2010). New algorithms and methods to estimate maximum-likelihood phylogenies, assessing the performance of PhyML 3.0. Syst. Biol..

[56] Ronquist F., Huelsenbeck J.P. (2003). MrBayes 3, Bayesian phylogenetic inference under mixed models. Bioinformatics.

[57] Nouri M.T., Lawrence D.P., Holland L.A., Doll D.A., Kallsen C.E., Culumber C.M., Nylander J.A.A. (2004). MrModeltest v2. Distributed by the Author.

[58] Rambaut A., Drummond A. (2010). FigTree v. 1.3.1. Institute of Evolutionary Biology.

[59] Rayner R.W. (1970). A Mycological Colour Chart.

[60] Wickham H. (2016). ggplot2, Elegant Graphics for Data Analysis.

[61] Kassambara A. (2018). ggpubr: ‘ggplot2’ Based Publication Ready Plots. https://CRAN.R-project.org/package=ggpubr.

[62] Mapook A., Hyde K.D., McKenzie E.H., Jones E.B., Bhat D.J., Jeewon R., Stadler M., Milan C., Samarakoon M.C., Malaithong M. (2020). Taxonomic and phylogenetic contributions to fungi associated with the invasive weed *Chromolaena odorata* (Siam weed). Fungal Divers..

[63] Spegazzini C. (1882). Fungi argentini additis nonnullis brasiliensibus montevideensibusque. Anal. Soc. Cient. Argent..

[64] Jami F., Slippers B., Wingfield M.J., Gryzenhout M. (2014). Botryosphaeriaceae species overlap on four unrelated, native South African hosts. Fungal Biol..

[65] Fan X.L., Yang Q., Cao B., Liang Y.M., Tian C.M. (2015). New records of *Aplosporella javeedii* on five hosts in China based on multi-gene analysis and morphology. Mycotaxon.

[66] Jami F., Wingfield M.J., Gryzenhout M., Slippers B. (2017). Diversity of tree-infecting Botryosphaeriales on native and non-native trees in South Africa and Namibia. Australas. Plant Pathol..

[67] Jiang N., Li J., Piao C.G., Guo M.W., Tian C.M. (2018). Identification and characterization of chestnut branch-inhabiting melanocratic fungi in China. Mycosphere.

[68] Zhu H.Y., Tian C.M., Fan X.L. (2018). Studies of botryosphaerialean fungi associated with canker and dieback of tree hosts in Dongling Mountain of China. Phytotaxa.

[69] Pan M., Zhu H.Y., Bezerra J.D.P., Bonthond G., Tian C.M., Fan X.L. (2019). Botryosphaerialean fungi causing canker and dieback of tree hosts from Mount Yudu in China. Mycol. Prog..

[70] Damm U., Fourie P.H., Crous P.W. (2007). *Aplosporella prunicola*, a novel species of anamorphic Botryosphaeriaceae. Fungal Divers..

[71] Cesati V., De Notaris G. (1863). Schema di classificazione degle sferiacei italici aschigeri piu’ o meno appartenenti al genere Sphaeria nell’antico significato attribuitoglide Persono. Comment. Della Soc. Crittogamolog. Ital..

[72] Barr M.E. (1972). Preliminary studies on the Dothideales in temperate North America. Mich. Univ. Herb Contrib..

[73] Fries E.M. (1823). Systema Mycologicum.

[74] Slippers B., Crous P.W., Denman S., Coutinho T.A., Wingfield B.D., Wingfield M.J. (2004). Combined multiple gene genealogies and phenotypic characters differentiate several species previously identified as *Botryosphaeria dothidea*. Mycologia.

[75] Phillips A.J.L., Lucas M.T. (1997). The taxonomic status of *Macrophoma flaccida* and *Macrophoma reniformis* and their relationship to *Botryosphaeria dothidea*. Sydowia.

[76] Phookamsak R., Hyde K.D., Jeewon R., Bhat D.J., Jones E.B.G., Maharachchikumbura S.S.N., Raspé O., Karunarathna S.C., Wanasinghe D.N., Hongsanan S. (2019). Fungal diversity notes 929–1035: Taxonomic and phylogenetic contributions on genera and species of fungi. Fungal Divers..

[77] Weir B.S., Johnston P.R., Damm U. (2012). The *Colletotrichum gloeosporioides* species complex. Stud. Mycol..

[78] Cannon P.F., Buddie A.G., Bridge P.D. (2008). The typification of *Colletotrichum gloeosporioides*. Mycotaxon.

[79] Prihastuti H., Cai L., Chen H., McKenzie E.H.C., Hyde K.D. (2009). Characterization of *Colletotrichum* species associated with coffee berries in northern Thailand. Fungal Divers..

[80] Chethana K.W.T., Jayawardene R.S., Zhang W., Zhou Y.Y., Liu M., Hyde K.D., Li X.H., Wang J., Zhang K.C., Yan J.Y. (2019). Molecular characterization and pathogenicity of fungal taxa associated with cherry leaf spot disease. Mycosphere.

[81] Mahoney M.J., Tattar T.A. (1980). Identification, etiology, and control of *Euonymus fortunei* anthracnose caused by *Colletotrichum gloeosporiodes*. Plant Dis..

[82] Li P.L., Li J., Zhan X.Y., Liu D., Gong G.S., Chen H.B., Zhang M., Huang Y., Yan J.M. (2017). First report of *Colletotrichum gloeosporioides* sensu stricto causing anthracnose on *Euonymus japonicus* in Sichuan Province of China. Plant Dis..

[83] Pan M., Zhu H.Y., Tian C.M., Huang M., Fan X.L. (2021). Assessment of *Cytospora* isolates from conifer cankers in China, with the descriptions of four new *Cytospora* species. Front. Plant Sci..

[84] Shang Q.J., Hyde K.D., Camporesi E., Maharachchikumbura S.S.N., Norphanphoun C., Brooks S., Liu J.K. (2020). Additions to the genus *Cytospora* with sexual morph in Cytosporaceae. Mycosphere.

[85] Jiang N., Li X.W., Xue H., Piao C.G., Li Y. (2022). Identification of *Kerria japonica* f. *pleniflora* branch canker related fungi based on morphological and molecular methods. J. Terr. Ecosyst. Conserv..

[86] Fan X.L., Hyde K.D., Yang Q., Liang Y.M., Ma R., Tian C.M. (2015). *Cytospora* species associated with canker disease of three anti-desertification plants in northwestern China. Phytotaxa.

[87] Saccardo P.A. (1888). Sylloge Fungorum.

[88] Chen M.M. (2002). Forest Fungi Phytogeography, Forest Fungi Phytogeography of China, North America, and Siberia and International Quarantine of Tree Pathogens.

[89] Zhuang W.Y. (2005). Fungi of Northwestern China.

[90] Fan X.L., Liang Y.M., Ma R., Tian C.M. (2014). Morphological and phylogenetic studies of *Cytospora* (Valsaceae, Diaporthales) isolates from Chinese scholar tree, with description of a new species. Mycoscience.

[91] Tai F.L. (1979). Sylloge Fungorum Sinicorum.

[92] Cooke M.C. (1885). New British Fungi. Grevillea.

[93] Saccardo P.A. (1892). Sylloge Fungorum.

[94] Norphanphoun C., Gentekaki E., Hongsana S., Jayawardena R., Senanayake I.C., Manawasinghe I.S., Abeywickrama P.D., Bhunjun C.S., Hyde K.D. (2022). *Diaporthe*, formalizing the species-group concept. Mycosphere.

[95] Udayanga D., Castlebury L.A., Rossman A.Y., Chukeatirote E., Hyde K.D. (2014). Insights into the genus *Diaporthe*, phylogenetic species delimitation in the *D. eres* species complex. Fungal Divers..

[96] Adams G.C., Wingfield M.J., Common R., Roux J. (2005). Phylogenetic relationships and morphology of *Cytospora* species and related teleomorphs (Ascomycota, Diaporthales, Valsaceae) from *Eucalyptus*. Stud. Mycol..

[97] Moyo P., Mostert L., Spies C.F., Damm U., Halleen F. (2018). Diversity of Diatrypaceae species associated with dieback of Grapevines in South Africa, with the description of *Eutypa cremea* sp. nov.. Plant Dis..

[98] Schmidt C.S., Lovecká P., Mrnka L., Vychodilová A., Strejček M., Fenclová M., Demnerová K.J.M.E. (2018). Distinct communities of poplar endophytes on an unpolluted and a risk element-polluted site and their plant growth-promoting potential in vitro. Microb. Ecol..

[99] Tang W., Ding Z., Zhou Z.Q., Wang Y.Z., Guo L.Y. (2012). Phylogenetic and pathogenic analyses show that the causal agent of apple ring rot in China is *Botryosphaeria dothidea*. Plant Dis..

[100] Zhang J.Z., Xu T. (2005). Cytological characteristics of the infection in different species, varieties and organs of persimmon by *Colletotrichum gloeosporioides*. Mycosystema.

[101] Wang Q., Liu Z., He W., Zhang Y. (2019). *Pseudocercospora* spp. from leaf spots of *Euonymus japonicus* in China. Mycosystema.

[102] Huang J.G., Chi M.Y., Sun X.M., Qian H.W., Liang W.X., Zhou X.F. (2017). First Report of Powdery Mildew Caused by *Erysiphe alphitoides* on *Euonymus japonicas* and Its Natural Teleomorph Stage in China. Plant Dis..

[103] Braun U., Cook R.T.A. (2012). Taxonomic Manual of the Erysiphales (Powdery Mildews).

[104] Li C.W., Zhang Y., Liu Y., Kang J.M., Ma X.M., Fu L.L. (2011). First report of powdery mildew caused by *Erysiphe euonymi-japonici* on *Euonymus japonicus* in central China. Plant Dis..

[105] Wu H., Pan Y., Di R., He Q., Rajaofera M.J.N., Liu W., Zheng F., Miao W. (2019). Molecular identification of the powdery mildew fungus infecting rubber trees in China. For. Pathol..

