# Supplementary material for: The Potential Fungal Pathogens of Euonymus japonicus in Beijing, China
Source: J Fungi (Basel). 2023 Feb 18;9(2):271. doi: 10.3390/jof9020271 (PMC9966606; doi:10.3390/jof9020271)
Supplement: Supplementary file 1 [file jof-09-00271-s001.zip › Table S1.pdf]

Table S1. Strains used in the molecular analyses in this study.

Species	Strain[1]	Host	Origin	GenBank accession numbers								
				ITS	*tef1-α*	*tub2*	*act*	*rpb2*	*gapdh*	*chs-1*	*cal*	*his3*
Aplosporella africana	CBS 121777*	*Acacia mellifera*	Namibia	KF766196	EU101360	–	–	–	–	–	–	–
Aplosporella africana	CBS 1217778*	*Acacia mellifera*	Namibia	EU101316	EU101361	–	–	–	–	–	–	–
Aplosporella artocarpi	CPC 22791*	*Artocarpus heterophyllus*	Thailand	KM006450	KM006481	–	–	–	–	–	–	–
Aplosporella ginkgonis	CFCC 52442*	*Rhus typhina*	China	MH133916	MH133950	–	–	–	–	–	–	–
Aplosporella ginkgonis	CFCC 89661	*Rhus typhina*	China	KM030583	KM030597	–	–	–	–	–	–	–
Aplosporella hesperidica	CBS 732.79*	–	–	KX464083	–	–	–	–	–	–	–	–
Aplosporella hesperidica	CBS 208.37	*Citrus sinensis*	Zimbabwe	JX681069	–	–	–	–	–	–	–	–
Aplosporella hesperidica	**CFCC 55554**	***Euonymus japonicus***	**China**	**OQ344679**	–	–	–	–	–	–	–	–
Aplosporella javeedii	CFCC 50054	*Juniperus chinensis*	China	KP208840	KP208846	–	–	–	–	–	–	–
Aplosporella javeedii	CFCC 50052	*Gleditsia sinensis*	China	KP208838	KP208844	–	–	–	–	–	–	–
Aplosporella javeedii	**CFCC 55489**	***Euonymus japonicus***	**China**	**OQ344672**	**OQ354343**	–	–	–	–	–	–	–
Aplosporella javeedii	**CFCC 55553**	***Euonymus japonicus***	**China**	**OQ344677**	–	–	–	–	–	–	–	–
Aplosporella javeedii	**CFCC 55555**	***Euonymus japonicus***	**China**	**OQ344678**	**OQ354348**	–	–	–	–	–	–	–
Aplosporella macropycnidia	CGMCC 3.17725*	*Cerasus yedoensis*	China	KT343648	KX011176	–	–	–	–	–	–	–
Aplosporella macropycnidia	CGMCC 3.17726	*Cerasus yedoensis*	China	KT343649	KX011177	–	–	–	–	–	–	–
Aplosporella papillata	CBS 121780*	*Acacia tortillas*	South Africa	EU101328	EU101373	–	–	–	–	–	–	–
Aplosporella papillata	CBS 121781	*Acacia tortillas*	South Africa	EU101329	EU101374	–	–	–	–	–	–	–
Aplosporella prunicola	CBS 121167*	*Prunus persica* var. *nucipersica*	South Africa	KF766147	–	–	–	–	–	–	–	–
Aplosporella prunicola	STE-U 6326	*Prunus persica* var. *nucipersica*	South Africa	EF564375	–	–	–	–	–	–	–	–
Aplosporella prunicola	**CFCC 55550**	***Euonymus japonicus***	**China**	**OQ344673**	**OQ354344**	–	–	–	–	–	–	–
Aplosporella prunicola	**CFCC 55551**	***Euonymus japonicus***	**China**	**OQ344674**	**OQ354345**	–	–	–	–	–	–	–

Aplosporella prunicola	**CFCC 55552**	***Euonymus japonicus***	**China**	**OQ344675**	**OQ354346**	–	–	–	–	–	–	–
Aplosporella prunicola	**CFCC 57541**	***Euonymus japonicus***	**China**	**OQ344676**	**OQ354347**	–	–	–	–	–	–	–
Aplosporella sophorae	CPC 29688*	*Sophora microphylla*	New Zealand North	KY173388	–	–	–	–	–	–	–	–
Aplosporella thailandica	MFLU 16-0615*	Dead stems	Thailand	KX423536	KX423537	–	–	–	–	–	–	–
Aplosporella yalgorensis	MUCC511*	*Acacia cochlearis*	Australia	EF591926	EF591977	–	–	–	–	–	–	–
Aplosporella yalgorensis	MUCC512	*Eucalyptus gomphocephala*	Australia	EF591927	EF591978	–	–	–	–	–	–	–
Alanomyces indica	CBS 134264	Soil	India	HF563622	AB872219	–	–	–	–	–	–	–
Botryosphaeria agaves	CBS 133992* = MFLUCC 11-0125	*Agave* sp.	Thailand	JX646791	JX646856	JX646841	–	–	–	–	–	–
Botryosphaeria agaves	CBS 141505 = CPC 26299	*Agave* sp.	France	KX306750	MT592030	MT592463	–	–	–	–	–	–
Botryosphaeria agaves	MFLUCC 10-0051	*Agave* sp.	Thailand	JX646790	JX646855	JX646840	–	–	–	–	–	–
Botryosphaeria corticis	ATCC 22927	*Vaccinium* sp.	USA	DQ299247	EU673291	EU673108	–	–	–	–	–	–
Botryosphaeria corticis	CBS 119047* = CAP 197	*Vaccinium corymbosum*	USA	DQ299245	EU017539	EU673107	–	–	–	–	–	–
Botryosphaeria corticis	CBS 119048 = CAP 198	*Vaccinium corymbosum*	USA	DQ299246	EU017540	MT592464	–	–	–	–	–	–
Botryosphaeria dothidea	CBS 110302	*Vitis vinifera*	Portugal	AY259092	AY573218	EU673106	–	–	–	–	–	–
Botryosphaeria dothidea	CBS 110484	*Vitis vinifera*	Argentina	MT587330	AY343350	MT592466	–	–	–	–	–	–
Botryosphaeria dothidea	CBS 115476*	*Prunus* sp.	Switzerland	AY236949	AY236898	AY236927	–	–	–	–	–	–
Botryosphaeria dothidea	CBS 121557	*Olea europea*	Italy	EF638747	EF638727	MT592467	–	–	–	–	–	–
Botryosphaeria dothidea	CBS 121769	*Acacia mellifera*	Namibia	EU101303	EU101348	–	–	–	–	–	–	–
Botryosphaeria dothidea	CBS 123530	*Diospyros glabra*	South Africa	MT587329	MT592031	MT592465	–	–	–	–	–	–

Botryosphaeria dothidea	CBS 145971	*Grevillea* sp.	Australia	MT587332	MT592034	MT592470	–	–	–	–	–	–
Botryosphaeria dothidea	CERC 1976	English walnut	China	KR261706	KR261718	KR261730	–	–	–	–	–	–
Botryosphaeria dothidea	CERC 2298 = CGMCC 3.18744	*Cedrus deodara*	China	KX278002	KX278107	KX278211	–	–	–	–	–	–
Botryosphaeria dothidea	CERC 2299 = CGMCC 3.18745	*Cedrus deodara*	China	KX278003	KX278108	KX278212	–	–	–	–	–	–
Botryosphaeria dothidea	CGMCC 3.17723	*Morus* sp.	China	KT343254	KU221233	KX197107	–	–	–	–	–	–
Botryosphaeria dothidea	CGMCC 3.17724	*Juglans regia*	China	KT343256	KU221234	KX197108	–	–	–	–	–	–
Botryosphaeria dothidea	CMW 25215	*Mangifera indica*	South Africa	KU997394	KU997130	KU997568	–	–	–	–	–	–
Botryosphaeria dothidea	CMW 7999	*Ostrya* sp.	Switzerland	AY236948	AY236897	AY236926	–	–	–	–	–	–
Botryosphaeria dothidea	DAR 78224	*Vitis vinifera*	Australia	EU137878	EU13788	EU137880	–	–	–	–	–	–
Botryosphaeria dothidea	GZCC 16-0013	Dead wood	China	KX447675	KX447678	–	–	–	–	–	–	–
Botryosphaeria dothidea	GZCC 16-0014	Dead wood	China	KX447676	KX447679	–	–	–	–	–	–	–
Botryosphaeria dothidea	MFLUCC 14-0459	*Quercus* sp.,	Italy	KU848199	–	–	–	–	–	–	–	–
Botryosphaeria dothidea	MUCC 500	*Eucalyptus marginata*	Australia	EF591915	EF591968	EF591951	–	–	–	–	–	–
Botryosphaeria dothidea	MUCC 501	*Eucalyptus marginata*	Australia	EF591916	EF591969	EF591952	–	–	–	–	–	–
Botryosphaeria dothidea	MUCC 503	*Acacia rostellifera*	Australia	EF591918	EF591970	EF591953	–	–	–	–	–	–
Botryosphaeria dothidea	PD 296 = WAC 12404 = WA7	*Eucalyptus calophylla*	Australia	GU251104	GU251236	GU251764	–	–	–	–	–	–
Botryosphaeria dothidea	**CFCC 55490**	***Euonymus japonicus***	**China**	**OQ344707**	**OQ410515**	**OQ410536**	–	–	–	–	–	–
Botryosphaeria dothidea	**CFCC 55491**	***Euonymus japonicus***	**China**	**OQ344708**	**OQ410516**	**OQ410537**	–	–	–	–	–	–

Botryosphaeria dothidea	**CFCC 55492**	***Euonymus japonicus***	**China**	**OQ344695**	**OQ410503**	**OQ410524**	–	–	–	–	–	–
Botryosphaeria dothidea	**CFCC 55493**	***Euonymus japonicus***	**China**	**OQ344696**	**OQ410504**	**OQ410525**	–	–	–	–	–	–
Botryosphaeria dothidea	**CFCC 55494**	***Euonymus japonicus***	**China**	**OQ344697**	**OQ410505**	**OQ410526**	–	–	–	–	–	–
Botryosphaeria dothidea	**CFCC 55495**	***Euonymus japonicus***	**China**	**OQ344698**	**OQ410506**	**OQ410527**	–	–	–	–	–	–
Botryosphaeria dothidea	**CFCC 55496**	***Euonymus japonicus***	**China**	**OQ344694**	**OQ410502**	**OQ410523**	–	–	–	–	–	–
Botryosphaeria dothidea	**CFCC 55564**	***Euonymus japonicus***	**China**	**OQ344711**	**OQ410519**	**OQ410540**	–	–	–	–	–	–
Botryosphaeria dothidea	**CFCC 55565**	***Euonymus japonicus***	**China**	**OQ344713**	**OQ410521**	**OQ410542**	–	–	–	–	–	–
Botryosphaeria dothidea	**CFCC 55566**	***Euonymus japonicus***	**China**	**OQ344706**	**OQ410514**	**OQ410535**	–	–	–	–	–	–
Botryosphaeria dothidea	**CFCC 55567**	***Euonymus japonicus***	**China**	**OQ344704**	**OQ410512**	**OQ410533**	–	–	–	–	–	–
Botryosphaeria dothidea	**CFCC 55568**	***Euonymus japonicus***	**China**	**OQ344705**	**OQ410513**	**OQ410534**	–	–	–	–	–	–
Botryosphaeria dothidea	**CFCC 55569**	***Euonymus japonicus***	**China**	**OQ344699**	**OQ410507**	**OQ410528**	–	–	–	–	–	–
Botryosphaeria dothidea	**CFCC 55570**	***Euonymus japonicus***	**China**	**OQ344700**	**OQ410508**	**OQ410529**	–	–	–	–	–	–
Botryosphaeria dothidea	**CFCC 55571**	***Euonymus japonicus***	**China**	**OQ344701**	**OQ410509**	**OQ410530**	–	–	–	–	–	–
Botryosphaeria dothidea	**CFCC 55572**	***Euonymus japonicus***	**China**	**OQ344702**	**OQ410510**	**OQ410531**	–	–	–	–	–	–
Botryosphaeria dothidea	**CFCC 55575**	***Euonymus japonicus***	**China**	**OQ344709**	**OQ410517**	**OQ410538**	–	–	–	–	–	–
Botryosphaeria dothidea	**CFCC 55576**	***Euonymus japonicus***	**China**	**OQ344710**	**OQ410518**	**OQ410539**	–	–	–	–	–	–
Botryosphaeria dothidea	**CFCC 55577**	***Euonymus japonicus***	**China**	**OQ344712**	**OQ410520**	**OQ410541**	–	–	–	–	–	–
Botryosphaeria dothidea	**CFCC 55578**	***Euonymus japonicus***	**China**	**OQ344714**	**OQ410522**	**OQ410543**	–	–	–	–	–	–

Botryosphaeria dothidea	**CFCC 55618**	***Euonymus japonicus***	**China**	**OQ344703**	**OQ410511**	**OQ410532**	–	–	–	–	–	–
Botryosphaeria fabicerciana	CERC 2930	*Eucalyptus* hybrid	China	KX277987	KX278092	KX278196	–	–	–	–	–	–
Botryosphaeria fabicerciana	CERC 2949	*Eucalyptus* hybrid	China	KX277984	KX278089	KX278194	–	–	–	–	–	–
Botryosphaeria fabicerciana	CERC 3441	*Eucalyptus* hybrid	China	KX277974	KX278079	KX278184	–	–	–	–	–	–
Botryosphaeria fabicerciana	CERC 3469	*Eucalyptus* hybrid	China	KX277975	KX278080	KX278185	–	–	–	–	–	–
Botryosphaeria fabicerciana	CPC 29629	*Mangifera indica*	USA	MT587331	MT592033	MT592469	–	–	–	–	–	–
Botryosphaeria fabicerciana	MFLUCC 10-0098*	*Entada* sp.	Thailand	JX646789	JX646854	JX646839	–	–	–	–	–	–
Botryosphaeria fabicerciana	MFLUCC 11-0507	*Entada* sp.	Thailand	JX646788	JX646853	JX646838	–	–	–	–	–	–
Botryosphaeria kuwatsukai	CBS 135219* = PG 2	*Malus domestica*	China	KJ433388	KJ433410	–	–	–	–	–	–	–
Botryosphaeria kuwatsukai	CGMCC 3.18007	*Malus* sp.	China	KX197074	KX197094	KX197101	–	–	–	–	–	–
Botryosphaeria kuwatsukai	CGMCC 3.18008	*Amygdalus* sp.	China	KX197075	KX197095	KX197102	–	–	–	–	–	–
Botryosphaeria kuwatsukai	LSP 5	*Pyrus* sp.	China	KJ433395	KJ433417	–	–	–	–	–	–	–
Botryosphaeria qingyuanensis	CERC 2946 = CGMCC 3.18742*	*Eucalyptus* hybrid	China	KX278000	KX278105	KX278209	–	–	–	–	–	–
Botryosphaeria qingyuanensis	CERC 2947 = CGMCC 3.18743	*Eucalyptus* hybrid	China	KX278001	KX278106	KX278210	–	–	–	–	–	–
Botryosphaeria ramosa	CBS 122069* = CMW 26167	*Eucalyptus camaldulensis*	Australia	EU144055	EU144070	KF766132	–	–	–	–	–	–
Botryosphaeria ramosa	CERC 1999	*Eucalyptus* hybrid	China	KX277988	KX278093	KX278197	–	–	–	–	–	–
Botryosphaeria ramosa	CERC 2001 = CGMCC 3.187396*	*Eucalyptus* hybrid	China	KX277989	KX278094	KX278198	–	–	–	–	–	–
Botryosphaeria scharifii	CBS 124702 = IRAN 1543C	*Mangifera indica*	Iran	JQ772019	JQ772056	–	–	–	–	–	–	–
Botryosphaeria scharifii	CBS 124703* = IRAN 1529C	*Mangifera indica*	Iran	JQ772020	JQ772057	–	–	–	–	–	–	–

Cophinforma eucalypti	CBS 134651 = MFLUCC 11-0425	*Eucalyptus* sp.	Thailand	JX646800	JX646865	JX64684	–	–	–	–	–	–
Dothiorella acacicola	CBS 141295* = CPC 26349	*Acacia mearnsii*	France	KX228269	KX228376	–	–	–	–	–	–	–
Dothiorella acericola	HNXX032	*Ziziphus jujuba*	China	KY385661	KY393212	KY393178	–	–	–	–	–	–
Dothiorella acericola	KUMCC 18-0137*	*Acer palmatum*	China	MK359449	MK361182	–	–	–	–	–	–	–
Dothiorella acericola	**CFCC 55556**	***Euonymus japonicus***	**China**	**OQ344756**	**OQ410574**	**OQ410578**	–	–	–	–	–	–
Dothiorella acericola	**CFCC 55558**	***Euonymus japonicus***	**China**	**OQ344757**	**OQ410575**	**OQ410579**	–	–	–	–	–	–
Dothiorella acericola	**CFCC 55559**	***Euonymus japonicus***	**China**	**OQ344758**	**OQ410576**	**OQ410580**	–	–	–	–	–	–
Dothiorella acericola	**CFCC 55561**	***Euonymus japonicus***	**China**	**OQ344759**	**OQ410577**	**OQ410581**	–	–	–	–	–	–
Dothiorella alpina	CGMCC 3.18001*	*Platycladus orientalis*	China	KX499645	KX499651	–	–	–	–	–	–	–
Dothiorella brevicollis	CBS 130411* = CMW 36463	*Acacia karroo*	South Africa	JQ239403	JQ239390	JQ239371	–	–	–	–	–	–
Dothiorella brevicollis	CBS 130412 = CMW 36464	*Acacia karroo*	South Africa	MT587395	MT592107	MT59257	–	–	–	–	–	–
Dothiorella californica	CBS 119635	*Laurus nobilis*	Turkey	MT587396	MT592108	MT592579	–	–	–	–	–	–
Dothiorella californica	CBS 141587*	*Umbellularia californica*	USA	KX357188	KX357211	KX357165	–	–	–	–	–	–
Dothiorella capri-amissi	CBS 121763*	*Acacia erioloba*	South Africa	EU101323	EU101368	KX464850	–	–	–	–	–	–
Dothiorella capri-amissi	CBS 121878*	*Acacia erioloba*	South Africa	EU101324	EU101369	KX464851	–	–	–	–	–	–
Dothiorella casuarinae	CBS 120688*	*Casuarina* sp.	Australia	DQ846773	DQ875331	DQ875340	–	–	–	–	–	–
Dothiorella casuarinae	CBS 120689	*Casuarina* sp.	Australia	DQ846772	DQ875332	DQ875339	–	–	–	–	–	–
Dothiorella casuarinae	CBS 120690	*Casuarina* sp.	Australia	DQ846774	DQ875333	DQ875341	–	–	–	–	–	–
Dothiorella citricola	CBS 124728	*Citrus cinensis*	New Zealand	EU673322	EU673289	KX464852	–	–	–	–	–	–
Dothiorella citricola	CBS 124729*	*Citrus cinensis*	New Zealand	EU673323	EU673290	KX464853	–	–	–	–	–	–
Dothiorella citricola	CBS 130415	*Acacia karroo*	South Africa	MT587397	MT592109	MT592580	–	–	–	–	–	–
Dothiorella diospyricola	CBS 145972*	*Diospyros mespiliformis*	South Africa	MT587398	MT592110	MT592581	–	–	–	–	–	–
Dothiorella dulcispinae	CBS 121764	*Acacia mellifera*	Namibia	EU101299	EU101344	KX464854	–	–	–	–	–	–
Dothiorella dulcispinae	CBS 121765*	*Acacia mellifera*	South Africa	EU101300	EU101345	KX464862	–	–	–	–	–	–
Dothiorella dulcispinae	CBS 121766	*Acacia mellifera*	South Africa	EU101301	EU101346	KX464863	–	–	–	–	–	–
Dothiorella dulcispinae	CBS 130413	*Acacia karroo*	South Africa	JQ239400	JQ239387	JQ239373	–	–	–	–	–	–
Dothiorella dulcispinae	CBS 130414	*Acacia karroo*	South Africa	MT587408	MT592120	MT592598	–	–	–	–	–	–
Dothiorella eriobotryae	CBS 140852*	*Eriobotrya japonica*	Spain	KT240287	KT240262	MT592582	–	–	–	–	–	–

Dothiorella eriobotryae	CBS 145976	*Rhamnus saxatilis*	Austria	MT587399	MT592111	MT592583	–	–	–	–	–	–
Dothiorella eriobotryae	MFLUCC 14-0902	*Rhamnus cathartica*	Russia	KU246381	–	–	–	–	–	–	–	–
Dothiorella guttulata	CBS 134886	*Cotinus coggygria*	Slovenia	FM955384	FM955416	MT592584	–	–	–	–	–	–
Dothiorella guttulata	CBS 134888	*Ostrya carpinifolia*	Italy	FM955386	FM955420	MT592585	–	–	–	–	–	–
Dothiorella guttulata	MFLUCC 17-0242*	*Alnus glutinosa*	Italy	KY797637	–	–	–	–	–	–	–	–
Dothiorella iberica	CBS 121001	*Vitis vinifera* cv. Zinfande	USA	MT587420	MT592132	MT592612	–	–	–	–	–	–
Dothiorella iberica	PD257	*Quercus ilex*	Spain	GU251168	GU251300	GU251828	–	–	–	–	–	–
Dothiorella iberica	UCD1448SLO	*Vitis vinifera*	USA	EF202009	EF202023	EF202016	–	–	–	–	–	–
Dothiorella iberica	CBS 113189	*Quercus ilex*	Spain	AY573199	AY573230	KX464855	–	–	–	–	–	–
Dothiorella iberica	CBS 115035	*Quercus ilex*	Spain	AY573213	AY573228	MT592586	–	–	–	–	–	–
Dothiorella iberica	CBS 115036	*Quercus ruber*	Spain	AY573216	AY573233	MT592587	–	–	–	–	–	–
Dothiorella iberica	CBS 115037	*Quercus ruber*	Spain	AY573201	AY573229	MT592588	–	–	–	–	–	–
Dothiorella iberica	CBS 115039	*Quercus* sp	Italy	AY573210	AY573234	MT592589	–	–	–	–	–	–
Dothiorella iberica	CBS 115040	*Quercus ilex*	Spain	AY573214	AY573232	MT592590	–	–	–	–	–	–
Dothiorella iberica	CBS 115041*	*Quercus ilex*	Spain	AY573202	AY573222	EU673096	–	–	–	–	–	–
Dothiorella iberica	CBS 130984	*Cupressus macrocarpa*	Algeria	MT587400	MT592112	MT592591	–	–	–	–	–	–
Dothiorella iberica	CBS 133500	*Persea americana*	USA	MT587410	MT592122	MT592602	–	–	–	–	–	–
Dothiorella iberica	UCR-DI3	Aerial spore trap in almond orchard	USA	KP012591	KP828802	KP067201	–	–	–	–	–	–
Dothiorella iberica	UCROK1227	*Quercus agrifolia*	USA	JQ411405	JQ512114	JQ411436	–	–	–	–	–	–
Dothiorella iberica	UCROK1396	*Quercus agrifolia*	USA	JQ411406	JQ512115	JQ411437	–	–	–	–	–	–
Dothiorella iranica	CBS 124722*	*Olea* sp.,	Iran	KC898231	KC898214	KX464856	–	–	–	–	–	–
Dothiorella italica	CBS 115038	*Malus pumila*	Netherlands	AY573206	AY573223	EU673101	–	–	–	–	–	–
Dothiorella italica	CBS 122805	*Prunus dulcis*	Spain	MT587401	MT592113	MT592592	–	–	–	–	–	–
Dothiorella italica	CBS 122806	*Prunus dulcis*	Spain	MT587402	MT592114	MT592593	–	–	–	–	–	–
Dothiorella italica	CBS 910.73	*Acer pseudoplatanus*	Germany	EU673315	EU673282	EU673139	–	–	–	–	–	–
Dothiorella italica	CPC 30616	*Taxus baccata*	Germany	MT587403	MT592115	MT592594	–	–	–	–	–	–
Dothiorella italica	CPC 33887	*Lonicera tatarica*	Germany	MT587404	MT592116	MT592595	–	–	–	–	–	–
Dothiorella italica	CPC 35506	*Lonicera xylosteum*	Germany	MT587405	MT592117	MT592596	–	–	–	–	–	–
Dothiorella italica	CPC 35540	*Rubus idaeus*	Ukraine	MT587406	MT592118	MT592597	–	–	–	–	–	–
Dothiorella italica	MFLUCC 17-0951*	*Rosa canina*	Italy	MF398891	MF398943	–	–	–	–	–	–	–

Dothiorella lampangensis	MFLUCC 18-0232*	*Rutaceae*	Thailand	MK347758	MK340869	MK412874	–	–	–	–	–	–
Dothiorella longicollis	CBS 122066	*Terminalia* sp.	Australia	EU144052	EU144067	KX464857	–	–	–	–	–	–
Dothiorella longicollis	CBS 122067	*Lysiphyllum cunninghamii*	Australia	EU144053	EU144068	KX464858	–	–	–	–	–	–
Dothiorella longicollis	CBS 122068*	*Lysiphyllum cunninghamii*	Australia	EU144054	EU144069	KF766130	–	–	–	–	–	–
Dothiorella magnoliae	CFCC 51563*	*Magnolia grandiflora*	China	KY111247	KY213686	–	–	–	–	–	–	–
Dothiorella magnoliae	CFCC 51564	*Magnolia grandiflora*	China	KY111248	KY213687	–	–	–	–	–	–	–
Dothiorella mangifericola	CBS 121760*	*Acacia karroo*	Namibia	KF766227	EU101335	KX464877	–	–	–	–	–	–
Dothiorella mangifericola	CBS 121761	*Acacia mellifera*	South Africa	EU101293	EU101338	KX464878	–	–	–	–	–	–
Dothiorella mangifericola	CBS 124726	*Mangifera indica*	Iran	MT587407	MT592119	–	–	–	–	–	–	–
Dothiorella mangifericola	CBS 124727*	*Mangifera indica*	Iran	KC898221	KX464614	–	–	–	–	–	–	–
Dothiorella moneti	WAC 13154* = MUCC 505	*Acacia rostellifera*	Australia	EF591920	EF591971	EF591954	–	–	–	–	–	–
Dothiorella omnivora	CBS 124716	*Juglans regia*	Iran	KC898232	KC898215	KX464864	–	–	–	–	–	–
Dothiorella omnivora	CBS 140349*	*Corylus avellana*	Italy	KP205497	KP205470	–	–	–	–	–	–	–
Dothiorella omnivora	CBS 188.87	*Juglans regia*	France	EU673316	EU673283	EU673119	–	–	–	–	–	–
Dothiorella parva	CBS 124720*	*Corylus* sp.	Iran	KC898234	KC898217	KX464866	–	–	–	–	–	–
Dothiorella parva	CBS 124721	*Corylus* sp.	Iran	KX464123	KX464615	KX464867	–	–	–	–	–	–
Dothiorella parva	CBS 134885	*Ostrya carpinifolia*	Slovenia	FM955391	FM955423	MT592599	–	–	–	–	–	–
Dothiorella parva	CBS 134887	*Ostrya carpinifolia*	Italy	FM955385	FM955417	MT592600	–	–	–	–	–	–
Dothiorella plurivora	CBS 124724*	*Citrus* sp.	Iran	KC898225	KC898208	KX464874	–	–	–	–	–	–
Dothiorella pretoriensis	CBS 130404*	*Acacia karroo*	South Africa	JQ239405	JQ239392	JQ239376	–	–	–	–	–	–
Dothiorella prunicola	CBS 124723*	*Prunus dulcis*	Portugal	EU673313	EU673280	EU673100	–	–	–	–	–	–
Dothiorella santali	WAC 13155*	*Santalum acuminatum*	Australia	EF591924	EF591975	EF591958	–	–	–	–	–	–
Dothiorella sarmentorum	CBS 128309*	*Vitis vinifera*	USA	HQ288218	HQ288262	HQ288297	–	–	–	–	–	–
Dothiorella sarmentorum	CBS 128310	*Vitis vinifera* cv. Chardonel	USA	MH864852	MT592106	MT592577	–	–	–	–	–	–
Dothiorella sarmentorum	CBS 164.33	*Ulmus* sp	Netherlands	KX464127	KX464619	KX464881	–	–	–	–	–	–

Dothiorella sarmentorum	CPC 31043	*Tilia platyphyllos*	Germany	MT587409	MT592121	MT592601	–	–	–	–	–	–
Dothiorella sarmentorum	IMI 63581b*	*Ulmus* sp.	UK	AY573212	AY573235	EU673102	–	–	–	–	–	–
Dothiorella sempervirentis	CBS 124718*	*Cupressus sempervirens*	Iran	KC898236	KC898220	KX464884	–	–	–	–	–	–
Dothiorella sempervirentis	CBS 124719	*Cupressus sempervirens*	Iran	KC898237	KC898219	KX464885	–	–	–	–	–	–
Dothiorella sp. 1	CBS 121783	*Acacia mearnsii*	South Africa	EU101333	EU101378	KX464859	–	–	–	–	–	–
Dothiorella sp. 2	CBS 121784	*Acacia mearnsii*	South Africa	EU101331	EU101376	KX464860	–	–	–	–	–	–
Dothiorella sp. 3	CBS 121785	*Acacia mearnsii*	South Africa	EU101334	EU101379	KX464861	–	–	–	–	–	–
Dothiorella striata	CBS 124730	*Citrus cinensis*	New Zealand	EU673320	EU673287	EU673142	–	–	–	–	–	–
Dothiorella striata	CBS 124731*	*Citrus cinensis*	New Zealand	EU673321	EU673288	EU673143	–	–	–	–	–	–
Dothiorella striata	DAR 80992*	*Vitis vinifera* cv. Chardonnay	Australia	KJ573643	KJ573640	KJ577551	–	–	–	–	–	–
Dothiorella symphoricarpicola	CBS 135622	*Chamaecyparis atlantica*	Serbia	KF261725	KF261729	KF261731	–	–	–	–	–	–
Dothiorella symphoricarpicola	CBS 135623	*Thuja plicata*	Serbia	KF040058	KF261728	KF261730	–	–	–	–	–	–
Dothiorella symphoricarpicola	CBS 135847	*Chamaecyparis atlantica*	Serbia	KF575016	KF575051	KF575112	–	–	–	–	–	–
Dothiorella symphoricarpicola	CPC 30972	*Elaeagnus rhamnoides*	Germany	MT587411	MT592123	MT592603	–	–	–	–	–	–
Dothiorella symphoricarpicola	CPC 30976	*Tilia platyphyllos*	Germany	MT587412	MT592124	MT592604	–	–	–	–	–	–
Dothiorella symphoricarpicola	CPC 31033	*Elaeagnus rhamnoides*	Germany	MT587413	MT592125	MT592605	–	–	–	–	–	–
Dothiorella symphoricarpicola	CPC 33923	*Acer negundo*	Germany	MT587414	MT592126	MT592606	–	–	–	–	–	–
Dothiorella symphoricarpicola	MFLUCC 13-0497*	*Symphoricarpos* sp.	Italy	KJ742378	KJ742381	–	–	–	–	–	–	–
Dothiorella tectonae	MFLUCC 18-0382*	*Tectona grandis*	Thailand	KM396899	KM409637	KM510357	–	–	–	–	–	–
Dothiorella thailandica	CBS 133991*	Dead bamboo culm	Thailand	JX646796	JX646861	JX646844	–	–	–	–	–	–

Dothiorella thripsita	CBS 125445* = BRIP 51876a	*Acacia harpophylla*	Australia	KJ573642	KJ573639	KJ577550	–	–	–	–	–	–
Dothiorella ulmacea	CBS 138855*	*Eucalyptus* sp.	Malaysia	KR611881	KR611910	KR611909	–	–	–	–	–	–
Dothiorella ulmacea	CBS 140005	*Ulmus laevis*	Germany	KR611882	KR857697	MT592607	–	–	–	–	–	–
Dothiorella ulmacea	CBS 141414	*Corylus avellana*	Germany	MT587415	MT592127	MT592608	–	–	–	–	–	–
Dothiorella uruguayensis	CBS 124908*	*Hexachlamis edulis*	Uruguay	EU080923	EU863180	KX464886	–	–	–	–	–	–
Dothiorella vidmadera	CPC 30614	*Ribes rubrum*	Germany	MT587416	MT592128	–	–	–	–	–	–	–
Dothiorella vidmadera	DAR 78992*	*Vitis vinifera*	Australia	EU768874	EU768881	HM800522	–	–	–	–	–	–
Dothiorella vinea-gemmae	CPC 29689	*Sophora microphylla*	New Zealand	MT587417	MT592129	MT592609	–	–	–	–	–	–
Dothiorella vinea-gemmae	DAR 81012*	*Vitis vinifera* cv. Chardonnay	Australia	KJ573644	KJ573641	KJ577552	–	–	–	–	–	–
Dothiorella viticola	CBS 112869	*Vitis vinifera*, vine	South Africa	AY343373	AY343336	KX464869	–	–	–	–	–	–
Dothiorella viticola	CBS 117006	*Vitis vinifera* cv. Garnatxa Negra	Spain	AY905555	AY905562	EU673103	–	–	–	–	–	–
Dothiorella viticola	CBS 117007	*Vitis vinifera* cv. Macabeu	Spain	AY905556	KX464623	KX464890	–	–	–	–	–	–
Dothiorella yunnana	CGMCC 3.17999*	*Camellia* sp	China	KX499643	KX499649	–	–	–	–	–	–	–
Dothiorella yunnana	CGMCC 3.18000	*Camellia* sp	China	KX499644	KX499650	–	–	–	–	–	–	–
Neofusicoccum luteum	CBS 121482	Phaeomoniella chlamydospora	Spain	EU650669	KX464689	KX464967	–	–	–	–	–	–
Colletotrichum aenigma	ICMP 18608*	*Persea americana*	Israel	JX010244	–	JX010389	JX009443	–	JX010044	JX009774	–	–
Colletotrichum aenigma	**CFCC 55535**	***Euonymus japonicus***	**China**	**OQ344719**	–	**OQ410548**	**OQ410632**	–	**OQ410568**	**OQ410558**	–	–
Colletotrichum aeschynomenes	ICMP 17673*, ATCC 201874	*Aeschynomene virginica*	USA	JX010176	–	JX010392	JX009483	–	JX009930	JX009799	–	–
Colletotrichum alatae	CBS 304.67*, ICMP 17919	*Dioscorea alata*	India	JX010190	–	JX010383	JX009471	–	JX009990	JX009837	–	–
Colletotrichum alienum	ICMP 12071*	*Malus domestica*	New Zealand	JX010251	–	JX010411	JX009572	–	JX010028	JX009882	–	–
Colletotrichum aotearoa	ICMP 18537*	*Coprosma* sp.	New Zealand	JX010205	–	JX010420	JX009564	–	JX010005	JX009853	–	–
Colletotrichum arecicola	CGMCC 3.19667*	*Areca catechu*	China	MK914635	–	MK935498	MK935374	–	MK935455	MK935541	–	–

Colletotrichum artocarpicola	MFLUCC 18-1167*	*Artocarpus heterophyllus*	Thailand	MN415991	–	MN435567	MN435570	–	MN435568	MN435569	–	–
Colletotrichum asianum	ICMP 18580*, CBS 130418	*Coffea arabica*	Thailand	FJ972612	–	JX010406	JX009584	–	JX010053	JX009867	–	–
Colletotrichum australianum	VPRI 43075*	*Citrus sinensis*	Australia	MG572138	–	MG572149	MN442109	–	MG572127	MW091987	–	–
Colletotrichum camelliae	CGMCC 3.14925, LC1364*	*Camellia sinensis*	China	KJ955081	–	KJ955230	KJ954363	–	KJ954782	MZ799255	–	–
Colletotrichum changpingense	CGMCC 3.17582*, SA0016, MFLUCC 15-0022	Rhizome of *Fragaria ×ananass*	China	KP683152	–	MZ673952	KP683093	–	MZ664048	KP852449	–	–
Colletotrichum changpingense	MFLUCC 18-0945*	Magnolia garrettii	Thailand	MW346499	–	–	MW655578	–	MW548592	MW623653	–	–
Colletotrichum chrysophilum	URM 7368, CMM 4268*	*Musa* sp.	Brazil	KX094252	–	KX094285	KX093982	–	KX094183	KX094083	–	–
Colletotrichum cigarro	ICMP 18539*	*Olea europaea*	Australia	JX010230	–	JX010434	JX009523	–	JX009966	JX009800	–	–
Colletotrichum clidemiae	ICMP 18658*	*Clidemia hirta*	USA, Hawaii	JX010265	–	JX010438	JX009537	–	JX009989	JX009877	–	–
Colletotrichum cobbittiense	BRIP 66219*	*Cordyline stricta ×C. australis*	Australia	MH087016	–	MH094137	MH094134	–	MH094133	MH094135	–	–
Colletotrichum conoides	CGMCC 3.17615, CAUG17, LC6226*	Chili pepper	China	KP890168	–	KP890174	KP890144	–	KP890162	KP890156	–	–
Colletotrichum cordylinicola	MFLUCC 090551*, ICMP 18579	*Cordyline fruticosa*	Thailand	JX010226	–	JX010440	HM470235	–	JX009975	JX009864	–	–
Colletotrichum dracaenigenum	MFLUCC 19-0430*	*Dracaena* sp.	Thailand	MN921250	–	–	MT313686	–	MT215577	MT215575	–	–
Colletotrichum endophyticum	MFLUCC 13-0418, LC0324*	*Pennisetum purpureum*	Thailand	KC633854	–	MZ67395	KF306258	–	KC832854	MZ799261	–	–
Colletotrichum euonymi	**CFCC 55540**	***Euonymus japonicus***	**China**	**OQ344715**	–	**OQ410544**	**OQ410628**	–	**OQ410564**	**OQ410554**	–	–
Colletotrichum euonymi	**CFCC 55537**	***Euonymus japonicus***	**China**	**OQ344716**	–	**OQ410545**	**OQ410629**	–	**OQ410565**	**OQ410555**	–	–
Colletotrichum euonymi	**CFCC 55483**	***Euonymus japonicus***	**China**	**OQ344717**	–	**OQ410546**	**OQ410630**	–	**OQ410566**	**OQ410556**	–	–
Colletotrichum euonymi	**CFCC 55542***	***Euonymus japonicus***	**China**	**OQ344718**	–	**OQ410547**	**OQ410631**	–	**OQ410567**	**OQ410557**	–	–

Colletotrichum euonymicola	**CFCC 55486***	***Euonymus japonicus***	**China**	**OQ344723**	–	**OQ410552**	**OQ410636**	–	**OQ410572**	**OQ410562**	–	–
Colletotrichum euonymicola	**CFCC 55539**	***Euonymus japonicus***	**China**	**OQ344724**	–	**OQ410553**	**OQ410637**	–	**OQ410573**	**OQ410563**	–	–
Colletotrichum fructicola	ICMP 18581*, CBS 130416	*Coffea arabica*	Thailand	JX010165	–	JX010405	FJ907426	–	JX010033	JX009866	–	–
Colletotrichum fructivorum	Coll1414, CBS 133125*	*Vaccinium macrocarpon*	Burlington	JX145145	–	JX145196	MZ664126	–	MZ664047	MZ799259	–	–
Colletotrichum gloeosporioides	IMI 356878*, CBS 112999	*Citrus sinensis*	Italy	JX010152	–	JX010445	JX009531	–	JX010056	JX009818	–	–
Colletotrichum gloeosporioides	**CFCC 55544**	***Euonymus japonicus***	**China**	**OQ344720**	–	**OQ410549**	**OQ410633**	–	**OQ410569**	**OQ410559**	–	–
Colletotrichum gloeosporioides	**CFCC 55545**	***Euonymus japonicus***	**China**	**OQ344721**	–	**OQ410550**	**OQ410634**	–	**OQ410570**	**OQ410560**	–	–
Colletotrichum gloeosporioides	**CFCC 55547**	***Euonymus japonicus***	**China**	**OQ344722**	–	**OQ410551**	**OQ410635**	–	**OQ410571**	**OQ410561**	–	–
Colletotrichum grevilleae	CBS 132879, CPC 15481*	*Grevillea* sp.	Italy	KC297078	–	KC297102	KC296941	–	KC297010	KC296987	–	–
Colletotrichum grossum	CGMCC3.17614, LC6227*	Chili pepper	China	KP890165	–	KP890171	KP890141	–	KP890159	KP890153	–	–
Colletotrichum hebeiense	MFLUCC13–0726*	*Vitis vinifera*	China	KF156863	–	KF288975	KF377532	–	KF377495	KF289008	–	–
Colletotrichum hederiicola	MFLU 15-0689*	*Hedera helix*	Italy	MN631384	–	–	MN635795	–	–	MN635794	–	–
Colletotrichum helleniense	CBS 142418, CPC 26844*	*Poncirus trifoliata*	Greece, Arta	KY856446	–	KY856528	KY856019	–	KY856270	KY856186	–	–
Colletotrichum henanense	CGMCC 3.17354, LF238 *	*Camellia sinensis*	China	KJ955109	–	KJ955257	KM023257	–	KJ954810	MZ799256	–	–
Colletotrichum horii	NBRC 7478*, ICMP 10492, MTCC 10841	*Diospyros kaki*	Japan	GQ329690	–	JX010450	JX009438	–	GQ329681	JX009752	–	–
Colletotrichum hystricis	CBS 142411, CPC 28153*	*Citrus hystrix*	Italy, Catania	KY856450	–	KY856532	KY856023	–	KY856274	KY856190	–	–
Colletotrichum jiangxiense	CGMCC 3.17361*, LC3266, LF488	*Camellia sinensis*	China	KJ955149	–	OK236389	KJ954427	–	KJ954850	MZ799257	–	–

Colletotrichum kahawae	IMI 319418*, ICMP 17816	*Coffea arabica*	Kenya	JX010231	–	JX010444	JX009452	–	JX010012	JX009813	–	–
Colletotrichum makassarense	CBS 143664*	*Capsicum annuum*	Indonesia	MH728812	–	MH846563	MH781480	–	MH728820	MH805850	–	–
Colletotrichum musae	CBS 116870*, ICMP 19119, MTCC 11349	*Musa* sp.	USA	JX010146	–	HQ596280	JX009433	–	JX010050	JX009896	–	–
Colletotrichum nupharicola	CBS 470.96*, ICMP 18187	*Nuphar lutea* subsp. *polysepala*	USA	JX010187	–	JX010398	JX009437	–	JX009972	JX009835	–	–
Colletotrichum pandanicola	MFLUCC 17-0571*	*Pandanaceae*	Thailand	MG646967	–	MG646926	MG646938	–	MG646934	MG646931	–	–
Colletotrichum perseae	CBS 141365*, GA100	Avocado	Israel	KX620308	–	KX620341	KX620145	–	KX620242	MZ799260	–	–
Colletotrichum proteae	CBS 132882*, CPC 14859	*Protea* sp.	South Africa	KC297079	–	KC297101	KC296940	–	KC297009	KC296986	–	–
Colletotrichum pseudotheobromicola	MFLUCC 18–1602*	*Prunus avium*	China	MH817395	–	MH853684	MH853681	–	MH853675	MH853678	–	–
Colletotrichum psidii	CBS 145.29*, ICMP 19120	*Psidium* sp.	Italy	JX010219	–	JX010443	JX009515	–	JX009967	JX009901	–	–
Colletotrichum queenslandicum	ICMP 1778*	*Carica papaya*	Australia	JX010276	–	JX010414	JX009447	–	JX009934	JX009899	–	–
Colletotrichum rhexiae	Coll1026, BPI 884112, CBS 133134*	*Rhexia virginica*	Sussex	JX145128	–	JX145179	MZ664127	–	MZ664046	MZ799258	–	–
Colletotrichum salsolae	ICMP 19051*	*Salsola tragus*	Hungary	JX010242	–	JX010403	JX009562	–	JX009916	JX009863	–	–
Colletotrichum siamense	ICMP 18578*, CBS 130417	*Coffea arabica*	Thailand	JX010171	–	JX010404	FJ907423	–	JX009924	JX009865	–	–
Colletotrichum syzygiicola	DNCL021, MFLUCC 10-0624*	*Syzygium samarangense*	Thailand	KF242094	–	KF254880	KF157801	–	KF242156	–	–	–
Colletotrichum tainanense	CBS 143666*	*Capsicum annuum*	Taiwan	MH728818	–	MH846558	MH781475	–	MH728823	MH805845	–	–
Colletotrichum temperatum	CBS 133122*, BPI 884100	*Vaccinium macrocarpon*	Bronx	JX145159	–	JX145211	MZ664125	–	MZ664045	MZ799254	–	–
Colletotrichum theobromicola	CBS 124945*, ICMP 18649	*Theobroma cacao*	Panama	JX010294	–	JX010447	JX009444	–	JX010006	JX009869	–	–
Colletotrichum ti	ICMP 4832*	*Cordyline* sp.	New Zealand	JX010269	–	JX010442	JX009520	–	JX009952	JX009898	–	–

Colletotrichum tropicale	CBS 124949*, ICMP 18653, MTCC 11371	*Theobroma cacao*	Panama	JX010264	–	JX010407	JX009489	–	JX010007	JX009870	–	–
Colletotrichum viniferum	GZAAS 5.08601*, yg1	*Vitis vinifera* cv. Shuijing	China	JN412804	–	–	JN412795	–	JN412798	–	–	–
Colletotrichum wuxiense	CGMCC 3.17894*	*Camellia sinensis*	China	KU251591	–	KU252200	KU251672	–	KU252045	KU251939	–	–
Colletotrichum xanthorrhoeae	BRIP 45094*, ICMP 17903, CBS 127831	*Xanthorrhoea preissii*	Australia	JX010261	–	JX010448	JX009478	–	JX009927	JX009823	–	–
Colletotrichum xishuangbannaense	MFLUCC 19-0107*	*Magnolia liliifera*	China	MW346469	–	–	MW652294	–	MW537586	MW660832	–	–
Colletotrichum yulongense	CFCC 50818*	*Vaccinium dunalianum* var. *urophyllum*	China	MH751507	–	MK108987	MH777394	–	MK108986	MH793605	–	–
Colletotrichum vietnamense	CBS 125477, BMT25(L3)	*Coffea* sp.	Vietnam	KF687720	–	KF687876	KF687791	–	KF687831	KF687768	–	–
Colletotrichum vietnamense	CBS 125478, LD16(L2)*	*Coffea* sp.	Vietnam	KF687721	–	KF687877	KF687792	–	KF687832	KF687769	–	–
Cytospora ailanthicola	CFCC 89970	*Ailanthus altissima*	Ningxia, China	MH933618	MH933494	MH933565	MH933526	MH933592	–	–	–	–
Cytospora ailanthicola	**CFCC 55529**	***Euonymus japonicus***	**China**	**OQ344725**	**OQ398738**	**OQ398765**	**OQ410598**	**OQ398712**	–	–	–	–
Cytospora albodisca	CFCC 53161	*Platycladus orientalis*	Beijing, China	MW418406	MW422921	MW422933	MW422899	MW422909	–	–	–	–
Cytospora albodisca	CFCC 54373	*Platycladus orientalis*	Beijing, China	MW418407	MW422922	MW422934	MW422900	MW422910	–	–	–	–
Cytospora albodisca	**CFCC 56274**	***Euonymus japonicus***	**China**	**OQ344726**		**OQ398766**	**OQ410599**		–	–	–	–
Cytospora albodisca	**CFCC 57538**	***Euonymus japonicus***	**China**	**OQ344727**		**OQ398767**	**OQ410600**		–	–	–	–
Cytospora alba	CFCC 55462*	*Salix matsudana*	Gansu, China	MZ702593	OK303577	OK303644	OK303457	OK303516	–	–	–	–
Cytospora alba	CFCC 55463*	*Salix matsudana*	Gansu, China	MZ702594	OK303578	OK303645	OK303458	OK303517	–	–	–	–
Cytospora ampulliformis	MFLUCC 16-0583*	*Sorbus intermedia*	Russia	KY417726	–	–	KY417692	KY417794	–	–	–	–
Cytospora ampulliformis	MFLUCC 16-0629	*Acer platanoides*	Russia	KY417727	–	–	KY417693	KY417795	–	–	–	–

Cytospora amygdali	CBS 144233*	*Prunus dulcis*	California, USA	MG971853	MG971659	MG971718	MG972002	–	–	–	–	–
Cytospora atrocirrhata	CFCC 89615	*Juglans regia*	Qinghai, China	KR045618	KP310858	KR045659	KF498673	KU710946	–	–	–	–
Cytospora atrocirrhata	CFCC 89616	*Juglans regia*	Qinghai, China	KR045619	KP310859	KR045660	KF498674	KU710947	–	–	–	–
Cytospora atrocirrhata	CXY 1401	*Populus* sp.	Inner Mongolia, China	JX534242	–	KM034904	–	–	–	–	–	–
Cytospora atrocirrhata	CXY 1402	*Populus* sp.	Inner Mongolia, China	JX534243	–	KM034903	–	–	–	–	–	–
Cytospora beilinensis	CFCC 50493*	*Pinus armandii*	Beijing, China	MH933619	MH933495	MH933561	MH933527	–	–	–	–	–
Cytospora beilinensis	CFCC 50494	*Pinus armandii*	Beijing, China	MH933620	MH933496	MH933562	MH933528	–	–	–	–	–
Cytospora berberidis	CFCC 89927*	*Berberis dasystachya*	Qinghai, China	KR045620	KU710913	KR045661	KU710990	KU710948	–	–	–	–
Cytospora berberidis	CFCC 89933	*Berberis dasystachya*	Qinghai, China	KR045621	KU710914	KR045662	KU710991	KU710949	–	–	–	–
Cytospora bungeana	CFCC 50495*	*Pinus bungeana*	Shanxi, China	MH933621	MH933497	MH933563	MH933529	MH933593	–	–	–	–
Cytospora bungeana	CFCC 50496	*Pinus bungeana*	Shanxi, China	MH933622	MH933498	MH933564	MH933530	MH933594	–	–	–	–
Cytospora californica	CBS 144234*	*Juglans regia*	California, USA	MG971935	MG971645	–	MG972083	–	–	–	–	–
Cytospora carbonacea	CFCC 89947	*Ulmus pumila*	Qinghai, China	KR045622	KP310855	KP310825	KP310842	KU710950	–	–	–	–
Cytospora carpobroti	CMW 48981*	*Carpobrotus edulis*	South Africa	MH382812	MH411212	MH411207	–	–	–	–	–	–
Cytospora celtidicola	CFCC 50497*	*Celtis sinensis*	Anhui, China	MH933623	MH933499	MH933566	MH933531	MH933595	–	–	–	–
Cytospora celtidicola	CFCC 50498	*Celtis sinensis*	Anhui, China	MH933624	MH933500	MH933567	MH933532	MH933596	–	–	–	–
Cytospora centrivillosa	MFLUCC 16-1206*	*Sorbus domestica*	Italy	MF190122	–	–	–	MF377600	–	–	–	–
Cytospora centrivillosa	MFLUCC 17-1660	*Sorbus domestica*	Italy	MF190123	–	–	–	MF377601	–	–	–	–
Cytospora ceratosperma	CFCC 89624	*Juglans regia*	Gansu, China	KR045645	KP310860	KR045686	–	KU710976	–	–	–	–

Cytospora ceratosperma	CFCC 89625	*Juglans regia*	Gansu, China	KR045646	KP31086	KR045687	–	KU710977	–	–	–	–
Cytospora ceratospermopsis	CFCC 89626*	*Juglans regia*	Shaanxi, China	KR045647	KU710934	KR045688	KU711011	KU710978	–	–	–	–
Cytospora ceratospermopsis	CFCC 89627	*Juglans regia*	Shaanxi, China	KR045648	KU710935	KR045689	KU711012	KU710979	–	–	–	–
Cytospora chrysosperma	CFCC 89629	*Salix psammophila*	Shaanxi, China	KF765673	–	–	–	KF765705	–	–	–	–
Cytospora chrysosperma	CFCC 89981	*Populus alba* subsp. *pyramidalis*	Gansu, China	MH933625	MH933501	MH933568	MH933533	MH933597	– –	– –	– –	– –
Cytospora chrysosperma	CFCC 89982	*Ulmus pumila*	Tibet, China	KP281261	KP310848	KP310818	KP310835	–	–	–	–	–
Cytospora cinnamomea	CFCC 53178*	*Prunus armeniaca*	Xinjiang, China	MK673054	–	MK672970	MK673024	–	–	–	–	–
Cytospora coryli	CFCC 53162*	*Corylus mandshurica*	Beijing, China	MN854450	MN850758	MN861120	–	MN850751	–	–	–	–
Cytospora corylina	CFCC 54684*	*Corylus heterophylla*	Beijing, China	MW839861	MW815886	MW883969	MW815951	MW815937	–	–	–	–
Cytospora corylina	CFCC 54685	*Corylus heterophylla*	Beijing, China	MW839862	MW815887	MW883970	MW815952	MW815938	–	–	–	–
Cytospora corylina	CFCC 54686	*Corylus heterophylla*	Beijing, China	MW839863	MW815888	MW883971	MW815953	MW815939	–	–	–	–
Cytospora corylina	CFCC 54687	*Corylus heterophylla*	Beijing, China	MW839864	MW815889	MW883972	MW815954	MW815940	–	–	–	–
Cytospora cotini	MFLUCC 14-1050*	*Cotinus coggygria*	Russia	KX430142	–	–	–	KX430144	–	–	–	–
Cytospora cotoneastricola	CF 20197027	*Cotoneaster* sp.	Tibet, China	MK673072	MK672958	MK672988	MK673042	MK673012	–	–	–	–
Cytospora cotoneastricola	CF 20197028	*Cotoneaster* sp.	Tibet, China	MK673073	MK672959	MK672989	MK673043	MK673013	–	–	–	–
Cytospora cotoneastricola	CF 20197030	*Cotoneaster* sp.	Tibet, China	MK673074	MK672960	MK672990	MK673044	MK673014	–	–	–	–
Cytospora cotoneastricola	CF 20197031*	*Cotoneaster* sp.	Tibet, China	MK673075	MK672961	MK672991	MK673045	MK673015	–	–	–	–
Cytospora curvata	MFLUCC 15-0865*	*Salix alba*	Russia	KY417728	–	–	KY417694	KY417796	–	–	–	–

Cytospora curvispora	CFCC 54000*	*Corylus heterophylla*	Beijing, China	MW839851	MW815880	MW883963	MW815931	MW815945	–	–	–	–
Cytospora curvispora	CFCC 54001	*Corylus heterophylla*	Beijing, China	MW839853	MW815881	MW883964	MW815932	MW815946	–	–	–	–
Cytospora curvispora	CFCC 54676	*Corylus heterophylla*	Beijing, China	MW839854	MW815882	MW883965	MW815933	MW815947	–	–	–	–
Cytospora curvispora	CFCC 54677	*Corylus heterophylla*	Beijing, China	MW839855	MW815883	MW883966	MW815934	MW815948	–	–	–	–
Cytospora curvispora	CFCC 54678	*Corylus heterophylla*	Beijing, China	MW839856	MW815884	MW883967	MW815935	MW815949	–	–	–	–
Cytospora curvispora	CFCC 54679	*Corylus heterophylla*	Beijing, China	MW839857	MW815885	MW883968	MW815936	MW815950	–	–	–	–
Cytospora davidiana	CXY 1350*	*Populus davidiana*	Inner Mongolia,	KM034870	–	–	–	–	–	–	–	–
			China						–	–	–	–
Cytospora diopuiensis	CFCC 55884	*Kerria japonica* f. *pleniflora*	China	OK316819	OK358471	OK358473	–	OK358569	–	–	–	–
Cytospora diopuiensis	CFCC 55885	*Kerria japonica* f. *pleniflora*	China	OK316820	OK358472	OK358474	–	OK358470	–	–	–	–
Cytospora diopuiensis	MFLUCC 18-1419*	Undefined wood	Thailand	MK912137	–	–	MN685819	–	–	–	–	–
Cytospora diopuiensis	**CFCC 54692**	***Euonymus japonicus***	**China**	**OQ344752**	–	–	–	–	–	–	–	–
Cytospora diopuiensis	**CFCC 55479**	***Euonymus japonicus***	**China**	**OQ344753**	**OQ398762**	**OQ398791**	**OQ410625**	**OQ398735**	–	–	–	–
Cytospora diopuiensis	**CFCC 55527**	***Euonymus japonicus***	**China**	**OQ344754**	**OQ398763**	**OQ398792**	**OQ410626**	**OQ398736**	–	–	–	–
Cytospora diopuiensis	**CFCC 55528**	***Euonymus japonicus***	**China**	**OQ344755**	**OQ398764**	**OQ398793**	**OQ410627**	**OQ398737**	–	–	–	–
Cytospora discotoma	CFCC 53137 *	*Platycladus orientalis*	Beijing, China	MW418404	MW422919	MW422931	MW422897	MW422907	–	–	–	–
Cytospora discotoma	CFCC 54368	*Platycladus orientalis*	Beijing, China	MW418405	MW422920	MW422932	MW422898	MW422908	–	–	–	–
Cytospora discotoma	**CFCC 56276**	***Euonymus japonicus***	**China**	**OQ344728**	**OQ398739**	–	**OQ410601**	**OQ398713**	–	–	–	–
Cytospora donetzica	MFLUCC 15-0864	*Crataegus monogyna*	Russia	KY417729	–	–	KY417695	KY417797	–	–	–	–

Cytospora donetzica	MFLUCC 16-0574*	*Crataegus monogyna*	Russia	KY417731	–	–	KY417697	KY417799	–	–	–	–
Cytospora donglingensis	CFCC 53159 *	*Platycladus orientalis*	Beijing, China	MW418412	MW422927	MW422939	MW422903	MW422915	–	–	–	–
Cytospora donglingensis	CFCC 53160	*Platycladus orientalis*	Beijing, China	MW418414	MW422929	MW422941	MW422905	MW422917	–	–	–	–
Cytospora donglingensis	CFCC 54371	*Platycladus orientalis*	Beijing, China	MW418413	MW422928	MW422940	MW422904	MW422916	–	–	–	–
Cytospora donglingensis	CFCC 54372	*Platycladus orientalis*	Beijing, China	MW418415	MW422930	MW422942	MW422906	MW422918	–	–	–	–
Cytospora elaeagni	CFCC 89632	*Elaeagnus angustifolia*	Ningxia, China	KR045626	KU710918	KR045667	KU710995	KU710955	–	–	–	–
Cytospora elaeagni	CFCC 89633	*Elaeagnus angustifolia*	Ningxia, China	KF765677	KU710919	KR045668	KU710996	KU710956	–	–	–	–
Cytospora elaeagni	**CFCC 54082**	***Euonymus japonicus***	**China**	**OQ344729**	**OQ398740**	**OQ398768**	**OQ410602**	**OQ398714**	–	–	–	–
Cytospora elaeagni	**CFCC 55477**	***Euonymus japonicus***	**China**	**OQ344730**	**OQ398741**	**OQ398769**	**OQ410603**	**OQ398715**	–	–	–	–
Cytospora elaeagni	**CFCC 55526**	***Euonymus japonicus***	**China**	**OQ344731**	**OQ398742**	**OQ398770**	**OQ410604**	**OQ398716**	–	–	–	–
Cytospora elaeagni	**CFCC 56273**	***Euonymus japonicus***	**China**	**OQ344732**	**OQ398743**	**OQ398771**	**OQ410605**	**OQ398717**	–	–	–	–
Cytospora elaeagni	**CFCC 56287**	***Euonymus japonicus***	**China**	**OQ344733**	**OQ398744**	**OQ398772**	**OQ410606**	**OQ398718**	–	–	–	–
Cytospora elaeagnicola	CFCC 52882*	*Elaeagnus angustifolia*	China	MK732341	–	–	MK732344	MK732347	–	–	–	–
Cytospora elaeagnicola	CFCC 52883	*Elaeagnus angustifolia*	China	MK732342	–	–	MK732345	MK732348	–	–	–	–
Cytospora elaeagnicola	CFCC 52884	*Elaeagnus angustifolia*	China	MK732343	–	–	MK732346	MK732349	–	–	–	–
Cytospora erumpens	CFCC 50022	*Prunus padus*	Shanxi, China	MH933627	MH933502	MH933569	MH933534	–	–	–	–	–
Cytospora erumpens	MFLUCC 16-0580*	*Salix × fragilis*	Russia	KY417733	–	–	KY417699	KY417801	–	–	–	–
Cytospora erumpens	CFCC 53163	*Prunus padus*	Xinjiang, China	MK673059	MK672948	MK672975	MK673029	MK673000	–	–	–	–
Cytospora eucalypti	CBS 144241	*Eucalyptus globulus*	California, USA	MG971907	MG971617	MG971772	MG972056	–	–	–	–	–
Cytospora euonymicola	CFCC 50499*	*Euonymus kiautschovicus*	Shaanxi, China	MH933628	MH933503	MH933570	MH933535	MH933598	–	–	–	–
Cytospora euonymicola	CFCC 50500	*Euonymus kiautschovicus*	Shaanxi, China	MH933629	MH933504	MH933571	MH933536	MH933599	–	–	–	–

Cytospora euonymicola	**CFCC 54688**	***Euonymus japonicus***	**China**	**OQ344734**	**OQ398745**	**OQ398773**	**OQ410607**	**OQ398719**	–	–	–	–
Cytospora euonymicola	**CFCC 55530**	***Euonymus japonicus***	**China**	**OQ344735**	**OQ398746**	**OQ398774**	**OQ410608**	**OQ398720**	–	–	–	–
Cytospora euonymina	CFCC 89993*	*Euonymus kiautschovicus*	Shanxi, China	MH933630	MH933505	MH933590	MH933537	MH933600	–	–	–	–
Cytospora euonymina	CFCC 89999	*Euonymus kiautschovicus*	Shanxi, China	MH933631	MH933506	MH933591	MH933538	MH933601	–	–	–	–
Cytospora euonymina	**CFCC 55524**	***Euonymus japonicus***	**China**	**OQ344736**	**OQ398747**	**OQ398775**	**OQ410609**	–	–	–	–	–
Cytospora euonymina	**CFCC 55525**	***Euonymus japonicus***	**China**	**OQ344737**	**OQ398748**	**OQ398776**	**OQ410610**	**OQ398721**	–	–	–	–
Cytospora fraxinigena	BBH 42442	*Fraxinus ornus*	NA	MF190133	–	–	–	–	–	–	–	–
Cytospora fraxinigena	MFLUCC 14-0868*	*Fraxinus ornus*	Italy	MF190133	–	–	–	–	–	–	–	–
Cytospora fugax	CXY 1371	*Populus simonii*	Jilin, China	KM034852	–	KM034891	–	–	–	–	–	–
Cytospora fugax	CXY 1381	*Populus ussuriensis*	Heilongjiang, China	KM034853	–	KM034890	–	–	–	–	–	–
Cytospora fusispora	NFCCI 4372	NA	India	MN227694	–	–	–	–	–	–	–	–
Cytospora galegicola	MFLUCC 18-1199*	*Galega officinalis*	Forlì-Cesena, Italy	MK912128	–	–	MN685810	MN685820	–	–	–	–
Cytospora gigalocus	CFCC 89620*	*Juglans regia*	Qinghai, China	KR045628	KU710920	KR045669	KU710997	KU710957	–	–	–	–
Cytospora gigalocus	CFCC 89621	*Juglans regia*	Qinghai, China	KR045629	KU710921	KR045670	KU710998	KU710958	–	–	–	–
Cytospora gigaspora	CFCC 50014	*Juniperus procumbens*	Shanxi, China	KR045630	KU710922	KR045671	KU710999.	KU710959	–	–	–	–
Cytospora gigaspora	CFCC 89634*	*Salix psammophila*	Shaanxi, China	KF765671	KU710923	KR045672	KU711000	KU710960	–	–	–	–
Cytospora globosa	MFLU 16-2054*	*Abies alba*	Italy	MT177935	MT454016	–	–	MT432212	–	–	–	–
Cytospora granati	CBS 144237*	*Punica granatum*	California, USA	MG971799	MG971514	MG971664	MG971949	–	–	–	–	–
Cytospora haidianensis	CFCC 54056	*Euonymus alatus*	Beijing, China	MT360041	MT363997	MT364007	MT363978	MT363987	–	–	–	–
Cytospora haidianensis	CFCC 54057*	*Euonymus alatus*	Beijing, China	MT360042	MT363998	MT364008	MT363979	MT363988	–	–	–	–

Cytospora haidianensis	CFCC 54184	*Euonymus alatus*	Beijing, China	MT360043	MT363999	MT364009	MT363980	MT363989	–	–	–	–
Cytospora haidianensis	**CFCC 55480**	***Euonymus japonicus***	**China**	**OQ344738**	**OQ398749**	**OQ398777**	**OQ410611**	**OQ398722**	–	–	–	–
Cytospora haidianensis	**CFCC 55531**	***Euonymus japonicus***	**China**	**OQ344739**	**OQ398750**	**OQ398778**	**OQ410612**	**OQ398723**	–	–	–	–
Cytospora haidianensis	**CFCC 55532**	***Euonymus japonicus***	**China**	**OQ344740**	**OQ398751**	**OQ398779**	**OQ410613**	**OQ398724**	–	–	–	–
Cytospora haidianensis	**CFCC 55533**	***Euonymus japonicus***	**China**	**OQ344741**	**OQ398752**	**OQ398780**	**OQ410614**	**OQ398725**	–	–	–	–
Cytospora hippophaës	CFCC 89639	*Hippophaë rhamnoides*	Gansu, China	KR045632	KU710924	KR045673	KU711001	KU710961	–	–	–	–
Cytospora hippophaës	CFCC 89640	*Hippophaë rhamnoides*	Gansu, China	KF765682	KP310865	KR045674	KF765730	KU710962	–	–	–	–
Cytospora japonica	CFCC 89956	*Prunus cerasifera*	Ningxia, China	KR045624	KU710916	KR045665	KU710993	KU710953	–	–	–	–
Cytospora japonica	CFCC 89960	*Prunus cerasifera*	Ningxia, China	KR045625	KU710917	KR045666	KU710994	KU710954	–	–	–	–
Cytospora joaquinensis	CBS 144235	*Populus deltoides*	California, USA	MG971895	MG971605	MG971761	MG972044	–	–	–	–	–
Cytospora junipericola	BBH 42444	*Juniperus communis*	Italy	MF190126	MF377579	–	–	–	–	–	–	–
Cytospora junipericola	MFLU 17-0882*	*Juniperus communis*	Italy	MF190125	MF377580	–	–	–	–	–	–	–
Cytospora juniperina	CFCC 50501*	*Juniperus przewalskii*	Sichuan, China	MH933632	MH933507	–	MH933539	MH933602	–	–	–	–
Cytospora juniperina	CFCC 50502	*Juniperus przewalskii*	Sichuan, China	MH933633	MH933508	MH933572	MH933540	MH933603	–	–	–	–
Cytospora juniperina	CFCC 50503	*Juniperus przewalskii*	Sichuan, China	MH933634	MH933509	–	MH933541	MH933604	–	–	–	–
Cytospora kantschavelii	CXY 1383	*Populus maximowiczii*	Jilin, China	KM034867	–	–	–	–	–	–	–	–
Cytospora kantschavelii	CXY 1386	*Populus maximowiczii*	Chongqing, China	KM034867	–	–	–	–	–	–	–	–
Cytospora kuanchengensis	CFCC 52464*	*Castanea mollissima*	China	MK432616	–	–	MK442940	MK578076	–	–	–	–
Cytospora kuanchengensis	CFCC 52465	*Castanea mollissima*	China	MK432617	–	–	MK442941	MK578077	–	–	–	–
Cytospora longispora	CBS 144236*	*Prunus domestica*	California, USA	MG971905	MG971615	MG971764	MG972054	–	–	–	–	–
Cytospora longistiolata	MFLUCC 16-0628	*Salix × fragilis*	Russia	KY417734	–	–	KY417700	KY417802	–	–	–	–

Cytospora leucosperma	CFCC 89622	*Pyrus bretschneideri*	Gansu, China	KR045616	KU710911	KR045657	KU710988	KU710944	–	–	–	–
Cytospora leucosperma	CFCC 89894	*Pyrus bretschneideri*	Qinghai, China	KR045617	KU710912	KR045658	KU710989	KU710945	–	–	–	–
Cytospora leucostoma	CFCC 50023	*Cornus alba*	Shanxi, China	KR045635	KU710926	KR045676	KU711003	KU710964	–	–	–	–
Cytospora leucostoma	CFCC 50024	*Prunus pseudocerasus*	Qinghai, China	MH933640	–	MH933576	MH933547	MH933605	–	–	–	–
Cytospora leucostoma	CFCC 53140	*Prunus sibirica*	Beijing, China	MN854445	MN850753	MN861115	MN850760	MN850746	–	–	–	–
Cytospora leucostoma	CFCC 53141	*Prunus sibirica*	Beijing, China	MN854446	MN850754	MN861116	MN850761	MN850747	–	–	–	–
Cytospora leucostoma	CFCC 53156	*Juglans mandshurica*	Beijing, China	MN854447	MN850755	MN861117	MN850762	MN850748	–	–	–	–
Cytospora leucostoma	CFCC 53167	*Prunus armeniaca*	Xinjiang, China	MK673056	MK672946	MK672972	MK673026	MK672998	–	–	–	–
Cytospora leucostoma	CFCC 53169	*Prunus persica*	Beijing, China	MK673080	MK672966	MK672996	MK673050	MK673020	–	–	–	–
Cytospora leucostoma	CFCC 53170	*Prunus persica*	Beijing, China	MK673081	MK672967	MK672997	MK673051	MK673021	–	–	–	–
Cytospora leucostoma	CFCC 54680	*Corylus heterophylla*	Beijing, China	MW839857	MW815890	MW883973	MW815941	MW815955	–	–	–	–
Cytospora leucostoma	CFCC 54681	*Corylus heterophylla*	Beijing, China	MW839857	MW815891	MW883974	MW815942	MW815956	–	–	–	–
Cytospora leucostoma	CFCC 54682	*Corylus heterophylla*	Beijing, China	MW839857	MW815892	MW883975	MW815943	MW815957	–	–	–	–
Cytospora leucostoma	CFCC 54683	*Corylus heterophylla*	Beijing, China	MW839857	MW815893	MW883976	MW815944	MW815958	–	–	–	–
Cytospora leucostoma	**CFCC 55474**	***Euonymus japonicus***	**China**	**OQ344742**	**OQ398753**	**OQ398781**	**OQ410615**	**OQ398726**	–	–	–	–
Cytospora leucostoma	**CFCC 55475**	***Euonymus japonicus***	**China**	**OQ344743**	**OQ398754**	**OQ398782**	**OQ410616**	**OQ398727**	–	–	–	–
Cytospora leucostoma	**CFCC 55476**	***Euonymus japonicus***	**China**	**OQ344744**	**OQ398755**	**OQ398783**	**OQ410617**	–	–	–	–	–
Cytospora leucostoma	**CFCC 55478**	***Euonymus japonicus***	**China**	**OQ344745**	**OQ398756**	**OQ398784**	**OQ410618**	**OQ398728**	–	–	–	–
Cytospora leucostoma	**CFCC 55519**	***Euonymus japonicus***	**China**	**OQ344746**	**OQ398757**	**OQ398785**	**OQ410619**	**OQ398729**	–	–	–	–
Cytospora leucostoma	**CFCC 55520**	***Euonymus japonicus***	**China**	**OQ344747**	**OQ398758**	**OQ398786**	**OQ410620**	**OQ398730**	–	–	–	–

Cytospora leucostoma	**CFCC 55521**	***Euonymus japonicus***	**China**	**OQ344748**	–	**OQ398787**	**OQ410621**	**OQ398731**	–	–	–	–
Cytospora lumnitzericola	MFLUCC 17-0508*	*Lumnitzera racernosa*	Tailand	MG975778	–	–	MH253457	MH253453	–	–	–	–
Cytospora mali	CFCC 50028	*Malus pumila*	Gansu, China	MH933641	MH933513	MH933577	MH933548	MH933606	–	–	–	–
Cytospora mali	CFCC 50029	*Malus pumila*	Ningxia, China	MH933642	MH933514	MH933578	MH933549	MH933607	–	–	–	–
Cytospora mali	CFCC 50030	*Malus pumila*	Shaanxi, China	MH933643	MH933524	MH933579	MH933550	MH933608	–	–	–	–
Cytospora mali	CFCC 50031	*Crataegus* sp.	Shanxi, China	KR045636	KU710927	KR045677	KU711004	KU710965	–	–	–	–
Cytospora mali	CFCC 50044	*Malus baccata*	Qinghai, China	KR045637	KU710928	KR045678	KU711005	KU710966	–	–	–	–
Cytospora mali-spectabilis	CFCC 53181*	*Malus spectabilis* 'Royalty'	Xinjiang, China	MK673066	MK672953	MK672982	MK673036	MK673006	–	–	–	–
Cytospora melnikii	CFCC 89984	*Rhus typhina*	Xinjiang, China	MH933678	MH933515	MH933580	MH933551	MH933609	–	–	–	–
Cytospora melnikii	MFLUCC 15-0851	*Malus domestica*	Russia	KY417735	–	–	KY417701	KY417803	–	–	–	–
Cytospora melnikii	MFLUCC 16-0635	*Populus nigra* var. *italica*	Russia	KY417736	–	–	KY417702	KY417804	–	–	–	–
Cytospora myrtagena	CFCC 52454	*Castanea mollissima*	China	MK432614	–	–	MK442938	MK578074	–	–	–	–
Cytospora myrtagena	CFCC 52455	*Castanea mollissima*	China	MK432615	–	–	MK442939	MK578075	–	–	–	–
Cytospora nivea	MFLUCC 15-0860	*Salix acutifolia*	Russia	KY417737	–	–	KY417703	KY417805	–	–	–	–
Cytospora nivea	CFCC 89641	*Elaeagnus angustifolia*	Ningxia, China	KF765683	KU710929	KR045679	KU711006	KU710967	–	–	–	–
Cytospora nivea	CFCC 89643	*Salix psammophila*	Shaanxi, China	KF765685	KP310863	KP310829	–	KU710968	–	–	–	–
Cytospora notastroma	NE_*FR5	*Populus tremuloides*	USA	JX438632	JX438543	–	–	–	–	–	–	–
Cytospora notastroma	NE_*FR8	*Populus tremuloides*	USA	JX438633	JX438542	–	–	–	–	–	–	–
Cytospora ochracea	CFCC 53164*	*Cotoneaster* sp.	Xinjiang, China	MK673060	MK672949	MK672976	MK673030	MK673001	–	–	–	–
Cytospora oleicola	CBS 144248*	*Olea europaea*	California, USA	MG971944	MG971660	MG971752	MG972098	–	–	–	–	–
Cytospora olivacea	CFCC 53174	*Prunus cerasifera*	Xinjiang, China	MK673058	–	MK672974	MK673028	MK672999	–	–	–	–

Cytospora olivacea	CFCC 53175	*Prunus dulcis*	Xinjiang, China	MK673062	–	MK672978	MK673032	MK673003	–	–	–	–
Cytospora olivacea	CFCC 53176*	*Sorbus tianschanica*	Xinjiang, China	MK673068	MK672955	MK672984	MK673038	MK673008	–	–	–	–
Cytospora olivacea	CFCC 53177	*Prunus virginiana*	Xinjiang, China	MK673071	–	MK672987	MK673041	MK673011	–	–	–	–
Cytospora palm	CXY 1276	*Cotinus coggygria*	Beijing, China	JN402990	KJ781296	–	–	–	–	–	–	–
Cytospora palm	CXY 1280*	*Cotinus coggygria*	Beijing, China	JN411939	KJ781297	–	–	–	–	–	–	–
Cytospora paracinnamomea	CFCC 55453*	*Salix matsudana*	Gansu, China	MZ702594	OK303576	OK303643	OK303456	OK303515	–	–	–	–
Cytospora paracinnamomea	CFCC 55455*	*Salix matsudana*	Gansu, China	MZ702598	OK303580	OK303647	OK303460	OK303519	–	–	–	–
Cytospora parakantschavelii	MFLUCC 15-0857*	*Populus × sibirica*	Russia	KY417738	–	–	KY417704	KY417806	–	–	–	–
Cytospora parakantschavelii	MFLUCC 16-0575	*Pyrus pyraster*	Russia	KY417739	–	–	KY417705	KY417807	–	–	–	–
Cytospora parapistaciae	CBS 144506*	*Pistacia vera*	California, USA	MG971804	MG971519	MG971669	MG971954	–	–	–	–	–
Cytospora parasitica	MFLUCC 15-0507*	*Malus domestica*	Russia	KY417740	–	–	KY417706	KY417808	–	–	–	–
Cytospora parasitica	XJAU 2542-1	*Malus* sp.	Xinjiang, China	MH798884	MH813452	–	–	–	–	–	–	–
Cytospora parasitica	CFCC 53171	*Malus pumila*	Xinjiang, China	MK673061	MK672950	MK672977	MK673031	MK673002	–	–	–	–
Cytospora parasitica	CFCC 53172	*Malus pumila*	Xinjiang, China	MK673069	MK672956	MK672985	MK673039	MK673009	–	–	–	–
Cytospora parasitica	CFCC 53173	*Berberis* sp.	Xinjiang, China	MK673070	MK672957	MK672986	MK673040	MK673010	–	–	–	–
Cytospora paratranslucens	MFLUCC 15-0506*	*Populus alba* var. *bolleana*	Russia	KY417741	–	–	KY417707	KY417809	–	–	–	–
Cytospora paratranslucens	MFLUCC 16-0627	*Populus alba*	Russia	KY417742	–	–	KY417708	KY417810	–	–	–	–
Cytospora phialidica	MFLUCC 17-2498	*Alnus glutinosa*	Italy	MT177932	MT454014	–	–	MT432209	–	–	–	–

Cytospora piceae	CFCC 52841**	*Picea crassifolia*	Xinjiang, China	MH820398	MH820402	MH820387	MH820406	MH820395	–	–	–	–
Cytospora piceae	CFCC 52842*	*Picea crassifolia*	Xinjiang, China	MH820399	MH820403	MH820388	MH820407	MH820396	–	–	–	–
Cytospora pingbianensis	MFLUCC 18-1204*	Undefined wood	Yunnan, China	MK912135	–	–	MN685817	MN685826	–	–	–	–
Cytospora pistaciae	CBS 144238*	*Pistacia vera*	California, USA	MG971802	MG971517	MG971667	MG971952	–	–	–	–	–
Cytospora platanicola	MFLU 17-0327	*Platanus hybrida*	Italy	MH253451	–	–	MH253449	MH253450	–	–	–	–
Cytospora platycladi	CFCC 50504**	*Platycladus orientalis*	Yunnan, China	MH933645	MH933516	MH933581	MH933552	MH933610	–	–	–	–
Cytospora platycladi	CFCC 50505*	*Platycladus orientalis*	Yunnan, China	MH933646	MH933517	MH933582	MH933553	MH933611	–	–	–	–
Cytospora platycladi	CFCC 50506*	*Platycladus orientalis*	Yunnan, China	MH933647	MH933518	MH933583	MH933554	MH933612	–	–	–	–
Cytospora platycladicola	CFCC 50038**	*Platycladus orientalis*	Gansu, China	KT222840	MH933519	MH933584	MH933555	MH933613	–	–	–	–
Cytospora platycladicola	CFCC 50039*	*Platycladus orientalis*	Gansu, China	KR045642	KU710931	KR045683	KU711008	KU710973	–	–	–	–
Cytospora plurivora	CBS 144239*	*Olea europaea*	California, USA	MG971861	MG971572	MG971726	MG972010	–	–	–	–	–
Cytospora populicola	CBS 144240	*Populus deltoides*	California, USA	MG971891	MG971601	MG971757	MG972040	–	–	–	–	–
Cytospora populina	CFCC 89644*	*Salix psammophila*	Shaanxi, China	KF765686	KU710930	KR045681	KU711007	KU710969	–	–	–	–
Cytospora populinopsis	CFCC 50032*	*Sorbus aucuparia*	Ningxia, China	MH933648	MH933520	MH933585	MH933556	MH933614	–	–	–	–
Cytospora populinopsis	CFCC 50033	*Sorbus aucuparia*	Ningxia, China	MH933649	MH933521	MH933586	MH933557	MH933615	–	–	–	–
Cytospora predappioensis	MFLUCC 17-2458*	*Platanus hybrida*	Italy	MG873484	–	–	–	–	–	–	–	–
Cytospora prunicola	MFLU 17-0995*	*Prunus* sp.	Italy	MG742350	–	–	MG742353	MG742352	–	–	–	–
Cytospora pruni-mume	CFCC 53179	*Prunus armeniaca*	Xinjiang, China	MK673057	MK672947	MK672973	MK673027	–	–	–	–	–
Cytospora pruni-mume	CFCC 53180*	*Prunus mume*	Xinjiang, China	MK673067	MK672954	MK672983	MK673037	MK673007	–	–	–	–

Cytospora pruinopsis	CFCC 50034*	*Ulmus pumila*	Shaanxi, China	KP281259	KP310849	KP310819	KP310836	KU710970	–	–	–	–
Cytospora pruinopsis	CFCC 50035	*Ulmus pumila*	Jilin, China	KP281260	KP310850	KP310820	KP310837	KU710971	–	–	–	–
Cytospora pruinopsis	CFCC 53153	*Ulmus pumila*	Beijing, China	MN854451	MN850759	MN861121	MN850763	MN850752	–	–	–	–
Cytospora pruinosa	CFCC 50036	*Syringa oblata*	Qinghai, China	KP310800	KP310845	KP310815	KP310832	–	–	–	–	–
Cytospora pruinosa	CFCC 50037	*Syringa oblata*	Qinghai, China	MH933650	MH933522	MH933589	MH933558	–	–	–	–	–
Cytospora pubescentis	MFLUCC 18-1201*	*Quercus pubescens*	Forlì-Cesena, Italy	MK912130	–	–	MN685812	MN685821	–	–	–	–
Cytospora punicae	CBS 144244	*Punica granatum*	California, USA	MG971943	MG971654	MG971798	MG972091	–	–	–	–	–
Cytospora quercicola	MFLU 17-0881	*Quercus* sp.	Italy	MF190128	–	–	–	–	–	–	–	–
Cytospora quercicola	MFLUCC 14-0867*	*Quercus* sp.	Italy	MF190129	–	–	–	–	–	–	–	–
Cytospora ribis	CFCC 50026	*Ulmus pumila*	Qinghai, China	KP281267	KP310856	KP310826	KP310843	KU710972	–	–	–	–
Cytospora ribis	CFCC 50027	*Ulmus pumila*	Qinghai, China	KP281268	KP310857	KP310827	KP310844	–	–	–	–	–
Cytospora rosae	MFLU 17-0885	*Rosa canina*	Italy	MF190131	–	–	–	–	–	–	–	–
Cytospora rosicola	CF 20197024*	*Rosa* sp.	Tibet, China	MK673079	MK672965	MK672995	MK673049	MK673019	–	–	–	–
Cytospora rosigena	MFLUCC 18-0921*	*Rosa* sp.	Russia	MN879872	–	–	–	–	–	–	–	–
Cytospora rostrata	CFCC 89909	*Salix cupularis*	Gansu, China	KR045643	KU710932	KR045684	KU711009	KU710974	–	–	–	–
Cytospora rostrata	CFCC 89910	*Salix cupularis*	Gansu, China	KR045644	KU710933	–	KU711010	KU710975	–	–	–	–
Cytospora rusanovii	MFLUCC 15-0853	*Populus × sibirica*	Russia	KY417743	–	–	KY417709	KY417811	–	–	–	–
Cytospora rusanovii	MFLUCC 15-0854*	*Salix babylonica*	Russia	KY417744	–	–	KY417710	KY417812	–	–	–	–
Cytospora salicacearum	MFLUCC 15-0509	*Salix alba*	Russia	KY417746	–	–	KY417712	KY417814	–	–	–	–
Cytospora salicacearum	MFLUCC 15-0861	*Salix × fragilis*	Russia	KY417745	–	–	KY417711	KY417813	–	–	–	–

Cytospora salicacearum	MFLUCC 16-0587	*Prunus cerasus*	Russia	KY417742	–	–	KY417708	KY417810	–	–	–	–
Cytospora salicacearum	MFLUCC 16-0576	*Populus nigra* var. *italica*	Russia	KY417741	–	–	KY417707	KY417809	–	–	–	–
Cytospora salicicola	MFLUCC 14-1052*	*Salix alba*	Russia	KU982636	–	–	KU982637	–	–	–	–	–
Cytospora salicicola	MFLUCC 15-0866	*Salix* sp.	Thailand	KY417749	–	–	KY417715	KY417817	–	–	–	–
Cytospora salicina	MFLUCC 15-0862	*Salix alba*	Russia	KY417750	–	–	KY417716	KY417818	–	–	–	–
Cytospora salicina	MFLUCC 16-0637	*Salix × fragilis*	Russia	KY417751	–	–	KY417717	KY417819	–	–	–	–
Cytospora schulzeri	CFCC 50042	*Malus pumila*	Gansu, China	KR045650	KU710937	KR045691	KU711014	KU710981	–	–	–	–
Cytospora sibiraeae	CFCC 50045*	*Sibiraea angustata*	Gansu, China	KR045651	KU710938	KR045692	KU711015	KU710982	–	–	–	–
Cytospora sibiraeae	CFCC 50046	*Sibiraea angustata*	Gansu, China	KR045652	KU710939	KR045693	KU711015	KU710983	–	–	–	–
Cytospora sophorae	CFCC 50047	*Styphnolobium japonicum*	Shanxi, China	KR045653	KU710940	KR045694	KU711017	KU710984	–	–	–	–
Cytospora sophorae	CFCC 50048	*Magnolia grandiflora*	Shanxi, China	MH820401	MH820405	MH820390	MH820409	MH820397	–	–	–	–
Cytospora sophorae	CFCC 89598	*Styphnolobium japonicum*	Gansu, China	KR045654	KU710941	KR045695	KU711018	KU710985	–	–	–	–
Cytospora sophorae	**CFCC 55523**	***Euonymus japonicus***	**China**	**OQ344749**	**OQ398759**	**OQ398788**	**OQ410622**	**OQ398732**	–	–	–	–
Cytospora sophoricola	CFCC 89596	*Styphnolobium japonicum var. pendula*	Gansu, China	KR045656	KU710943	KR045697	KU711020	KU710987	–	–	–	–
Cytospora sophoricola	CFCC 89595*	*Styphnolobium japonicum var. pendula*	Gansu, China	KR045655	KU710942	KR045696	KU711019	KU710986	–	–	–	–
Cytospora sophoriopsis	CFCC 55469	*Salix matsudana*	Gansu, China	MZ702583	OK303565	OK303632	OK303445	OK303504	–	–	–	–
Cytospora sophoriopsis	CFCC 89600	*Styphnolobium japonicum*	Gansu, China	KR045623	KU710915	KP310817	KU710992	KU710951	–	–	–	–
Cytospora sorbi	MFLUCC 16-0631*	*Sorbus aucuparia*	Russia	KY417752	–	–	KY417718	KY417820	–	–	–	–

Cytospora sorbicola	MFLUCC 16-0584*	*Acer pseudoplatanus*	Russia	KY417755	–	–	KY417721	KY417823	–	–	–	–
Cytospora sorbicola	MFLUCC 16-0633	*Cotoneaster melanocarpus*	Russia	KY417758	–	–	KY417724	KY417826	–	–	–	–
Cytospora sorbina	CF 20197660*	*Sorbus tianschanica*	Xinjiang, China	MK673052	MK672943	MK672968	MK673022	–	–	–	–	–
Cytospora spiraeae	CFCC 50049*	*Spiraea salicifolia*	Gansu, China	MG707859	–	–	MG708196	MG708199	–	–	–	–
Cytospora spiraeae	CFCC 50050	*Spiraea salicifolia*	Gansu, China	MG707860	–	–	MG708197	MG708200	–	–	–	–
Cytospora spiraeicola	CFCC 53138*	*Spiraea salicifolia*	Beijing, China	MN854448	MN850756	MN861118	–	MN850749	–	–	–	–
Cytospora spiraeicola	CFCC 53139	*Tilia nobilis*	Beijing, China	MN854449	MN850757	MN861119	–	MN850750	–	–	–	–
Cytospora tamaricicola	CFCC 50507	*Rosa multifolora*	Yunnan, China	MH933651	MH933525	MH933587	MH933559	MH933616	–	–	–	–
Cytospora tamaricicola	CFCC 50508*	*Tamarix chinensis*	Yunnan, China	MH933652	MH933523	MH933588	MH933560	MH933617	–	–	–	–
Cytospora tanaitica	MFLUCC 14-1057*	*Betula pubescens*	Russia	KT459411	–	–	KT459413	–	–	–	–	–
Cytospora thailandica	MFLUCC 17-0262*	*Xylocarpus moluccensis*	Thailand	MG975776	–	–	MH253459	MH253455	–	–	–	–
Cytospora thailandica	MFLUCC 17-0263*	*Xylocarpus moluccensis*	Thailand	MG975777	–	–	MH253460	MH253456	–	–	–	–
Cytospora tibetensis	CF 20197026	*Cotoneaster* sp.	Tibet, China	MK673076	MK672962	MK672992	MK673046	MK673016	–	–	–	–
Cytospora tibetensis	CF 20197029	*Cotoneaster* sp.	Tibet, China	MK673077	MK672963	MK672993	MK673047	MK673017	–	–	–	–
Cytospora tibetensis	CF 20197032*	*Cotoneaster* sp.	Tibet, China	MK673078	MK672964	MK672994	MK673048	MK673018	–	–	–	–
Cytospora tibouchinae	CPC 26333*	*Tibouchina semidecandra*	France	KX228284	–	–	–	–	–	–	–	–
Cytospora translucens	CXY 1351	*Populus davidiana*	Inner Mongolia, China	KM034874	–	KM034895	–	–	–	–	–	–
Cytospora translucens	CXY 1359	*Populus* × Beijingensis	Beijing, China	KM034871	–	KM034894	–	–	–	–	–	–
Cytospora ulmi	MFLUCC 15-0863*	*Ulmus minor*	Russia	KY417759	–	–	–	–	–	–	–	–

Cytospora verrucosa	CFCC 53157 *	*Platycladus orientalis*	Beijing, China	MW418408	MW422923	MW422935	–	MW422911	–	–	–	–
Cytospora verrucosa	CFCC 53158	*Platycladus orientalis*	Beijing, China	MW418410	MW422925	MW422937	MW422901	MW422913	–	–	–	–
Cytospora verrucosa	CFCC 54369	*Platycladus orientalis*	Beijing, China	MW418409	MW422924	MW422936	–	MW422912	–	–	–	–
Cytospora verrucosa	CFCC 54370	*Platycladus orientalis*	Beijing, China	MW418411	MW422926	MW422938	MW422902	MW422914	–	–	–	–
Cytospora vinacea	CBS 141585*	*Vitis interspecific hybrid 'Vidal'*	USA	KX256256	KX256277	KX256235	–	–	–	–	–	–
Cytospora viridistroma	CBS 202.36*	*Cercis canadensis* Castigl.	USA	MN172408	MN271853	–	–	–	–	–	–	–
Cytospora viticola	Cyt2	*Vitis interspecific hybrid 'Frontenac'*	USA	KX256238	KX256259	KX256217	–	–	–	–	–	–
Cytospora viticola	CBS 141586*	*Vitis vinifera 'CabernetFranc'*	USA	KX256239	KX256260	KX256218	–	–	–	–	–	–
Cytospora xinjiangensis	CFCC 53182	*Rosa* sp.	Xinjiang, China	MK673064	MK672951	MK672980	MK673034	MK673004	–	–	–	–
Cytospora xinjiangensis	CFCC 53183*	*Rosa* sp.	Xinjiang, China	MK673065	MK672952	MK672981	MK673035	MK673005	–	–	–	–
Cytospora xinglongensis	CFCC 52458	*Castanea mollissima*	China	MK432622	–	–	MK442946	MK578082	–	–	–	–
Cytospora xinglongensis	CFCC 52459	*Castanea mollissima*	China	MK432623	–	–	MK442947	MK578083	–	–	–	–
Cytospora xylocarpi	MFLUCC 17-0251*	*Xylocarpus granatum*	Thailand	MG975775	–	–	MH253458	MH253454	–	–	–	–
Cytospora zhaitangensis	**CFCC 56227***	***Euonymus japonicus***	**China**	**OQ344750**	**OQ398760**	**OQ398789**	**OQ410623**	**OQ398733**	–	–	–	–
Cytospora zhaitangensis	**CFCC 57537**	***Euonymus japonicus***	**China**	**OQ344751**	**OQ398761**	**OQ398790**	**OQ410624**	**OQ398734**	–	–	–	–
Diaporthe vaccinii	CBS 160.32	*Vaccinium macrocarpon*	USA	KC343228	KC343954	KC344196	JQ807297	–	–	–	–	–
Diaporthe absenteum	LC 3924*	*Camellia sinensis*	China	KP267897	KP267971	KP293477	–	–	–	–	–	KP293547
Diaporthe acaciigena	CBS 129521*	*Acacia retinodes*	Australia	KC343005	KC343731	KC343973	–	–	–	–	KC343247	KC343489
Diaporthe acericola	MFLUCC 17-0956*	*Acer negundo*	Italy	KY964224	KY964180	KY964074	–	–	–	–	KY964137	–

Diaporthe acerigena	CFCC 52554*	*Acer tataricum*	China	MH121489	MH121531	–	–	–	–	–	MH121413	MH121449
Diaporthe acerigena	CFCC 52555	*Acer tataricum*	China	MH121490	MH121532	–	–	–	–	–	MH121414	MH121450
Diaporthe acerina	CBS 137.27	*Acer negundo*	NA	KC343006	KC343732	KC343974	–	–	–	–	KC343248	KC343490
Diaporthe actinidiae	ICMP 13683*	*Actinidia deliciosa*	New Zealand	KC145886	KC145941	–	–	–	–	–	–	–
Diaporthe acuta	PSCG 047*	*Pyrus pyrifolia*	China	MK626957	MK654802	MK691225	–	–	–	–	MK691125	MK726161
Diaporthe acutispora	LC6161*	*Coffea* sp.	China	KX986764	KX999155	KX999195	–	–	–	–	KX999274	KX999235
Diaporthe alangii	CFCC 52556*	*Alangium kurzii*	China	MH121491	MH121533	MH121573	–	–	–	–	MH121415	MH121451
Diaporthe alangii	CFCC 52557	*Alangium kurzii*	China	MH121492	MH121534	MH121574	–	–	–	–	MH121416	MH121452
Diaporthe albosinensis	CFCC 53066	*Betula albosinensis*	China	MK432659	MK578133	MK578059	–	–	–	–	MK442979	MK443004
Diaporthe albosinensis	CFCC 53067	*Betula albosinensis*	China	MK432660	MK578134	MK578060	–	–	–	–	MK442980	MK443005
Diaporthe alleghaniensis	CBS 495.72*	*Betula alleghaniensis*	Canada	MH121502	MH121544	MH121584	–	–	–	–	MH121426	MH121462
Diaporthe alnea	CBS 146.46*	*Alnus* sp.	Netherlands	KC343008	KC343734	KC343976	–	–	–	–	KC343250	KC343492
Diaporthe amaranthophila	MAFF 246900	*Amaranthus tricolor*	Japan	LC459575	LC459577	LC459579	–	–	–	–	LC459583	LC459581
Diaporthe ambigua	CBS 114015	*Pyrus communis*	South Africa	KC343010	KC343736	KC343978	–	–	–	–	KC343252	KC343494
Diaporthe ampelina	STE-U 2660	*Vitis vinifera*	France	–	AY745056	–	–	–	–	–	AY745026	–
Diaporthe amygdali	CBS 126679*	*Prunus dulcis*	Portugal	MH864208	KC343748	KC343990	–	–	–	–	KC343264	KC343506
Diaporthe anacardii	CBS 720.97*	*Anacardium occidentale*	East Africa	KC343024	KC343750	KC343992	–	–	–	–	KC343266	KC343508
Diaporthe angelicae	CBS 111592*	*Heracleum sphondylium*	Austria	KC343027	KC343753	KC343995	–	–	–	–	KC343269	KC343511
Diaporthe anhuiensis	CNUCC 201901*	*Cunninghamia lanceolata*	China	MN219718	MN224668	MN227008	–<br>–	–<br>–	–<br>–	–<br>–	MN224549	MN224556
Diaporthe apiculatum	CFCC 53068	*Rhus chinensis*	China	MK432651	MK578127	MK578054	–	–	–	–	MK442973	MK442998
Diaporthe apiculatum	CFCC 53069	*Rhus chinensis*	China	MK432652	MK578128	MK578055	–	–	–	–	MK44297	MK442999
Diaporthe aquatica	IFRDCC 3051*	*Aquatic habitat*	China	JQ797437	–	–	–	–	–	–	–	–
Diaporthe araucanorum	CBS 145285*	*Araucaria araucana*	Chile	MN509711	MN509733	MN509722	–	–	–	–	MN974277	–
Diaporthe araucanorum	CBS 145286	*Araucaria araucana*	Chile	MN509712	MN509734	MN509723	–	–	–	–	–	–

Diaporthe arctii	DP0482*	*Arctium lappa*	Austria	KJ590736	KJ590776	KJ610891	–	–	–	–	KJ612133	KJ659218
Diaporthe arecae	CBS 161.64*	*Areca catechu*	India	KC343032	KC343758	KC344000	–	–	–	–	KC343274	KC343516
Diaporthe arengae	CBS 114979*	*Arenga engleri*	Hong Kong	MF773664	KC343760	KC344002	–	–	–	–	KC343276	KC343518
Diaporthe arezzoensis	MFLU 19-2880*	MFLU 19-2880T	*Cytisus sp.*	Italy	–	MT454019	–	–	–	–	MT185503	–
Diaporthe aseana	MFLUCC 12-0299a	Unknown	Thailand	KT459414	KT459448	KT459432	–	–	–	–	KT459464	–
Diaporthe asheicola	CBS 136967	*Vaccinium ashei*	Chile	KJ160562	KJ160594	KJ160518	–	–	–	–	KJ160542	–
Diaporthe aspalathi	CBS 117169*	*Aspalathus linearis*	South Africa	KC343036	KC343762	KC344004	–	–	–	–	KC343278	KC343520
Diaporthe australafricana	CBS 111886*	*Vitis vinifera*	Australia	KC343038	KC343764	KC344006	–	–	–	–	KC343280	KC343522
Diaporthe australiana	BRIP 66145*	*Macadamia* sp.	Australia	MN708222	MN696522	MN696530	–	–	–	–	–	–
Diaporthe baccae	CBS 136972*	*Vaccinium corymbosum*	Italy	MK370623	KJ160597	MF418509	–	–	–	–	MG281695	MF418264
Diaporthe batatas	CBS 122.21*	*Ipomoea batatas*	USA	KC343040	KC343766	KC344008	–	–	–	–	KC343282	KC343524
Diaporthe bauhiniae	CFCC 53071	*Bauhinia purpurea*	China	MK432648	MK578124	MK578051	–	–	–	–	MK442970	MK442995
Diaporthe bauhiniae	CFCC 53072	*Bauhinia purpurea*	China	MK432649	MK578125	MK578052	–	–	–	–	MK442971	MK442996
Diaporthe bauhiniae	CFCC 53073	*Bauhinia purpurea*	China	MK432650	MK578126	MK578053	–	–	–	–	MK442972	MK442997
Diaporthe beilharziae	BRIP 54792*	*Indigofera australis*	Australia	JX862529	JX862535	KF170921	–	–	–	–	–	–
Diaporthe benedicti	SBen914	*Diaporthe benedicti*	USA	KM669929	KM669785	–	–	–	–	–	KM669862	–
Diaporthe betulae	CFCC 50469	*Betula platyphylla*	China	KT732950	KT733016	KT733020	–	–	–	–	KT732997	KT732999
Diaporthe betulae	CFCC 50470	*Betula platyphylla*	China	KT732951	KT733017	KT733021	–	–	–	–	KT732998	KT733000
Diaporthe betulicola	CFCC 51128*	*Betula albo-sinensis*	China	KX024653	KX024655	KX024657	–	–	–	–	KX024659	KX024661
Diaporthe betulicola	CFCC 51129	*Betula albo-sinensis*	China	KX0246554	KX0246556	KX024658	–	–	–	–	KX024660	KX024662
Diaporthe betulina	CFCC 52560	*Betula albo-sinensis*	China	MH121495	MH121537	MH121577	–	–	–	–	MH121419	MH121455
Diaporthe betulina	CFCC 52561	*Betula albo-sinensis*	China	MH121496	MH121538	MH121578	–	–	–	–	MH121420	MH121456
Diaporthe bicincta	CBS 121004*	*Juglans* sp.	USA	KC343134	KC343860	KC344102	–	–	–	–	KC343376	KC343618
Diaporthe biconispora	ZJUD62	*Citrus maxima*	China	KJ490597	KJ490476	KJ490418	–	–	–	–	–	KJ490539
Diaporthe biguttulata	ZJUD47	*Citrus limon*	China	KJ490582	KJ490461	KJ490403	–	–	–	–	–	KJ490524
Diaporthe biguttusis	CGMCC 3.17081	*Lithocarpus glabra*	China	KF576282	KF576257	KF576306	–	–	–	–	–	–

Diaporthe bohemiae	CBS 143347*	*Vitis vinifera*	Czech Republic	MK300012	MG281536	MG281188	–	–	–	–	MG281710	MG281361
Diaporthe brasiliensis	CBS 133183*	*Aspidosperma tomentosum*	Brazil	KC343042	KC343768	KC344010	–	–	–	–	KC343284	KC343526
Diaporthe caatingaensis	URM7485	*Tacinga inamoena*	Brazil	KY085927	KY115604	KY115601	–	–	–	–	KY115598	–
Diaporthe camelliae-oleiferae	HNZZ027*	*Camellia oleifera*	China	MZ509555	MZ504707	MZ504718	–	–	–	–	MZ504685	MZ504696
Diaporthe camelliae-sinensis	SAUCC194.92	*Camellia sinensis*	China	MT822620	MT855932	MT855817	–	–	–	–	MT855699	MT855588
Diaporthe camporesii	JZB320143	*Urtica dioidca*	Italy	MN533805	MN984254	MN561316	–	–	–	–		
Diaporthe camptothecicola	CFCC 51632	*Camptotheca acuminata*	China	KY203726	KY228887	KY228893	–	–	–	–	KY228877	KY228881
Diaporthe canthii	CPC 19740	*Canthium inerme*	South Africa	JX069864	–	–	–	–	–	–	–	–
Diaporthe caryae	CFCC 52563	*Carya illinoinensis*	China	MH121498	MH121540	MH121580	–	–	–	–	MH121422	MH121458
Diaporthe caryae	CFCC 52564	*Carya illinoinensis*	China	MH121499	MH121541	MH121581	–	–	–	–	MH121423	MH121459
Diaporthe cassines	CPC 21916	*Cassine peragua*	South Africa	KF777155	KF777244	–	–	–	–	–	–	–
Diaporthe caulivora	CBS 127268	*Glycine max*	Croatia	MH864501	KC343771	KC344013	–	–	–	–	KC343287	KC343529
Diaporthe celastrina	CBS 139.27*	*Celastrus* sp.	USA	KC343047	KC343773	KC344015	–	–	–	–	KC343289	KC343531
Diaporthe celeris	CBS 143349*	*Vitis vinifera*	United Kingdom	MG281017	MG281538	MG281190	–	–	–	–	MG281712	MG281363
Diaporthe cercidis	CFCC 52565*	*Cercis chinensis*	China	MH121500	–	MH121582	–	–	–	–	MH121424	MH121460
Diaporthe cercidis	CFCC 52566	*Cercis chinensis*	China	MH121501	–	MH121583	–	–	–	–	MH121425	MH121461
Diaporthe chamaeropis	CBS 454.81	*Chamaerops humilis*	Greece	KC343048	KC343774	KC344016	–	–	–	–	KC343290	KC343532
Diaporthe charlesworthii	BRIP 54884m*	*Rapistrum rugostrum*	Australia	KJ197288	KJ197250	KJ197268	–	–	–	–	–	–
Diaporthe chensiensis	CFCC 52567*	*Abies chensiensis*	China	MH121502	MH121544	MH121584	–	–	–	–	MH121426	MH121462
Diaporthe chensiensis	CFCC 52568	*Abies chensiensis*	China	MH121503	MH121545	MH121585	–	–	–	–	MH121427	MH121463
Diaporthe chongqingensis	PSCG 435*	*Pyrus pyrifolia*	China	MK626916	MK654866	MK691321	–	–	–	–	MK691209	MK726257
Diaporthe chromolaenae	MFLUCC 17-1422*	*Chromolaena odorata*	Thailand	MT214456			–	–	–	–		

Diaporthe cichorii	MFLUCC 17-1023*	*Cichorium intybus*	Italy	KY964220	KY964176	KY964104	–	–	–	–	KY964133	–
Diaporthe cinnamomi	CFCC 52569*	*Cinnamomum* sp.	China	MH121504	MH121546	MH121586	–	–	–	–	–	MH121464
Diaporthe cinnamomi	CFCC 52570	*Cinnamomum* sp.	China	MH121505	MH121547	MH121587	–	–	–	–	–	MH121465
Diaporthe cissampeli	CPC 27302*	*Cissampelos capensis*	South Africa	KX228273	–	KX228384	–	–	–	–	–	KX228366
Diaporthe citri	AR3405	*Citrus* sp.	USA	KC843311	KC843071	KC843187	–	–	–	–	KC843157	KJ420881
Diaporthe citri	CFCC 53079	*Citrus sinensis*	China	MK573940	MK574615	MK574635	–	–	–	–	MK574579	MK574595
Diaporthe citriasiana	CGMCC 3.15224	*Citrus unshiu*	China	JQ954645	JQ954663	KC357459	–	–	–	–	KC357491	KC490515
Diaporthe citrichinensis	CGMCC 3.15225	*Citrus* sp.	China	JQ954648	JQ954666	–	–	–	–	–	KC357494	–
Diaporthe collariana	MFLU 17-2770*	*Magnolia champaca*	Thailand	MG806115	MG783040	MG783041	–	–	–	–	MG783042	–
Diaporthe compactum	LC3083*	*Camellia sinensis*	China	KP267854	KP267928	–	–	–	–	–	–	KP293508
Diaporthe conica	CFCC 52571*	*Alangium chinense*	China	MH121506	MH121548	MH121588	–	–	–	–	MH121428	MH121466
Diaporthe conica	CFCC 52572	*Alangium chinense*	China	MH121507	MH121549	MH121589	–	–	–	–	MH121429	MH121467
Diaporthe constrictospora	GZCC 19-0065	Unknown	China	MT385947	MT424682	MT424702	–	–	–	–	MT424718	MW022487
Diaporthe constrictospora	GZCC 19-0084*	Unknown	China	MT385948	MT424683	MT424703	–	–	–	–	MT424719	MW022487
Diaporthe convolvuli	CBS 124654*	*Convolvulus arvensis*	Turkey	KC343054	KC343780	KC344022	–	–	–	–	KC343296	KC343538
Diaporthe coryli	CFCC 53083*	*Corylus mandshurica*	China	MK432661	MK578135	MK578061	–	–	–	–	MK442981	MK443006
Diaporthe coryli	CFCC 53084	*Corylus mandshurica*	China	MK432662	MK538176	MK578062	–	–	–	–	MK442982	MK443007
Diaporthe corylicola	CFCC 53986*	*Corylus heterophylla*	China	MW839880	MW815894	MW883977	–	–	–	–	MW836684	MW836717
Diaporthe corylicola	CFCC 54696	*Corylus heterophylla*	China	MW839867	MW815895	MW883978	–	–	–	–	MW836685	MW836718
Diaporthe corylicola	CFCC 54697	*Corylus heterophylla*	China	MW839882	MW815908	MW883991	–	–	–	–	MW836698	MW836731
Diaporthe crataegi	CBS 114435	*Crataegus rhipidophylla*	Sweden	KC343055	KC343781	KC344023	–	–	–	–	KC343297	KC343539
Diaporthe crotalariae	CBS 162.33*	*Crotalaria spectabilis*	USA	MH855395	GQ250307	KC344024	–	–	–	–	JX197439	KC343540
Diaporthe crousii	CAA 823	*Vaccinium corymbosum*	Portugal	MK792311	MK828081	MK837932	–	–	–	–	MK883835	MK871450
Diaporthe cucurbitae	DAOM 42078*	*Cucumis* sp.	Canada	KM453210	KM453211	KP118848	–	–	–	–	–	KM453212

Diaporthe cuppatea	CBS 117499*	*Aspalathus linearis*	South Africa	MH863021	KC343783	KC344025	–	–	–	–	KC343299	KC343541
Diaporthe cynaroidis	CBS 122676*	*Protea cynaroides*	South Africa	KC343058	KC343784	KC344026	–	–	–	–	KC343300	KC343542
Diaporthe cytosporella	FAU461	*Citrus limon*	Italy	KC843307	KC843116	KC843221	–	–	–	–	KC843141	–
Diaporthe delonicis	MFLU 16-1059	*Ipomoea batatas*	China	KP990621	KP990651	KP990631	–	–	–	–	–	KP990641
Diaporthe destruens	ZJUPD06	*Macadamia sp.*	South Africa	MN708229	MN696526	MN696537	–	–	–	–	–	–
Diaporthe diospyricola	CPC 21169*	*Diospyros whyteana*	South Africa	KF777209	–	–	–	–	–	–	–	–
Diaporthe discoidispora	ZJUD89	*Citrus unshiu*	China	KJ490624	KJ490503	KJ490445	–	–	–	–	–	KJ490566
Diaporthe donglingensis	CFCC 56581*	*Corylus heterophylla*	China	OM956090	ON157986	ON158021	–	–	–	–	–	ON157951
Diaporthe donglingensis	CFCC 57432	*Corylus heterophylla*	China	OM956091	ON157987	ON158022	–	–	–	–	–	ON157952
Diaporthe dorycnii	MFLUCC 17-1015*	*Dorycnium hirsutum*	Italy	KY964215	KY964171	KY964099	–	–	–	–	–	–
Diaporthe drenthii	BRIP 66524*	*Macadamia* sp.	Australia	MN708229	MN696526	MN696537	–	–	–	–	–	–
Diaporthe elaeagni-glabrae	LC4802	*Elaeagnus glabra*	China	KX986779	KX999171	KX999212	–	–	–	–	KX999281	KX999251
Diaporthe ellipicola	CGMCC 3.17084*	*Lithocarpus glaber*	China	KF576270	KF576245	KF576294	–	–	–	–	–	–
Diaporthe ellipsospora	GZCC 19-0231*	*decaying woody host*	Guizhou, China	MT385949	MT424684	MT424704	–	–	–	–	MT424720	MW022488
Diaporthe endophytica	CBS 133811*	*Schinus terebinthifolius*	Brazil	KC343065	KC343791	KC344033	–	–	–	–	KC343307	KC343549
Diaporthe eres	AR5193*	*Ulmus* sp.	Germany	KJ210529	KJ210550	KJ420799	–	–	–	–	KJ434999	KJ420850
Diaporthe eres	CFCC 52575	*Castanea mollissima*	China	MH121510	MH121552	MH121592	–	–	–	–	–	MH121470
Diaporthe eres	CFCC 52576	*Castanea mollissima*	China	MH121511	MH121553	MH121593	–	–	–	–	MH121432	MH121471
Diaporthe eres	CFCC 52577	*Acanthopanax senticosus*	China	MH121512	MH121554	MH121594	–	–	–	–	MH121433	MH121472
Diaporthe eres	CFCC 52578	*Sorbus* sp.	China	MH121513	MH121555	MH121595	–	–	–	–	MH121433	MH121473
Diaporthe eres	CFCC 52579	*Juglans regia*	China	MH121514	MH121556	–	–	–	–	–	–	MH121474
Diaporthe eres	CFCC 52580	*Melia azedarace*	China	MH121515	MH121557	MH121596	–	–	–	–	–	MH121475
Diaporthe eres	CFCC 52581	*Rhododendron simsii*	China	MH121516	MH121558	MH121597	–	–	–	–	–	MH121476
Diaporthe eres	**CFCC 55481**	***Euonymus japonicus***	**China**	**OQ344760**	**OQ410592**	**OQ410595**	–	–	–	–	**OQ410586**	**OQ410589**

Diaporthe eres	**CFCC 55482**	***Euonymus japonicus***	**China**	**OQ344761**	**OQ410593**	**OQ410596**	–	–	–	–	**OQ410587**	**OQ410590**
Diaporthe eres	**CFCC 55534**	***Euonymus japonicus***	**China**	**OQ344762**	**OQ410594**	**OQ410597**	–	–	–	–	**OQ410588**	**OQ410591**
Diaporthe eucalyptorum	CBS 132525*	*Eucalyptus* sp.	China	MH305525	–	–	–	–	–	–	–	–
Diaporthe foeniculacea	CBS 111553	*Foeniculum vulgare*	Spain	MH854926	KC343827	KC344069	–	–	–	–	KC343343	KC343585
Diaporthe foikelawen	CBS 145189	*Drimys winteri*	Chile	MN509713	MN509735	MN509724	–	–	–	–	MN974278	–
Diaporthe fraxini-angustifoliae	BRIP 54781*	*Fraxinus angustifolia*	Australia	JX862528	JX862534	–	–	–	–	–	KT459462	–
Diaporthe fraxinicola	CFCC 52582*	*Fraxinus chinensis*	China	MH121517	MH121560	–	–	–	–	–	MH121435	–
Diaporthe fraxinicola	CFCC 52583	*Fraxinus chinensis*	China	MH121518	MH121559	–	–	–	–	–	MH121436	–
Diaporthe fructicola	MAFF 246408*	*Passiflora edulis*	Japan	LC342734	LC342735	LC342736	–	–	–	–	LC342738	LC342737
Diaporthe fukushii	MAFF 625034	*Pyrus pyrifolia*	Japan	–	–	KJ420819	–	–	–	–	KJ435023	KJ420868
Diaporthe fulvicolor	PSCG 051*	*Pyrus pyrifolia*	China	MK626859	MK654806	MK691236	–	–	–	–	MK691132	MK726163
Diaporthe fusicola	CGMCC 3.17087	*Lithocarpus glabra*	China	KF576281	KF576256	KF576305	–	–	–	–	KF576233	–
Diaporthe ganjae	CBS 180.91*	*Cannabis sativa*	USA	KC343112	KC343838	KC344080	–	–	–	–	KC343354	KC343596
Diaporthe ganzhouensis	CFCC 53087	Unknown	China	MK432665	MK578139	MK578065	–	–	–	–	MK442985	MK443010
Diaporthe ganzhouensis	CFCC 53088	Unknown	China	MK432666	MK578140	MK578066	–	–	–	–	MK442986	MK443011
Diaporthe garethjonesii	MFLUCC 12-0542a	Unknown	Thailand	KT459423	KT459457	KT459441	–	–	–	–	KT459470	–
Diaporthe goulteri	BRIP 55657a*	*Helianthus annuus*	Australia	KJ197290	KJ197252	KJ197270	–	–	–	–	–	–
Diaporthe grandiflori	SAUCC194.84*	*Heterostemma grandiflorum*	China	MT822612	MT855809	MT855924	–	–	–	–	MT855691	MT855580
Diaporthe guangxiensis	JZB320087*	*Vitis vinifera*	China	MK335765	MK523560	MK500161	–	–	–	–	MK736720	–
Diaporthe gulyae	BRIP 54025*	*Helianthus annuus*	Australia	–	JN645803	KJ197271	–	–	–	–	–	–
Diaporthe guttulata	CGMCC 3.20100*	Unknown	China	MT385950	MT424685	MT424705	–	–	–	–	MW022470	MW022491
Diaporthe helianthi	CBS 592.81*	*Helianthus annuus*	Serbia	KC343115	KC343841	KC344083	–	–	–	–	KC343357	KC343599
Diaporthe helicis	AR5211*	*Hedera helix*	France	KJ210538	KJ210559	KJ420828	–	–	–	–	KJ435043	KJ420875

Diaporthe heliconiae	SAUCC194.77*	*Heliconia metallica*	China	MT822605	MT855802	MT855917	–	–	–	–	MT855684	MT855573
Diaporthe heterophyllae	CPC 26215	*Acacia heterophylla*	France	MG600222	MG600224	MG600226	–	–	–	–	MG600218	MG600220
Diaporthe heterostemmatis	SAUCC194.85*	*Heterostemma grandiflorum*	China	MT822613	MT855810	MT855925	–	–	–	–	MT855692	MT855581
Diaporthe hickoriae	CBS 145.26*	*Carya glabra*	USA	KC343118	KC343844	KC344086	–	–	–	–	KC343360	–
Diaporthe hispaniae	CBS 143351*	*Vitis vinifera*	Spain	MG281123	MG281644	MG281296	–	–	–	–	MG281820	MG281471
Diaporthe hongkongensis	CBS 115448*	*Dichroa febrifuga*	China	MK304388	KC343845	KC344087	–	–	–	–	KC343361	KC343603
Diaporthe huairouensis	CFCC 56808	*Corylus heterophylla*	China	ON188788	ON158016	ON158051	–	–	–	–	ON157945	ON157982
Diaporthe huairouensis	CFCC 56809	*Corylus heterophylla*	China	OM956120	ON158015	ON158050	–	–	–	–	ON157946	ON157981
Diaporthe hubeiensis	JZB320123*	*Vitis vinifera*	China	MK335809	MK523570	MK500148	–	–	–	–	MK500235	–
Diaporthe incompleta	LC6754	*Camellia sinensis*	China	KX986794	KX999186	KX999226	–	–	–	–	KX999289	KX999265
Diaporthe inconspicua	CBS 133813*	*Maytenus ilicifolia*	Brazil	–	KC343849	KC344091	–	–	–	–	KC343365	KC343607
Diaporthe infecunda	CBS 133812*	*Schinus terebinthifolius*	Brazil	KC343126	KC343852	KC344094	–	–	–	–	KC343368	KC343610
Diaporthe irregularis	CGMCC 3.20092*	Unknown	China	MT385951	MT424686	MT424706	–	–	–	–	MT424721	–
Diaporthe isoberliniae	CPC 22549	*Isoberlinia angolensis*	Zambia	KJ869190	–	KJ869245	–	–	–	–	–	–
Diaporthe juglandicola	CFCC 51134*	*Juglans mandshurica*	China	KU985101	KX024628	KX024634	–	–	–	–	KX024616	KX024622
Diaporthe juglandicola	CFCC 51135	*Juglans mandshurica*	China	KU985102	KX024629	KX024635	–	–	–	–	KX024617	KX024623
Diaporthe juglandigena	CFCC 52584	*Juglans regia*	China	MH121519	MH121561	MH121598	–	–	–	–	MH121437	MH121477
Diaporthe juglandigena	CFCC 52585	*Juglans regia*	China	MH121520	MH121562	MH121599	–	–	–	–	MH121438	MH121478
Diaporthe kadsurae	CFCC 52586*	*Kadsura longipedunculata*	China	MH121521	MH121563	MH121600	–	–	–	–	MH121439	MH121479
Diaporthe kadsurae	CFCC 52587	*Kadsura longipedunculata*	China	MH121522	MH121564	MH121601	–	–	–	–	MH121440	MH121480
Diaporthe kochmanii	BRIP 54033*	*Helianthus annuus*	Australia	–	JN645809	–	–	–	–	–	–	–
Diaporthe kongii	BRIP 54031*	*Helianthus annuus*	Australia	–	–	KJ197272	–	–	–	–	–	–

Diaporthe krabiensis	MFLUCC 17-2481*	*Bruguiera* sp.	Unknown	MN047101	MN433215	MN431495	–	–	–	–	–	–
Diaporthe lenispora	CGMCC 3.20101*	Unknown	China	MT385952	MT424687	MT424707	–	–	–	–	MW022472	MW022493
Diaporthe litchicola	BRIP 54900*	*Litchi chinensis*	Australia	LC041036	JX862539	–	–	–	–	–	–	–
Diaporthe litchii	SAUCC194.22*	*Litchi chinensis*	China	MT822550	MT855747	MT855863	–	–	–	–	MT855635	MT855519
Diaporthe lithocarpus	CGMCC 3.15175*	*Lithocarpus glabra*	China	KC135104	KC153095	KF576311	–	–	–	–	KF576235	–
Diaporthe longicicola	CGMCC 3.17089*	*Lithocarpus glabra*	China	KF576267	KF576242	KF576291	–	–	–	–	–	–
Diaporthe longicolla	FAU599	*Glycine max*	USA	KJ590728	KJ590767	KJ610883	–	–	–	–	KJ612124	KJ659188
Diaporthe longispora	CBS 194.36*	*Ribes* sp.	Canada	MH855769	KC343861	KC344103	–	–	–	–	KC343377	KC343619
Diaporthe lonicerae	MFLUCC 17-0963*	*Lonicera* sp.	Italy	KY964190	KY964146	KY964073	–	–	–	–	KY964116	–
Diaporthe lusitanicae	CBS 123212*	*Foeniculum vulgare*	Portugal	MH863279	KC343862	KC344104	–	–	–	–	KC343378	KC343620
Diaporthe lutescens	SAUCC194.36*	*Chrysalidocarpus lutescens*	China	MT822564	MT855761	MT855877	–	–	–	–	MT855647	MT855533
Diaporthe macadamiae	BRIP66526*	*Macadamia* sp.	Australia	MN708230	MN696528	MN696539	–	–	–	–	–	–
Diaporthe machili	SAUCC194.111*	*Machilus pingii*	China	MT822639	MT855951	MT855836	–	–	–	–	MT855718	MT855606
Diaporthe macintoshii	BRIP 55064a*	*Rapistrum rugosum*	Australia	KJ197289	KJ197251	KJ197269	–	–	–	–	–	–
Diaporthe mahothocarpus	CGMCC 3.15181	*Lithocarpus glabra*	China	KC153096	KC153087	KF576312	–	–	–	–	–	–
Diaporthe malorum	CAA 734	*Malus domestica*	Portugal	KY435638	KY435627	KY435668	–	–	–	–	KY435658	KY435648
Diaporthe marina	MFLU 17-2622	NA	Thailand	MN047102	–	–	–	–	–	–	–	–
Diaporthe maritima	DAOM 695742*	*Picea ruben*	Canada	KU552025	KU552023	KU574615	–	–	–	–	–	–
Diaporthe masirevicii	BRIP 54256	*Glycine max*	Australia	KJ197277	KJ197238	KJ197256	–	–	–	–	–	–
Diaporthe mayteni	CBS 133185*	*Maytenus ilicifolia*	Brazil	KC343139	KC343865	KC344107	–	–	–	–	KC343381	KC343623
Diaporthe maytenicola	CPC 21896*	*Maytenus acuminata*	South Africa	KF777157	–	KF777250	–	–	–	–	–	–
Diaporthe mediterranea	SAUCC194.111	*Machilus pingii*	China	MT822639	MT855836	MT855951	–	–	–	–	MT855718	MT855606
Diaporthe melastomatis	SAUCC194.55*	*Melastoma malabathricum*	China	MT822583	MT855780	MT855896	–	–	–	–	MT855664	MT855551
Diaporthe melonis	CBS 435.87	*Glycine soja*	Indonesia	KC343141	KC343867	KC344109	–	–	–	–	KC343383	KC343625

Diaporthe middletonii	BRIP 54884e*	*Rapistrum rugosum*	Australia	KJ197286	KJ197248	KJ197266	–	–	–	–	–	–
Diaporthe minima	GZCC 19-0066*	Unknown	China	MT385953	MT424688	MT424708	–	–	–	–	MT424722	MW022496
Diaporthe minusculata	GZCC 19-0215*	Unknown	China	MT385957	MT424692	MT424712	–	–	–	–	MW022475	MW022499
Diaporthe miriciae	BRIP 54736j*	*Helianthus annuus*	Australia	KJ197282	KJ197244	KJ197262	–	–	–	–	–	–
Diaporthe momicola	MFLUCC 16-0113	*Prunus persica*	China	KU557563	KU557631	KU55758	–	–	–	–	–	KU557611
Diaporthe multigutullata	CFCC 53095	*Citrus maxima*	China	MK432645	MK578121	MK578048	–	–	–	–	MK442967	MK442992
Diaporthe multigutullata	CFCC 53096	*Citrus maxima*	China	MK432646	MK578122	MK578049	–	–	–	–	MK442968	MK442993
Diaporthe musigena	CBS 129519*	*Musa* sp.	Australia	KC343143	KC343869	KC344111	–	–	–	–	KC343385	KC343267
Diaporthe myracrodruonis	URM7972*	*Myracrodruon urundeuva*	Unknown	MK205289	MK213408	MK205291	–	–	–	–	MK205290	–
Diaporthe neilliae	CBS 144.27*	*Spiraea* sp.	USA	KC343144	KC343870	KC344112	–	–	–	–	KC343386	KC343628
Diaporthe neoarctii	CBS 109490*	*Ambrosia trifida*	USA	KC343145	KC343871	KC344113	–	–	–	–	KC343387	KC343629
Diaporthe neoraonikayaporum	MFLUCC 14-1136	*Tectona grandis*	Thailand	KU712449	KU749369	KU743988	–	–	–	–	KU749356	–
Diaporthe nobilis	CBS 587.79	*Pinus parviflora var*	Japan	KC343153	KC343879	KC344121	–	–	–	–	KC343395	KC343637
Diaporthe nothofagi	BRIP 54801*	*Nothofagus cunninghamii*	Australia	JX862530	JX862536	KF170922	–	–	–	–	–	–
Diaporthe novem	CBS 127269*	*Glycine max*	Croatia	KC343155	KC343881	KC344123	–	–	–	–	KC343397	KC343639
Diaporthe ocoteae	CPC 26217*	*Ocotea bullata*	France	KX228293	–	KX228388	–	–	–	–	–	–
Diaporthe oraccinii	LC3166*	*Camellia sinensis*	China	KP267863	KP267937	KP293443	–	–	–	–	–	KP293517
Diaporthe ovalispora	ZJUD93	*Citrus limon*	China	KJ490628	KJ490507	KJ490449	–	–	–	–	–	KJ490570
Diaporthe ovoicicola	CGMCC 3.17093	*Lithocarpus glabra*	China	KF576265	KF576240	KF576289	–	–	–	–	KF576223	–
Diaporthe oxe	CBS 133186*	*Maytenus ilicifolia*	Brazil	KC343164	KC343890	KC344132	–	–	–	–	KC343406	KC343648
Diaporthe padina	CFCC 52590*	*Padus racemosa*	China	MH121525	MH121567	MH121604	–	–	–	–	MH121443	MH121483
Diaporthe padina	CFCC 52591	*Padus racemosa*	China	MH121526	MH121568	MH121605	–	–	–	–	MH121444	MH121484
Diaporthe pandanicola	MFLUCC 17-0607	Pandanaceae	Thailand	MG646974	–	MG646930	–	–	–	–	–	–
Diaporthe paranensis	CBS 133184*	*Maytenus ilicifolia*	Brazil	KC343171	KC343897	KC344139	–	–	–	–	KC343413	KC343655

Diaporthe parapterocarpi	CBS 137986	*Pterocarpus brenanii*	Zambia	KJ869138	–	KJ869248	–	–	–	–	–	–
Diaporthe parvae	PSCG 035	*Pyrus bretschneideri*	China	MK626920	MK654859	MK691249	–	–	–	–	MK691169	MK726211
Diaporthe pascoei	BRIP 54847*	*Persea americana*	Australia	MK111097	JX862538	KF170924	–	–	–	–	–	–
Diaporthe passiflorae	CPC 19183	*Passiflora edulis*	Netherlands	JX069860	–	–	–	–	–	–	–	–
Diaporthe passifloricola	CPC 27480*	*Passiflora foetida*	Malaysia	KX228292	–	KX228387	–	–	–	–	–	KX228367
Diaporthe penetriteum	LC3215	*Camellia sinensis*	China	KP267879	KP293532	KP267953	–	–	–	–	–	–
Diaporthe perjuncta	CBS 109745*	*Ulmus glabra*	Austria	KC343172	KC343898	KC344140	–	–	–	–	KC343414	KC343656
Diaporthe perseae	CBS 151.73	*Persea gratissima*	Netherlands	KC343173	–	–	–	–	–	–	KC343415	–
Diaporthe pescicola	MFLUCC 16-0105	*Prunus persica*	China	KU557555	KY400831	KU557579	–	–	–	–	KU557603	–
Diaporthe phaseolorum	AR4203*	*Phaseolus vulgaris*	USA	KJ590738	KJ590739	KJ610893	–	–	–	–	KJ612135	KJ659220
Diaporthe phillipsii	CAA 817	*Vaccinium corymbosum*	Portugal	MK792305	MK828076	MN000351	–	–	–	–	MK883831	MK871445
Diaporthe pimpinellae	JZB320131*	*Pimpinella peregrine*	Italy	MK874656	MT373074	MT373072	–	–	–	–	–	MT373073
Diaporthe podocarpi-macrophylli	LC6155	*Podocarpus macrophyllus*	Japan	KX986774	KX999167	KX999207	–	–	–	–	KX999278	KX999246
Diaporthe pometiae	SAUCC194.72*	*Pometia pinnata*	China	MT822600	MT855797	MT855912	–	–	–	–	MT855679	MT855568
Diaporthe pseudoalnea	CFCC 54190*	*Alnus glutinosa*	Netherlands	MZ727037	MZ816343	MZ753487	–	–	–	–	MZ753468	MZ781302
Diaporthe pseudomangiferae	CBS 101339*	*Mangifera indica*	Dominican Republic	KC343181	KC343907	KC344149	–	–	–	–	KC343423	KC343665
Diaporthe pseudophoenicicola	CBS 176.77	*Mangifera indica*	Iraq	KC343183	KC343909	KC344151	–	–	–	–	KC343425	KC343667
Diaporthe pseudotsugae	MFLU 15-3228*	*Pseudotsuga menziesii*	Italy	KY964225	KY964181	KY964108	–	–	–	–	KY964138	–
Diaporthe psoraleae	CPC 21634	*Psoralea pinnata*	South Africa	KF777158	KF777245	KF777251	–	–	–	–	–	–
Diaporthe psoraleae-pinnatae	CPC 21638*	*Psoralea pinnata*	South Africa	KF777159	–	KF777252	–	–	–	–	–	–
Diaporthe pterocarpi	MFLUCC 10-0571*	*Pterocarpus indicus*	Thailand	JQ619899	JX275416	JX275460	–	–	–	–	JX197451	–
Diaporthe pterocarpicola	MFLUCC 10-0580a*	*Pterocarpus indicus*	Thailand	JQ619887	JX275403	JX275441	–	–	–	–	JX197433	–

Diaporthe pulla	CBS 338.89*	*Hedera helix*	Yugoslavia	KC343152	KC343878	KC344120	–	–	–	–	KC343394	KC343636
Diaporthe pungensis	SAUCC194.112*	*Elaeagnus pungens*	China	MT822640	MT855837	MT855952	–	–	–	–	MT855719	MT855607
Diaporthe pyracanthae	CAA483	*Pyracantha coccinea*	Portugal	KY435635	KY435625	KY435666	–	–	–	–	KY435645	KY435656
Diaporthe racemosae	CPC 26646	*Euclea racemosa*	South Africa	MG600223	MG600225	MG600227	–	–	–	–	MG600219	MG600221
Diaporthe raonikayaporum	CBS 133182	*Spondias mombin*	Brazil	KC343188	KC343914	KC344156	–	–	–	–	KC343430	KC343672
Diaporthe ravennica	MFLUCC 16-0997	*Clematis vitalba*	Italy	–	MT394670	–	–	–	–	–	–	–
Diaporthe rhusicola	CPC 18191	*Rhus pendulina*	South Africa	JF951146	–	–	–	–	–	–	–	–
Diaporthe rosae	MFLUCC 17-2658	*Rosa* sp.	United Kingdom	MG828894	–	MG843878	–	–	–	–	MG829273	–
Diaporthe rosicola	MFLU 17-0646*	*Rosa* sp.	United Kingdom	MG828895	MG829270	MG843877	–	–	–	–	MG829274	–
Diaporthe rosiphthora	COAD 2914*	*Rosa* sp.	Brazil	MT311197	MT313693	–	–	–	–	–	MT313691	–
Diaporthe rossmaniae	CAA 762*	*Vaccinium corymbosum*	Portugal	MK792290	MK828063	MK837914	–	–	–	–	MK883822	MK871432
Diaporthe rostrata	CFCC 50062*	*Juglans mandshurica*	China	KP208847	KP208853	KP208855	–	–	–	–	KP208849	KP208851
Diaporthe rostrata	CFCC 50063	*Juglans mandshurica*	China	KP208848	KP208854	KP208856	–	–	–	–	KP208850	KP208852
Diaporthe rudis	AR3422*	*Laburnum anagyroides*	Austria	KC843331	KC843090	KC843177	–	–	–	–	KC843146	–
Diaporthe saccarata	CBS 116311*	*Protea repens*	South Africa	KC343190	KC343916	KC344158	–	–	–	–	KC343432	KC343674
Diaporthe sackstonii	BRIP 54669b*	*Helianthus annuus*	Australia	KJ197287	KJ197249	KJ197267	–	–	–	–	–	–
Diaporthe salicicola	BRIP 54825*	*Salix purpurea*	Australia	JX862531	JX862537	KF170923	–	–	–	–	–	–
Diaporthe sambucusii	CFCC 51986*	*Sambucus williamsii*	China	KY852495	KY852507	KY852511	–	–	–	–	KY852499	KY852503
Diaporthe sambucusii	CFCC 51987	*Sambucus williamsii*	China	KY852496	KY852508	KY852512	–	–	–	–	KY852500	KY852504
Diaporthe schimae	CFCC 53103	*Schima superba*	China	MK442640	MK578116	MK578043	–	–	–	–	MK442962	MK442987
Diaporthe schimae	CFCC 53104	*Schima superba*	China	MK442641	MK578117	MK578044	–	–	–	–	MK442963	MK442988
Diaporthe schimae	CFCC 53105	*Schima superba*	China	MK442642	MK578118	MK578045	–	–	–	–	MK442964	MK442989
Diaporthe schini	CBS 133181*	*Schinus terebinthifolius*	Brazil	KC343191	KC343917	KC344159	–	–	–	–	KC343433	KC343675
Diaporthe schisandrae	CFCC 51988*	*Schisandra chinensis*	China	KY852497	KY852509	KY852513	–	–	–	–	KY852501	KY852505
Diaporthe schisandrae	CFCC 51989	*Schisandra chinensis*	China	KY852498	KY852510	KY852514	–	–	–	–	KY852502	KY852506

Diaporthe schoeni	MFLU 15-1279*	*Schoenus nigricans*	Italy	KY964226	KY964182	KY964109	–	–	–	–	KY964139	–
Diaporthe sclerotioides	CBS 296.67	*Cucumis sativus*	Netherlands	MH858974	KC343919	KC344161	–	–	–	–	KC343435	KC343677
Diaporthe searlei	BRIP 66528*	*Macadamia* sp.	Australia	MN708231	–	MN696540	–	–	–	–	–	–
Diaporthe sennae	CFCC 51636*	*Senna bicapsularis*	China	KY203724	KY228885	KY228891	–	–	–	–	KY228875	–
Diaporthe sennae	CFCC 51637	*Senna bicapsularis*	China	KY203725	KY228886	KY228892	–	–	–	–	KY228876	–
Diaporthe sennicola	CFCC 51634*	*Senna bicapsularis*	China	KY203722	KY228883	KY228889	–	–	–	–	KY228873	KY228879
Diaporthe sennicola	CFCC 51635	*Senna bicapsularis*	China	KY203723	KY228884	KY228890	–	–	–	–	KY228874	KY228880
Diaporthe serafiniae	BRIP 55665a*	*Helianthus annuus*	Australia	KJ197274	KJ197236	KJ197254	–	–	–	–	–	–
Diaporthe shaanxiensis	CFCC 53106	*Liana* sp.	China	MK432654	MK578130	–	–	–	–	–	MK442976	MK443001
Diaporthe shaanxiensis	CFCC 53107	*Liana* sp.	China	MK432655	MK578131	–	–	–	–	–	MK432977	MK432002
Diaporthe siamensis	MFLUCC 10-0573a	*Dasymaschalon* sp.	Thailand	–	JX275393	JX275429	–	–	–	–	JQ619897	–
Diaporthe silvicola	CFCC 54191*	*Fraxinus excelsior*	Netherlands	MZ727041	MZ816347	MZ753491	–	–	–	–	MZ753472	MZ753481
Diaporthe sojae	FAU635*	*Glycine max*	USA	KJ590719	KJ590762	KJ610875	–	–	–	–	KJ612116	KJ659208
Diaporthe spartinicola	CPC 24951	*Spartium junceμm*	Spain	KR611879	–	KR857695	–	–	–	–	–	KR857696
Diaporthe spinosa	PSCG 383*	*Pyrus pyrifolia*	China	MK626849	MK654811	MK691234	–	–	–	–	MK691129	MK726156
Diaporthe sterilis	CBS 136969*	*Vaccinium corymbosum*	Italy	KJ160579	KJ160611	KJ160528	–	–	–	–	KJ160548	MF418350
Diaporthe stictica	CBS 370.54	*Buxus sampervirens*	Italy	KC343212	KC343938	KC344180	–	–	–	–	KC343454	KC343696
Diaporthe subclavata	ZJUD95	*Citrus unshiu*	China	KJ490630	KJ490509	KJ490451	–	–	–	–	–	KJ490572
Diaporthe subcylindrospora	KUMCC 17-0151	Unknown	China	MG746629	MG746630	MG746631	–	–	–	–	–	–
Diaporthe subellipicola	KUMCC 17-0153	Unknown	China	MG746632	MG746633	MG746634	–	–	–	–	–	–
Diaporthe subordinaria	CBS 464.90	*Plantago lanceolata*	South Africa	KC343214	KC343940	KC344182	–	–	–	–	KC343456	KC343698
Diaporthe taoicola	MFLUCC 16-0117	*Prunus persica*	China	KU557567	KU557636	KU557591	–	–	–	–	–	–
Diaporthe tarchonanthi	CBS 146073*	*Tarchonanthus littoralis*	South Africa	MT223794	MT223759	MT223733	–	–	–	–	–	–
Diaporthe tectonae	MFLUCC 12-0777	*Tectona grandis*	Thailand	KU712430	KU749359	KU743977	–	–	–	–	KU749345	–

Diaporthe tectonendophytica	MFLUCC 13-0471	*Tectona grandis*	Thailand	KU712439	KU749367	KU743986	–	–	–	–	KU749354	–
Diaporthe tectonigena	MFLUCC 12-0767	*Camellia sinensis*	China	KX986782	KX999174	KX999214	–	–	–	–	KX999284	KX999254
Diaporthe terebinthifolii	CBS 133180*	*Schinus terebinthifolius*	Brazil	KC343216	KC343942	KC344184	–	–	–	–	KC343458	KC343700
Diaporthe ternstroemia	CGMCC 3.15183	*Ternstroemia gymnanthera*	China	KC153098	KC153089	–	–	–	–	–	–	–
Diaporthe thunbergii	MFLUCC 10-0576a*	*Thunbergia laurifolia*	Thailand	JQ619893	JX275409	–	–	–	–	–	JX197440	–
Diaporthe thunbergiicola	MFLUCC 12-0033*	*Thunbergia laurifolia*	Thailand	KP715097	KP715098	–	–	–	–	–	–	–
Diaporthe tibetensis	CFCC 51999*	*Juglandis regia*	China	MF279843	MF279858	MF279873	–	–	–	–	MF279888	MF279828
Diaporthe tibetensis	CFCC 52000	*Juglandis regia*	China	MF279844	MF279859	MF279874	–	–	–	–	MF279889	MF279829
Diaporthe torilicola	MFLUCC 17-1051*	*Torilis arvensis*	Italy	KY964212	KY964168	KY964096	–	–	–	–	KY964127	–
Diaporthe toxica	CBS 534.93*	*Lupinus angustifolius*	Australia	KC343220	KC343946	KC344188	–	–	–	–	KC343462	KC343704
Diaporthe tulliensis	BRIP 62248a	*Theobroma cacao*	Australia	KR936130	KR936133	KR936132	–	–	–	–	–	–
Diaporthe ueckerae	FAU656*	*Cucumis melo*	USA	KJ590726	KJ590747	KJ610881	–	–	–	–	KJ612122	KJ659215
Diaporthe ukurunduensis	CFCC 52592*	*Acer ukurunduense*	China	MH121527	MH121569	–	–	–	–	–	MH121445	MH121485
Diaporthe ukurunduensis	CFCC 52593	*Acer ukurunduense*	China	MH121528	MH121570	–	–	–	–	–	MH121446	MH121486
Diaporthe undulata	LC6624	Unknown	China	KX986798	KX999190	KX999230	–	–	–	–	–	KX999269
Diaporthe unshiuensis	CFCC 52594	*Carya illinoensis*	China	MH121529	MH121571	MH121606	–	–	–	–	MH121447	MH121487
Diaporthe unshiuensis	CFCC 52595	*Carya illinoensis*	China	MH121530	MH121572	MH121607	–	–	–	–	MH121448	MH121488
Diaporthe vaccinii	CBS 160.32*	*Oxycoccus macrocarpos*	USA	MH121502	MH121544	MH121584	–	–	–	–	MH121426	MH121462
Diaporthe vacuae	CAA830	*Vaccinium corymbosum*	Portugal	MK792306	MK828077	MK837928	–	–	–	–	MK883832	MK871446
Diaporthe vangueriae	CBS 137985*	*Vangueria infausta*	Zambia	KJ869137	–	KJ869247	–	–	–	–	–	–
Diaporthe vawdreyi	BRIP 57887a	*Psidium guajava*	Australia	KR936126	KR936129	KR936128	–	–	–	–	–	–
Diaporthe velutina	LC4421	*Neolitsea* sp.	China	KX986790	KX999182	KX999223	–	–	–	–	–	KX999261
Diaporthe verniciicola	CFCC 53109	*Vernicia montana*	China	MK573944	MK574619	MK574639	–	–	–	–	MK574583	MK574599
Diaporthe verniciicola	CFCC 53110	*Vernicia montana*	China	MK573945	MK574620	MK574640	–	–	–	–	MK574584	MK574600

Diaporthe viniferae	JZB320071*	*Vitis vinifera*	China	MK341551	MK500107	MK500112	–	–	–	–	MK500119	–
Diaporthe virgiliae	CMW 40748	*Virgilia oroboides*	South Africa	KP247556	–	KP247575	–	–	–	–	–	–
Diaporthe xishuangbanica	LC6707	*Camellia sinensis*	China	KX986783	KX999175	KX999216	–	–	–	–	–	KX999255
Diaporthe xunwuensis	CFCC 53085	Unknown	China	MK432663	MK578137	MK578063	–	–	–	–	MK442983	MK443008
Diaporthe xunwuensis	CFCC 53086	Unknown	China	MK432664	MK578138	MK578064	–	–	–	–	MK442984	MK443009
Diaporthe yunnanensis	LC6168	Unknown	China	KX986796	KX999188	KX999228	–	–	–	–	KX999290	KX999267
Diaporthe zaobaisu	PSCG 031*	*Pyrus bretschneideri*	China	MK626922	MK654855	MK691245	–	–	–	–	–	MK726207
Diaporthella corylina	CBS 121124	*Corylus* sp.	NA	KC343004	KC343730	KC343972	–	–	–	–	KC343246	KC343488
Neopestalotiopsis magna	MFLUCC 12-0652*	*Pteridium* sp.	France	KF582795	KF582791	KF582793	–	–	–	–	–	–
Pestalotiopsis abietis	CFCC 53011*	*Abies fargesii*	China	MK397013	MK622277	MK622280	–	–	–	–	–	–
Pestalotiopsis abietis	CFCC 53012	*Abies fargesii*	China	MK397014	MK622278	MK622281	–	–	–	–	–	–
Pestalotiopsis adusta	ICMP 6088*	refrigerator door	Fiji	JX399006	JX399070	JX399037	–	–	–	–	–	–
Pestalotiopsis adusta	MFLUCC 10-146	*Syzygium* sp.	Thailand	JX399007	JX399071	JX399038	–	–	–	–	–	–
Pestalotiopsis aggestorum	LC6301*	*Camellia sinensis*	China	KX895015	KX895234	KX895348	–	–	–	–	–	–
Pestalotiopsis aggestorum	LC8186	*Camellia sinensis*	China	KY464140	KY464150	KY464160	–	–	–	–	–	–
Pestalotiopsis anacardiacearum	IFRDCC 2397*	*Mangifera indica*	China	KC247154	KC247156	KC247155	–	–	–	–	–	–
Pestalotiopsis anhuiensis	CFCC 54791*	*Cyclobalanopsis glauca*	China	ON007028	ON005045	ON005056	–	–	–	–	–	–
Pestalotiopsis arceuthobii	CBS 434.65*	*Arceuthobium campylopodum*	USA	KM199341	KM199516	KM199427	–	–	–	–	–	–
Pestalotiopsis arenga	CBS 331.92*	*Arenga undulatifolia*	Singapore	KM199340	KM199515	KM199426	–	–	–	–	–	–
Pestalotiopsis australasiae	CBS 114126*	*Knightia* sp.	New Zealand	KM199297	KM199499	KM199409	–	–	–	–	–	–
Pestalotiopsis australasiae	CBS 114141	*Protea* sp.	New South Wales	KM199298	KM199501	KM199410	–	–	–	–	–	–
Pestalotiopsis australis	CBS 111503	*Protea neriifolia* × *susannae*	South Africa	KM199331	KM199557	KM199382	–	–	–	–	–	–
Pestalotiopsis australis	CBS 114193*	*Grevillea* sp.	New South Wales	KM199332	KM199475	KM199383	–	–	–	–	–	–

Pestalotiopsis biciliata	CBS 124463*	*Platanus* × *hispanica*	Slovakia	KM199308	KM199505	KM199399	–	–	–	–	–	–
Pestalotiopsis biciliata	CBS 236.38	*Paeonia* sp.	Italy	KM199309	KM199506	KM199401	–	–	–	–	–	–
Pestalotiopsis brachiata	LC2988*	*Camellia* sp.	China	KX894933	KX895150	KX895265	–	–	–	–	–	–
Pestalotiopsis brachiata	LC8188	*Camellia* sp.	China	KY464142	KY464152	KY464162	–	–	–	–	–	–
Pestalotiopsis brachiata	LC8189	*Camellia* sp.	China	KY464143	KY464153	KY464163	–	–	–	–	–	–
Pestalotiopsis brassicae	CBS 170.26*	*Brassica napus*	New Zealand	KM199379	KM199558	–	–	–	–	–	–	–
Pestalotiopsis camelliae	MFLUCC 12-0277*	*Camellia japonica*	China	JX399010	JX399074	JX399041	–	–	–	–	–	–
Pestalotiopsis camelliae-oleiferae	CSUFTCC08*	*Camelliae oleiferae*	China	OK493593	OK507963	OK562368	–	–	–	–	–	–
Pestalotiopsis camelliae-oleiferae	CSUFTCC09	*Camelliae oleiferae*	China	OK493594	OK507964	OK562369	–	–	–	–	–	–
Pestalotiopsis castanopsidis	CFCC 54430*	*Castanopsis lamontii*	China	OK339732	OK358493	OK358508	–	–	–	–	–	–
Pestalotiopsis castanopsidis	CFCC 54305	*Castanopsis hystrix*	China	OK339733	OK358494	OK358509	–	–	–	–	–	–
Pestalotiopsis castanopsidis	CFCC 54384	*Castanopsis hystrix*	China	OK339734	OK358495	OK358510	–	–	–	–	–	–
Pestalotiopsis chamaeropis	CBS 186.71*	*Chamaerops humilis*	Italy	KM199326	KM199473	KM199391	–	–	–	–	–	–
Pestalotiopsis chamaeropis	LC3619	*Camellia* sp.	China	KX894991	KX895208	KX895322	–	–	–	–	–	–
Pestalotiopsis chamaeropis	CFCC 55124	*Quercus acutissima*	China	OM746221	OM839993	OM839894	–	–	–	–	–	–
Pestalotiopsis chamaeropis	CFCC 54977	*Quercus acutissima*	China	OM746223	OM839995	OM839896	–	–	–	–	–	–
Pestalotiopsis chamaeropis	CFCC 55019	*Quercus aliena*	China	OM746224	OM839996	OM839897	–	–	–	–	–	–
Pestalotiopsis chamaeropis	CFCC 55122	*Quercus aliena*	China	OM746229	OM840001	OM839902	–	–	–	–	–	–
Pestalotiopsis chamaeropis	CFCC 55023	*Castanopsis fissa*	China	OM746233	OM840005	OM839906	–	–	–	–	–	–

Pestalotiopsis chamaeropis	CFCC 54776	*Quercus variabilis*	China	OM746234	OM840006	OM839907	–	–	–	–	–	–
Pestalotiopsis chamaeropis	CFCC 55338	*Quercus variabilis*	China	OM746235	OM840007	OM839908	–	–	–	–	–	–
Pestalotiopsis changjiangensis	CFCC 54314*	*Castanopsis tonkinensis*	China	OK339739	OK358500	OK358515	–	–	–	–	–	–
Pestalotiopsis changjiangensis	CFCC 54433	*Castanopsis hainanensis*	China	OK339740	OK358501	OK358516	–	–	–	–	–	–
Pestalotiopsis changjiangensis	CFCC 52803	*Cyclobalanopsis austrocochinchinensis*	China	OK339741	OK358502	OK358517	–	–	–	–	–	–
Pestalotiopsis chaoyangensis	**CFCC 55549***	***Euonymus japonicus***	**China**	**OQ344763**	**OQ410582**	**OQ410584**	–	–	–	–	–	–
Pestalotiopsis chaoyangensis	**CFCC 58805**	***Euonymus japonicus***	**China**	**OQ344764**	**OQ410583**	**OQ410585**	–	–	–	–	–	–
Pestalotiopsis clavata	MFLUCC 12-0268*	*Buxus* sp.	China	JX398990	JX399056	JX399025	–	–	–	–	–	–
Pestalotiopsis colombiensis	CBS 118553*	*Eucalyptus eurograndis*	Colombia	KM199307	KM199488	KM199421	–	–	–	–	–	–
Pestalotiopsis cyclobalanopsidis	CFCC 54328*	*Cyclobalanopsis glauca*	China	OK339735	OK358496	OK358511	–	–	–	–	–	–
Pestalotiopsis cyclobalanopsidis	CFCC 55891	*Cyclobalanopsis glauca*	China	OK339736	OK358497	OK358512	–	–	–	–	–	–
Pestalotiopsis digitalis	MFLU 14-0208*	*Digitalis purpurea*	New Zealand	KP781879	–	KP781883	–	–	–	–	–	–
Pestalotiopsis dilucida	LC3232	*Camellia sinensis*	China	KX894961	KX895178	KX895293	–	–	–	–	–	–
Pestalotiopsis dilucida	LC8184	*Camellia sinensis*	China	KY464138	KY464148	KY464158	–	–	–	–	–	–
Pestalotiopsis diploclisiae	CBS 115449	*Psychotria tutcheri*	China	KM199314	KM199485	KM199416	–	–	–	–	–	–
Pestalotiopsis diploclisiae	CBS 115587*	*Diploclisia glaucescens*	China	KM199320	KM199486	KM199419	–	–	–	–	–	–
Pestalotiopsis disseminata	CBS 143904	*Persea americana*	New Zealand	MH554152	MH554587	MH554825	–	–	–	–	–	–
Pestalotiopsis disseminata	MEAN 1165	*Pinus pinea*	Portugal	MT374687	MT374699	MT374712	–	–	–	–	–	–
Pestalotiopsis diversiseta	MFLUCC 12-0287*	*Rhododendron* sp.	China	JX399009	JX399073	JX399040	–	–	–	–	–	–

Pestalotiopsis doitungensis	MFLUCC 14-0115*	*Dendrobium* sp.	Thailand	MK993574	MK975832	MK975837	–	–	–	–	–	–
Pestalotiopsis dracaenicola	MFLUCC 18-0913*	*Dracaena* sp.	Thailand	MN962731	MN962732	MN962733	–	–	–	–	–	–
Pestalotiopsis dracontomelonis	MFLU 14-0207*	*Dracontomelon dao*	Thailand	KP781877	KP781880	–	–	–	–	–	–	–
Pestalotiopsis ericacearum	IFRDCC 2439*	*Rhododendron delavayi*	China	KC537807	KC537814	KC537821	–	–	–	–	–	–
Pestalotiopsis etonensis	BRIP 66615*	*Sporobolus jacquemontii*	Australia	MK966339	MK977635	MK977634	–	–	–	–	–	–
Pestalotiopsis formosana	NTUCC 17-009*	*Poaceae* sp.	China	MH809381	MH809389	MH809385	–	–	–	–	–	–
Pestalotiopsis furcata	MFLUCC 12-0054*	*Camellia sinensis*	Thailand	JQ683724	JQ683740	JQ683708	–	–	–	–	–	–
Pestalotiopsis furcata	LC6691	*Camellia sinensis*	China	KX895030	KX895248	KX895363	–	–	–	–	–	–
Pestalotiopsis foliicola	CFCC 54440*	*Castanopsis faberi*	China	ON007029	ON005046	ON005057	–	–	–	–	–	–
Pestalotiopsis foliicola	CFCC 57359	*Castanopsis faberi*	China	ON007030	ON005047	ON005058	–	–	–	–	–	–
Pestalotiopsis foliicola	CFCC 57360	*Castanopsis faberi*	China	ON007031	ON005048	ON005059	–	–	–	–	–	–
Pestalotiopsis gaultheriae	IFRD 411-014*	*Gaultheria forrestii*	China	KC537805	KC537812	KC537819	–	–	–	–	–	–
Pestalotiopsis gibbosa	NOF 3175*	*Gaultheria shallon*	Canada	LC311589	LC311591	LC311590	–	–	–	–	–	–
Pestalotiopsis grevilleae	CBS 114127*	*Grevillea* sp.	Australia	KM199300	KM199504	KM199407	–	–	–	–	–	–
Pestalotiopsis guangxiensis	CFCC 54308*	*Quercus griffithii*	China	OK339737	OK358498	OK358513	–	–	–	–	–	–
Pestalotiopsis guangxiensis	CFCC 54300	*Quercus griffithii*	China	OK339738	OK358499	OK358514	–	–	–	–	–	–
Pestalotiopsis guizhouensis	CFCC 54803*	*Cyclobalanopsis glauca*	China	ON007035	ON005052	ON005063	–	–	–	–	–	–
Pestalotiopsis guizhouensis	CFCC 57364	*Cyclobalanopsis glauca*	China	ON007036	ON005053	ON005064	–	–	–	–	–	–
Pestalotiopsis hawaiiensis	CBS 114491*	*Leucospermum* sp.	USA	KM199339	KM199514	KM199428	–	–	–	–	–	–
Pestalotiopsis hispanica	CBS 115391*	*Protea* sp.	Spain	MH553981	MH554399	MH554640	–	–	–	–	–	–
Pestalotiopsis hollandica	CBS 265.33*	*Sciadopitys verticillata*	Netherlands	KM199328	KM199481	KM199388	–	–	–	–	–	–

Pestalotiopsis humicola	CBS 336.97*	soil	Papua New Guinea	KM199317	KM199484	KM199420	–	–	–	–	–	–
Pestalotiopsis hunanensis	CSUFTCC15*	*Camellia oleifera*	China	OK493599	OK507969	OK562374	–	–	–	–	–	–
Pestalotiopsis hunanensis	CSUFTCC18	*Camellia oleifera*	China	OK493600	OK507970	OK562375	–	–	–	–	–	–
Pestalotiopsis inflexa	MFLUCC 12-0270*	unidentified tree	China	JX399008	JX399072	JX399039	–	–	–	–	–	–
Pestalotiopsis intermedia	MFLUCC 12-0259*	unidentified tree	China	JX398993	JX399059	JX399028	–	–	–	–	–	–
Pestalotiopsis italiana	MFLU 14-0214*	*Cupressus glabra*	Italy	KP781878	KP781881	KP781882	–	–	–	–	–	–
Pestalotiopsis jesteri	CBS 109350*	*Fragraea bodenii*	Papua New Guinea	KM199380	KM199554	KM199468	–	–	–	–	–	–
Pestalotiopsis jiangxiensis	LC4399*	*Camellia* sp.	China	KX895009	KX895227	KX895341	–	–	–	–	–	–
Pestalotiopsis jinchanghensis	LC6636*	*Camellia sinensis*	China	KX895028	KX895247	KX895361	–	–	–	–	–	–
Pestalotiopsis jinchanghensis	LC8190	*Camellia sinensis*	China	KY464144	KY464154	KY464164	–	–	–	–	–	–
Pestalotiopsis kaki	KNU-PT-1804*	*Diospyros kaki*	Korea	LC552953	LC553555	LC552954	–	–	–	–	–	–
Pestalotiopsis kandelicola	NCYU 19-0355*	*Kandelia candel*	China	MT560723	MT563102	MT563100	–	–	–	–	–	–
Pestalotiopsis kenyana	CBS 442.67*	*Coffea* sp.	Kenya	KM199302	KM199502	KM199395	–	–	–	–	–	–
Pestalotiopsis kenyana	LC6633	*Camellia sinensis*	China	KX895027	KX895246	KX895360	–	–	–	–	–	–
Pestalotiopsis kenyana	CFCC 54962	*Quercus aliena*	China	OM746237	OM840009	OM839910	–	–	–	–	–	–
Pestalotiopsis kenyana	CFCC 55330	*Cyclobalanopsis fleuryi*	China	OM746238	OM840010	OM839911	–	–	–	–	–	–
Pestalotiopsis kenyana	CFCC 54621	*Quercus aliena* var. *acutiserrata*	China	OM746239	OM840011	OM839912	–	–	–	–	–	–
Pestalotiopsis kenyana	CFCC 54732	*Cyclobalanopsis neglecta*	China	OM746243	OM840015	OM839916	–	–	–	–	–	–
Pestalotiopsis kenyana	CFCC 54742	*Castanopsis hystrix*	China	OM746245	OM840017	OM839918	–	–	–	–	–	–
Pestalotiopsis kenyana	CFCC 54618	*Quercus aliena* var. *acutiserrata*	China	OM746248	OM840020	OM839921	–	–	–	–	–	–
Pestalotiopsis kenyana	CFCC 54805	*Cyclobalanopsis glauca*	China	OM746253	OM840025	OM839926	–	–	–	–	–	–
Pestalotiopsis kenyana	CFCC 55088	*Castanopsis fissa*	China	OM746254	OM840026	OM839927	–	–	–	–	–	–
Pestalotiopsis knightiae	CBS 111963	*Knightia* sp.	New Zealand	KM199311	KM199495	KM199406	–	–	–	–	–	–

Pestalotiopsis knightiae	CBS 114138*	*Knightia* sp.	New Zealand	KM199310	KM199497	KM199408	–	–	–	–	–	–
Pestalotiopsis krabiensis	MFLUCC 16-0260*	*Pandanus* sp.	Thailand	MH388360	MH388395	MH412722	–	–	–	–	–	–
Pestalotiopsis leucadendri	CBS 121417*	*Leucadendron* sp.	South Africa	MH553987	MH554412	MH554654	–	–	–	–	–	–
Pestalotiopsis licualicola	HGUP 4057*	*Licuala grandis*	China	KC492509	KC481684	KC481683	–	–	–	–	–	–
Pestalotiopsis lijiangensis	CFCC 50738*	*Castanopsis carlesii* var. *spinulosa*	China	KU860520	KU844185	KU844184	–	–	–	–	–	–
Pestalotiopsis linearis	MFLUCC 12-0271*	*Trachelospermum* sp.	China	JX398992	JX399058	JX399027	–	–	–	–	–	–
Pestalotiopsis lithocarpi	CFCC 55100*	*Lithocarpus chiungchungensis*	China	OK339742	OK358503	OK358518	–	–	–	–	–	–
Pestalotiopsis lithocarpi	CFCC 55893	*Lithocarpus chiungchungensis*	China	OK339743	OK358504	OK358519	–	–	–	–	–	–
Pestalotiopsis lushanensis	LC4344*	*Camellia* sp.	China	KX895005	KX895223	KX895337	–	–	–	–	–	–
Pestalotiopsis lushanensis	LC8182	*Camellia* sp.	China	KY464136	KY464146	KY464156	–	–	–	–	–	–
Pestalotiopsis lushanensis	LC8183	*Camellia* sp.	China	KY464137	KY464147	KY464157	–	–	–	–	–	–
Pestalotiopsis lushanensis	CFCC 54894	*Quercus serrata*	China	OM746282	OM840054	OM839955	–	–	–	–	–	–
Pestalotiopsis macadamiae	BRIP 63738b	*Macadamia integrifolia*	Australia	KX186588	KX186621	KX186680	–	–	–	–	–	–
Pestalotiopsis macadamiae	BRIP 63739b	*Macadamia integrifolia*	Australia	KX186587	KX186620	KX186679	–	–	–	–	–	–
Pestalotiopsis macadamiae	BRIP 637441a	*Macadamia integrifolia*	Australia	KX186586	KX186619	KX186678	–	–	–	–	–	–
Pestalotiopsis malayana	CBS 102220*	*Macaranga triloba*	Malaysia	KM199306	KM199482	KM199411	–	–	–	–	–	–
Pestalotiopsis monochaeta	CBS 144.97*	*Quercus robur*	Netherlands	KM199327	KM199479	KM199386	–	–	–	–	–	–
Pestalotiopsis monochaeta	CBS 440.83	*Taxus baccata*	Netherlands	KM199329	KM199480	KM199387	–	–	–	–	–	–

Pestalotiopsis nanjingensis	CFCC 53882	*Quercus aliena*	China	OM746295	OM840067	OM839968	–	–	–	–	–	–
Pestalotiopsis nanjingensis	CSUFTCC16*	*Camellia oleifera*	China	OK493602	OK507972	OK562377	–	–	–	–	–	–
Pestalotiopsis nanningensis	CSUFTCC10*	*Camellia oleifera*	China	OK493596	OK507966	OK562371	–	–	–	–	–	–
Pestalotiopsis neolitseae	NTUCC 17-011*	*Neolitsea villosa*	China	MH809383	MH809391	MH809387	–	–	–	–	–	–
Pestalotiopsis neolitseae	CFCC 54590	*Lithocarpus amygdalifolius*	China	OK339744	OK358505	OK358520	–	–	–	–	–	–
Pestalotiopsis novae-hollandiae	CBS 130973*	*Banksia grandis*	Australia	KM199337	KM199511	KM199425	–	–	–	–	–	–
Pestalotiopsis oryzae	CBS 111522	*Telopea* sp.	USA	KM199294	KM199493	KM199394	–	–	–	–	–	–
Pestalotiopsis oryzae	CBS 171.26	NA	Italy	KM199304	KM199494	KM199397	–	–	–	–	–	–
Pestalotiopsis oryzae	CBS 353.69*	*Oryza sativa*	Denmark	KM199299	KM199496	KM199398	–	–	–	–	–	–
Pestalotiopsis pallidotheae	MAFF 240993*	*Pieris japonica*	Japan	AB482220	–	–	–	–	–	–	–	–
Pestalotiopsis pandanicola	MFLUCC 16-0255*	*Pandanus* sp.	Thailand	MH388361	MH388396	MH412723	–	–	–	–	–	–
Pestalotiopsis papuana	CBS 331.96*	coastal soil	Papua New Guinea	KM199321	KM199491	KM199413	–	–	–	–	–	–
Pestalotiopsis papuana	CBS 887.96	*Cocos nucifera*	Papua New Guinea	KM199318	KM199492	KM199415	–	–	–	–	–	–
Pestalotiopsis parva	CBS 265.37	*Delonix regia*	NA	KM199312	KM199508	KM199404	–	–	–	–	–	–
Pestalotiopsis parva	CBS 278.35*	*Delonix regia*	NA	KM199313	KM199509	KM199405	–	–	–	–	–	–
Pestalotiopsis photiniicola	GZCC 16-0028*	*Photinia serrulata*	China	KY092404	KY047662	KY047663	–	–	–	–	–	–
Pestalotiopsis pini	MEAN 1092	*Pinus pinea*	Portugal	MT374680	MT374693	MT374705	–	–	–	–	–	–
Pestalotiopsis pinicola	KUMCC 19-0183*	*Pinus armandii*	China	MN412636	MN417509	MN417507	–	–	–	–	–	–
Pestalotiopsis portugalica	CBS 393.48*	NA	Portugal	KM199335	KM199510	KM199422	–	–	–	–	–	–
Pestalotiopsis rhizophorae	MFLUCC 17-0416*	*Rhizophora mucronata*	Thailand	MK764283	MK764327	MK764349	–	–	–	–	–	–
Pestalotiopsis rhododendri	IFRDCC 2399*	*Rhododendron sinogrande*	China	KC537804	KC537811	KC537818	–	–	–	–	–	–

Pestalotiopsis rhodomyrtus	LC4458	*Camellia sinensis*	China	KX895010	KX895228	KX895342	–	–	–	–	–	–
Pestalotiopsis rhodomyrtus	HGUP4230*	*Rhodomyrtus tomentosa*	China	KF412648	KF412645	KF412642	–	–	–	–	–	–
Pestalotiopsis rhodomyrtus	CFCC 54733	*Quercus aliena*	China	OM746310	OM840082	OM839983	–	–	–	–	–	–
Pestalotiopsis rhodomyrtus	CFCC 55052	*Cyclobalanopsis augustinii*	China	OM746311	OM840083	OM839984	–	–	–	–	–	–
Pestalotiopsis rosea	MFLUCC 12-0258*	*Pinus* sp.	China	JX399005	JX399069	JX399036	–	–	–	–	–	–
Pestalotiopsis scoparia	CBS 176.25*	*Chamaecyparis* sp.	China	KM199330	KM199478	KM199393	–	–	–	–	–	–
Pestalotiopsis sequoiae	MFLUCC 13-0399*	*Sequoia sempervirens*	Italy	KX572339	–	–	–	–	–	–	–	–
Pestalotiopsis shaanxiensis	CFCC 54958*	*Quercus variabilis*	China	ON007026	ON005043	ON005054	–	–	–	–	–	–
Pestalotiopsis shaanxiensis	CFCC 57356	*Quercus variabilis*	China	ON007027	ON005044	ON005055	–	–	–	–	–	–
Pestalotiopsis silvicola	CFCC 55296*	*Cyclobalanopsis kerrii*	China	ON007032	ON005049	ON005060	–	–	–	–	–	–
Pestalotiopsis silvicola	CFCC 54915	*Cyclobalanopsis kerrii*	China	ON007033	ON005050	ON005061	–	–	–	–	–	–
Pestalotiopsis silvicola	CFCC 57363	*Cyclobalanopsis kerrii*	China	ON007034	ON005051	ON005062	–	–	–	–	–	–
Pestalotiopsis spathulata	CBS 356.86*	*Gevuina avellana*	Chile	KM199338	KM199513	KM199423	–	–	–	–	–	–
Pestalotiopsis spathuliappendiculata	CBS 144035*	*Phoenix canariensis*	Australia	MH554172	MH554607	MH554845	–	–	–	–	–	–
Pestalotiopsis telopeae	CBS 114137	*Protea* sp.	Australia	KM199301	KM199559	KM199469	–	–	–	–	–	–
Pestalotiopsis telopeae	CBS 114161*	*Telopea* sp.	Australia	KM199296	KM199500	KM199403	–	–	–	–	–	–
Pestalotiopsis telopeae	CBS 113606	*Telopea* sp.	Australia	KM199295	KM199498	KM199402	–	–	–	–	–	–
Pestalotiopsis terricola	CBS 141.69*	soil	Pacific Islands	MH554004	MH554438	MH554680	–	–	–	–	–	–
Pestalotiopsis thailandica	MFLUCC 17-1616*	*Rhizophora mucronata*	Thailand	MK764285	MK764329	MK764351	–	–	–	–	–	–
Pestalotiopsis trachycarpicola	OP068*	*Trachycarpus fortunei*	China	JQ845947	JQ845946	JQ845945	–	–	–	–	–	–

Pestalotiopsis trachycarpicola	IFRDCC 2403	*Podocarpus macrophyllus*	China	KC537809	KC537816	KC537823	–	–	–	–	–	–
Pestalotiopsis trachycarpicola	LC4523	*Camellia sinensis*	China	KX895011	KX895230	KX895344	–	–	–	–	–	–
Pestalotiopsis unicolor	MFLUCC 12-0276*	*Rhododendron* sp.	China	JX398999	–	JX399030	–	–	–	–	–	–
Pestalotiopsis unicolor	MFLUCC 12-0275	unidentified tree	China	JX398998	JX399063	JX399029	–	–	–	–	–	–
Pestalotiopsis verruculosa	MFLUCC 12-0274*	*Rhododendron* sp.	China	JX398996	JX399061	–	–	–	–	–	–	–
Pestalotiopsis yanglingensis	LC4553*	*Camellia sinensis*	China	KX895012	KX895231	KX895345	–	–	–	–	–	–
Pestalotiopsis yanglingensis	LC3412	*Camellia sinensis*	China	KX894980	KX895197	KX895312	–	–	–	–	–	–
Pestalotiopsis yunnanensis	HMAS 96359*	*Podocarpus macrophyllus*	China	AY373375	–	–	–	–	–	–	–	–

[1] Acronyms: ATCC: American Type Culture Collecton, Virginia, USA; BBH: BIOTEC Bangkok Herbarium, National Science and Technology Development Agency, Thailand; CBS: Westerdijk Fungal Biodiversity Institute (CBS-KNAW Fungal Biodiversity Centre), Utrecht, The Netherlands; CFCC: China Forestry Culture Collection Centre, Beijing, China; CMW: Culture collection of Michael Wingfield, University of Pretoria, South Africa; CPC: Culture collection of Pedro Crous, The Netherlands; IMI: Culture collection of the International Mycological Institute, CABI Bioscience, Egham, Surrey, UK; MFLU: Mae Fah Luang University herbarium, Thailand; MFLUCC: Mae Fah Luang University Culture Collection, Thailand; MUCC: Murdoch University Culture Collection, Perth, Australia; NE: Gerard Adams collections, University of Nebraska, Lincoln NE, USA; PPRI: Culture collection of the Plant Protection Research Institute, Agriculture Research Center, Pretoria, South Africa; XJAU: Xinjiang Agricultural University, Xinjiang, China; NA: not applicable. All the new isolates used in this study are in bold and the type materials are marked with *.
