# Supplementary figures and images for: The Potential Fungal Pathogens of Euonymus japonicus in Beijing, China
Source: J Fungi (Basel). 2023 Feb 18;9(2):271. doi: 10.3390/jof9020271 (PMC9966606; doi:10.3390/jof9020271)

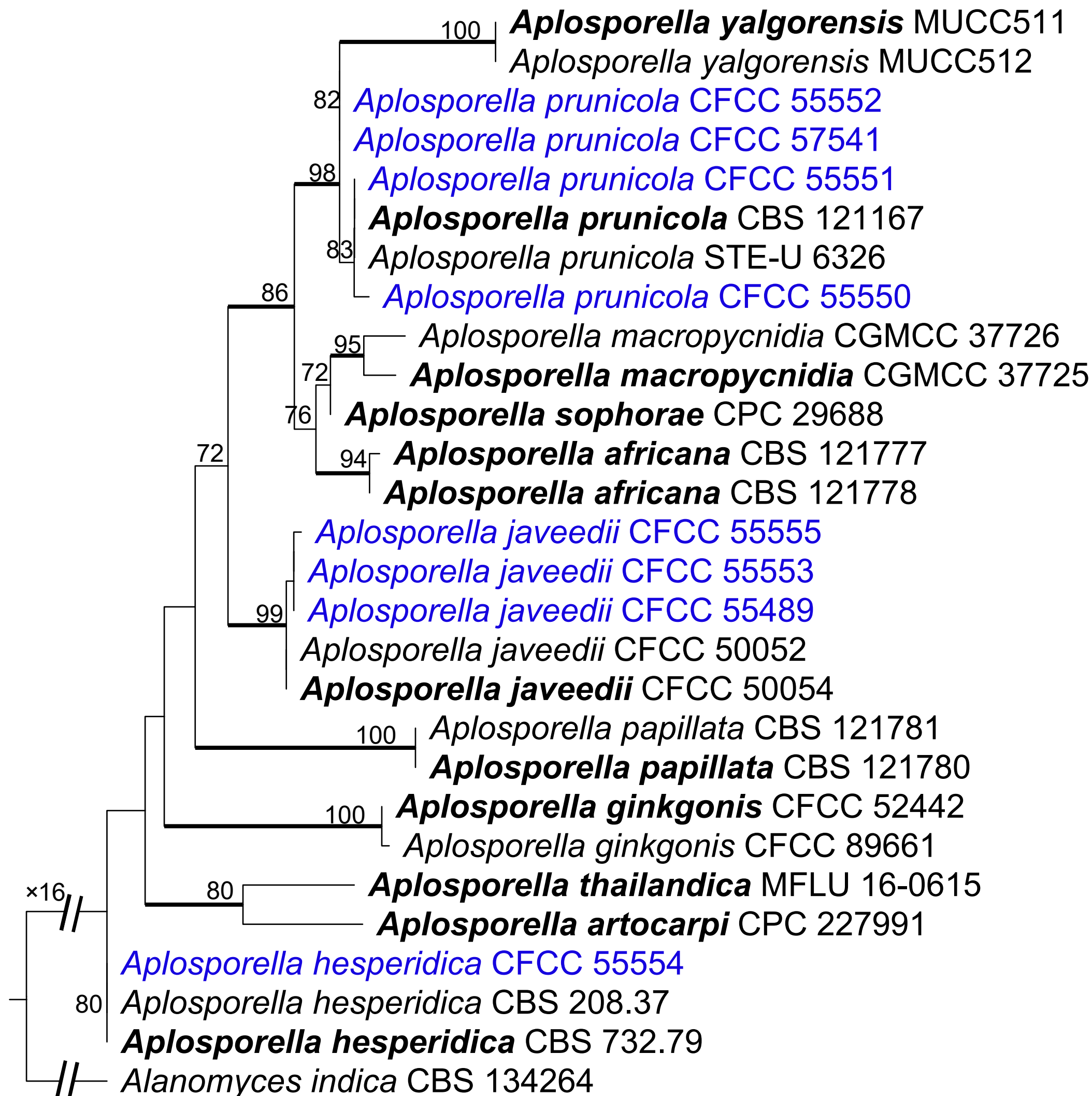

Supplement: Supplementary file 1 [file jof-09-00271-s001.zip › Figure S1.pdf]

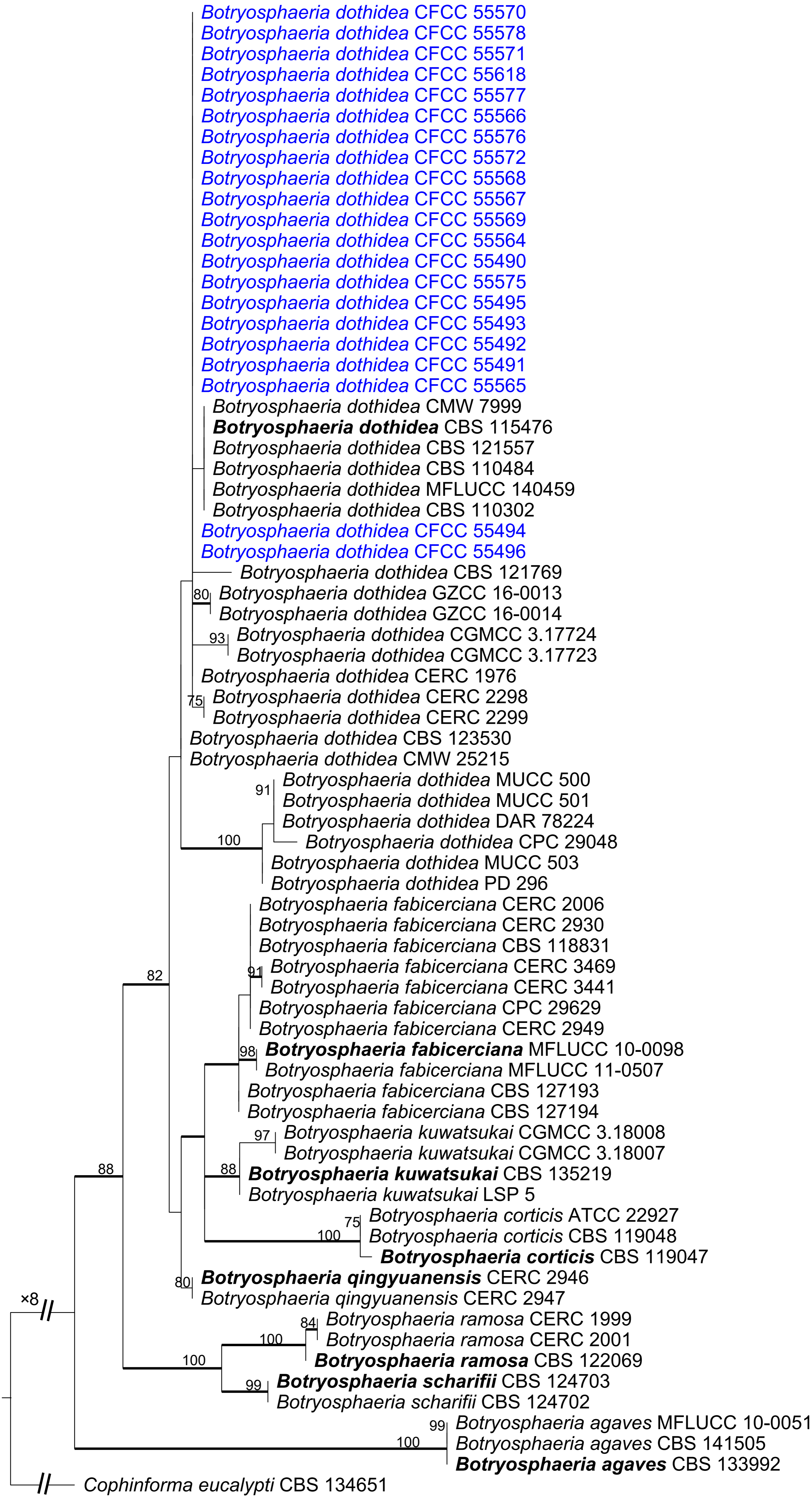

Supplement: Supplementary file 1 [file jof-09-00271-s001.zip › Figure S2.pdf]

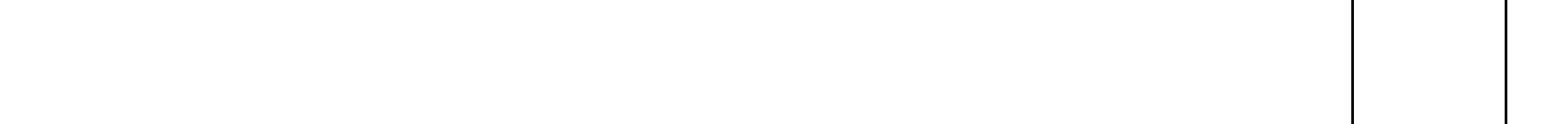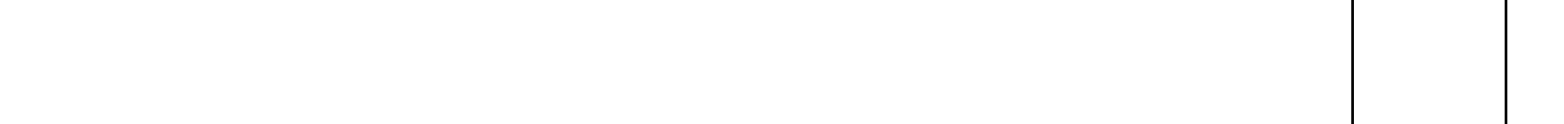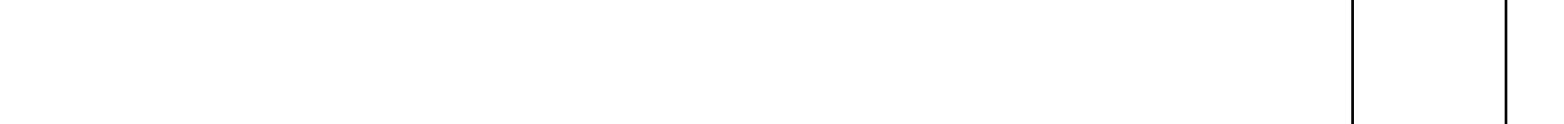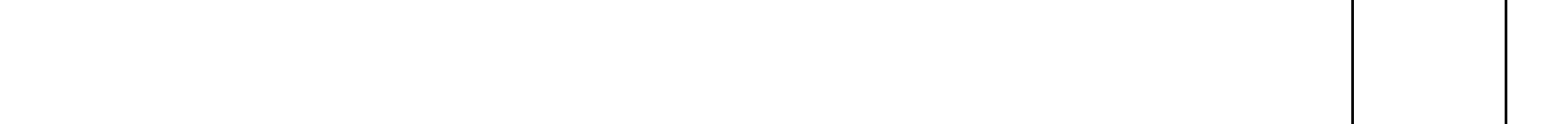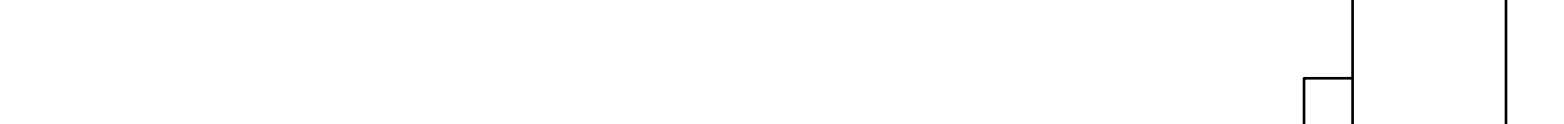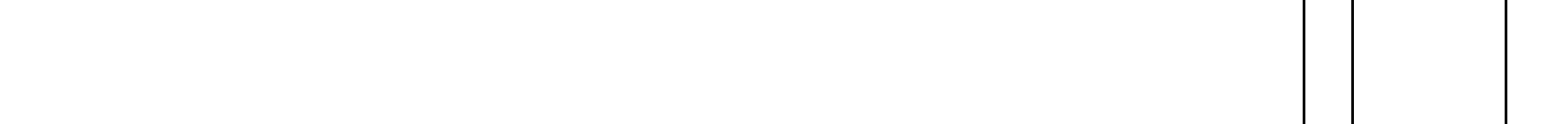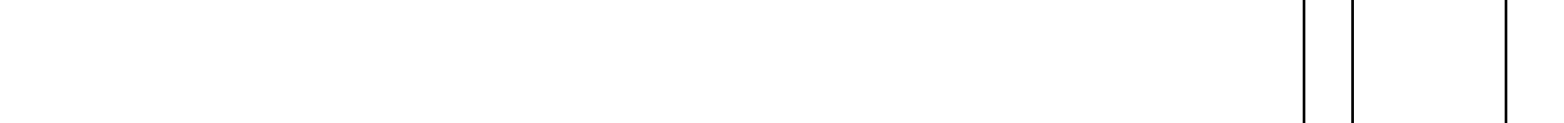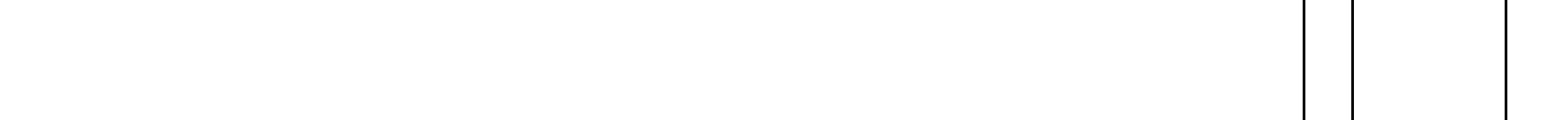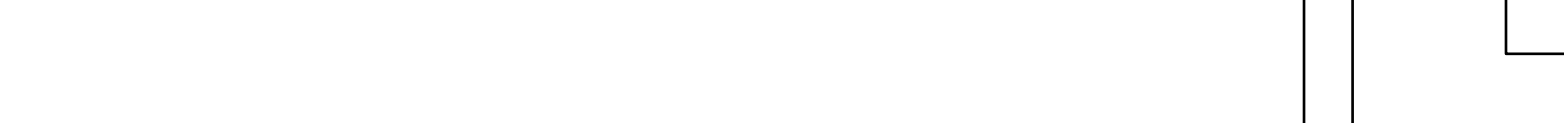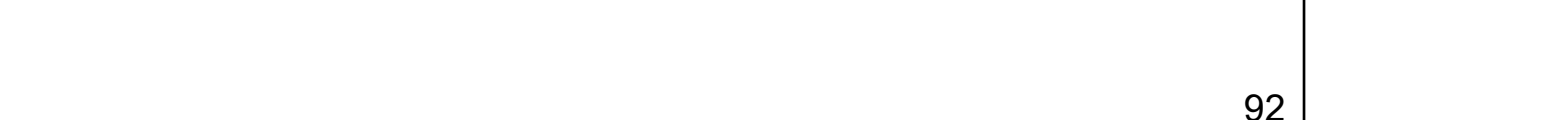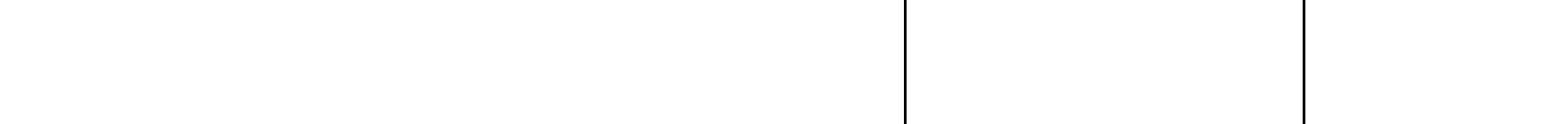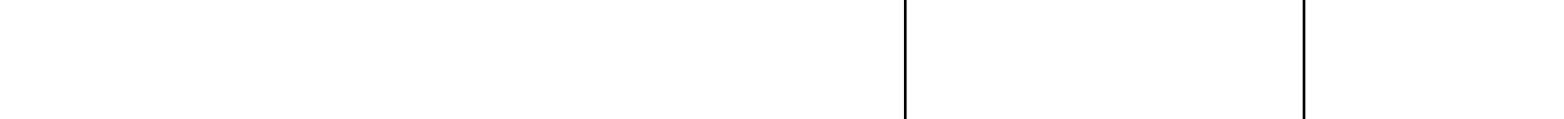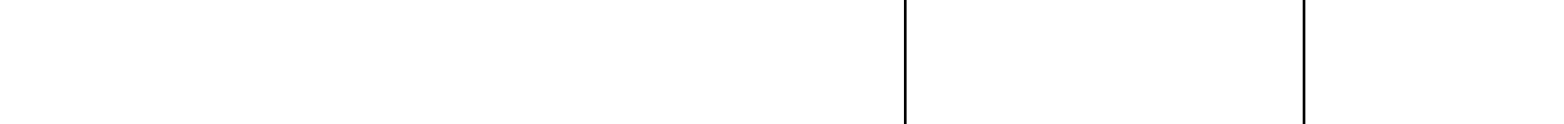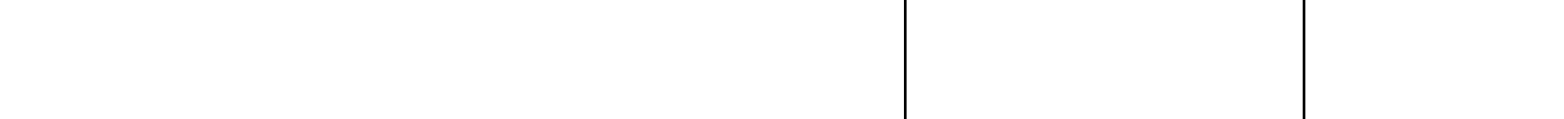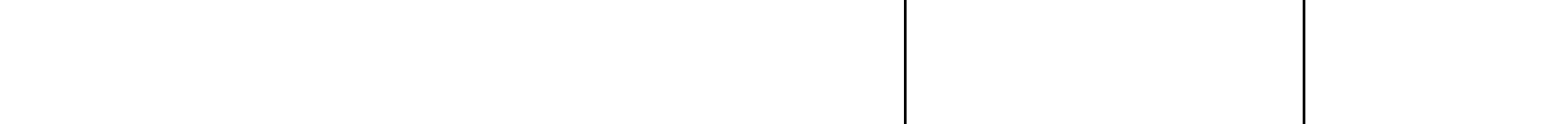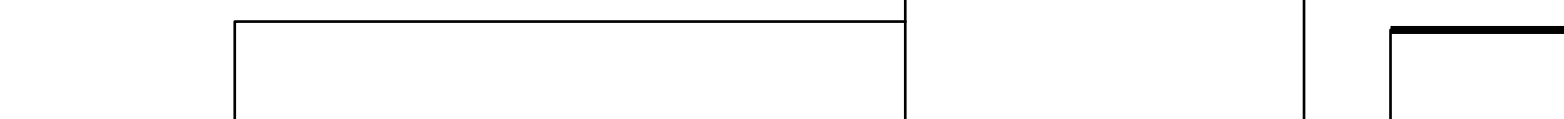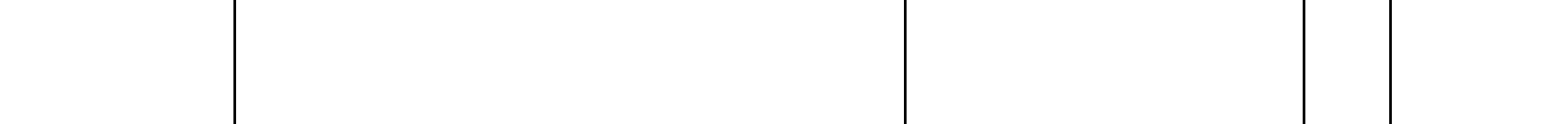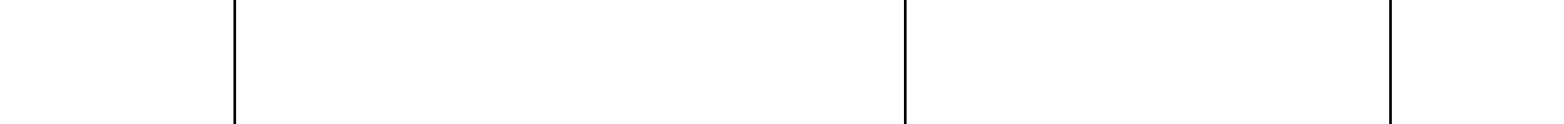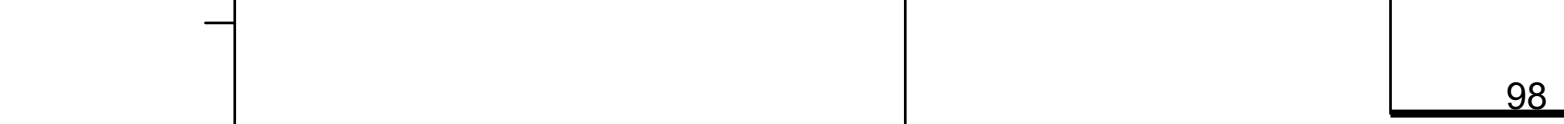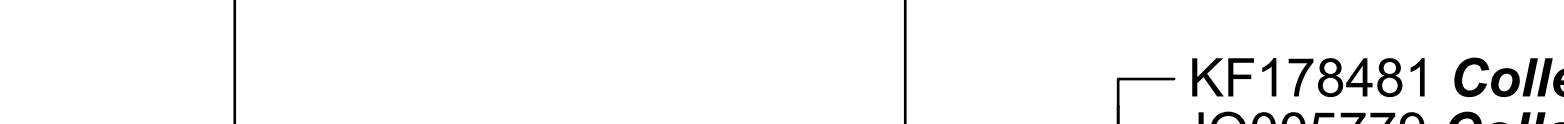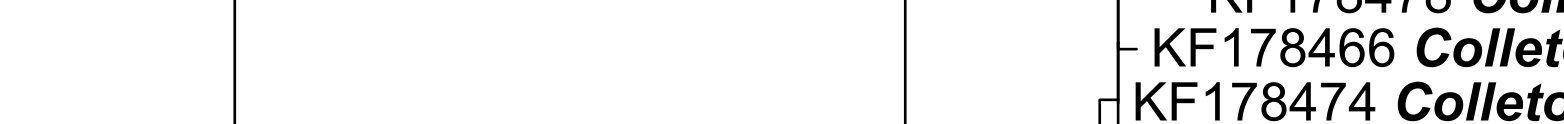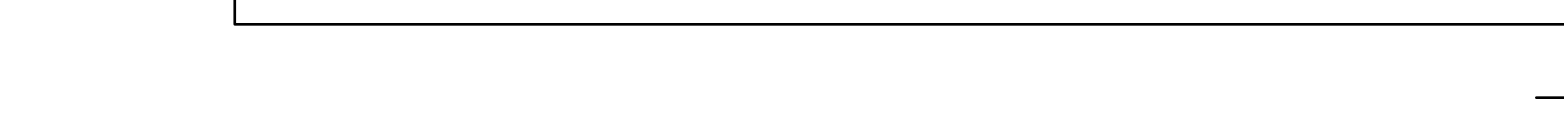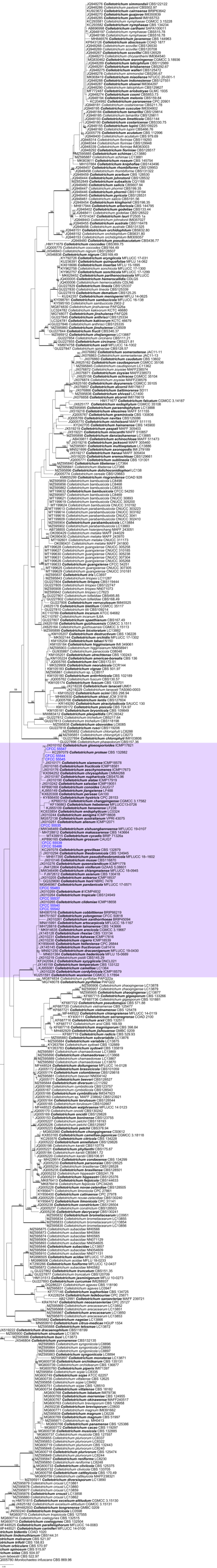

Supplement: Supplementary file 1 [file jof-09-00271-s001.zip › Figure S3.pdf]

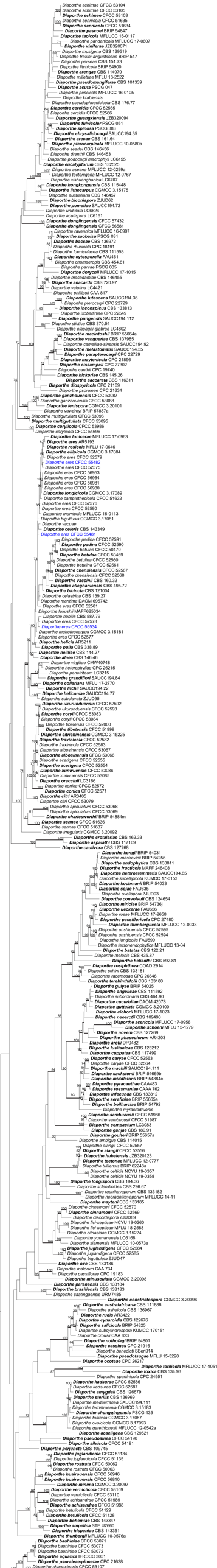

Supplement: Supplementary file 1 [file jof-09-00271-s001.zip › Figure S4.pdf]

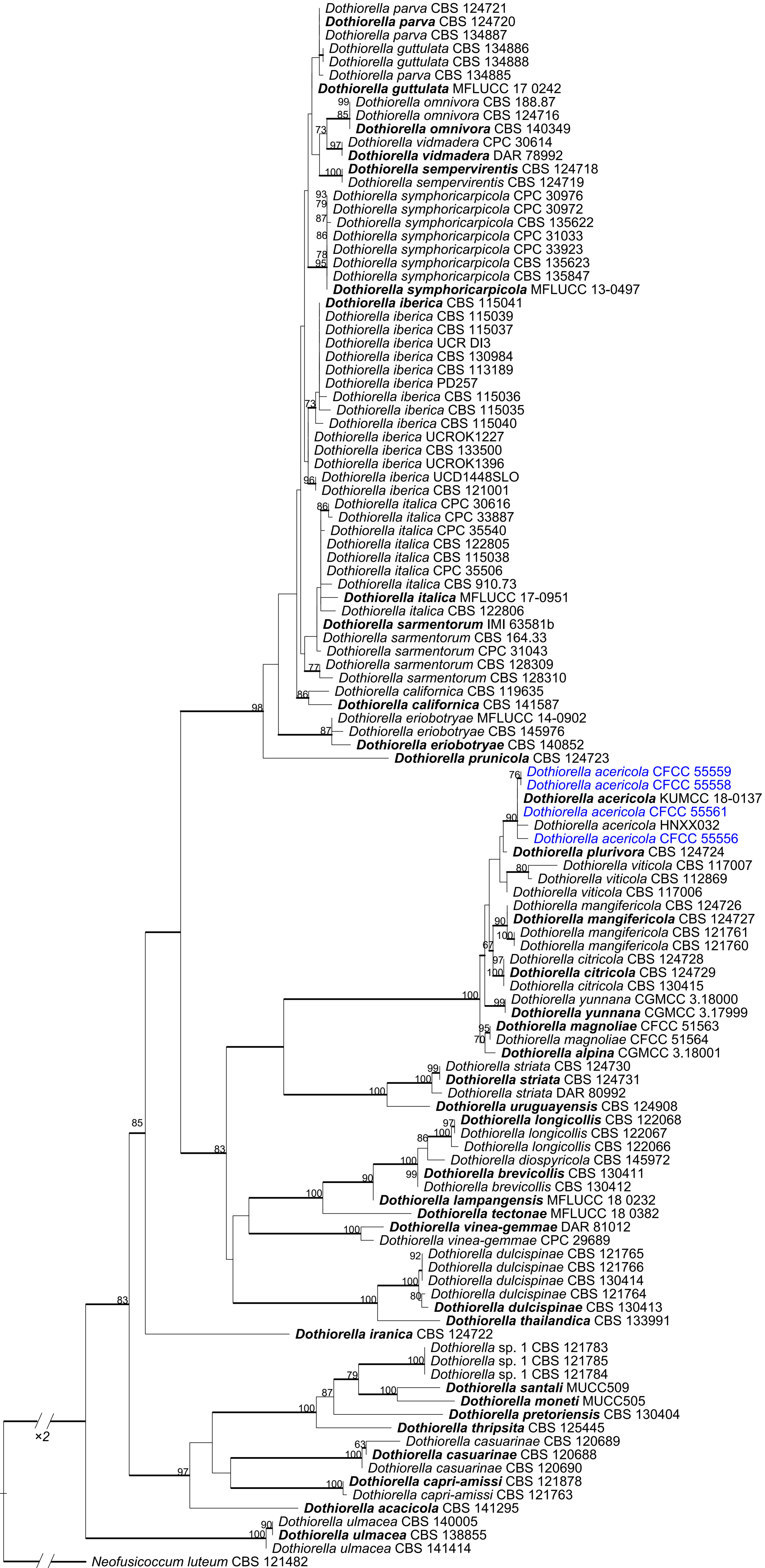

Supplement: Supplementary file 1 [file jof-09-00271-s001.zip › Figure S5.pdf]
